# Representing dynamic biological networks with multi-scale probabilistic models

**DOI:** 10.1038/s42003-018-0268-3

**Published:** 2019-01-17

**Authors:** Alexander Groß, Barbara Kracher, Johann M. Kraus, Silke D. Kühlwein, Astrid S. Pfister, Sebastian Wiese, Katrin Luckert, Oliver Pötz, Thomas Joos, Dries Van Daele, Luc De Raedt, Michael Kühl, Hans A. Kestler

**Affiliations:** 1 Institute of Medical Systems Biology, Ulm University, 89081 Ulm, Germany; 2 Institute of Biochemistry and Molecular Biology, Ulm University, 89081 Ulm, Germany; 3 Core Unit Mass Spectrometry and Proteomics, Ulm University, 89081 Ulm, Germany; 4 NMI Natural and Medical Sciences Institute at the University of Tübingen, 72770 Reutlingen, Germany; 5 Department of Computer Science, Katholieke Universiteit Leuven, 3001 Heverlee, Belgium

## Abstract

Dynamic models analyzing gene regulation and metabolism face challenges when adapted to modeling signal transduction networks. During signal transduction, molecular reactions and mechanisms occur in different spatial and temporal frames and involve feedbacks. This impedes the straight-forward use of methods based on Boolean networks, Bayesian approaches, and differential equations. We propose a new approach, ProbRules, that combines probabilities and logical rules to represent the dynamics of a system across multiple scales. We demonstrate that ProbRules models can represent various network motifs of biological systems. As an example of a comprehensive model of signal transduction, we provide a Wnt network that shows remarkable robustness under a range of phenotypical and pathological conditions. Its simulation allows the clarification of controversially discussed molecular mechanisms of Wnt signaling by predicting wet-lab measurements. ProbRules provides an avenue in current computational modeling by enabling systems biologists to integrate vast amounts of available data on different scales.

## Abstract (web summary)

Alexander Gross et al. present ProbRules, a dynamic modeling approach that combines probabilities and logical rules to represent network dynamics over multiple scales. They apply ProbRules to a Wnt network, predicting gene expression readouts that they confirm with wet-lab experiments.

## Introduction

The growth in available knowledge about interactions of genes and proteins^1^ inspired efforts to integrate this into mathematical models^2^. This was done in order to simulate functions of organisms in silico^3^ and in particular, to use the resulting insights for prediction of outcomes in vitro and in vivo^4^. The complexity of elucidating such interaction networks and their mechanisms represents an ongoing challenge^5^. Static approaches can provide a basis for assessing possible protein-protein interactions^6^. As their specific actions depend on activities of other interactions as prerequisites, the system of interest can be better understood by examining the dynamics of the underlying interactions^7–10^.

A range of dynamic modeling approaches are used for analyses of biological systems. The choice of model type is based in particular on available data. Boolean networks can represent discrete levels of system interactions’ activities which makes them especially suitable to model gene regulatory networks^11^. Regarding substance quantities and time as continuous allows one to use kinetic laws to describe the temporal dynamics. The resulting differential equations models have been used for analysis of metabolism^12^. Bayesian networks can represent distributions of interaction activities dependent on other interactions. Iteratively reusing derived distributions allows them to recapitulate dynamical systems^13^. There are also several approaches aimed at bridging discrete and continuous models, by allowing continuous times and stochastic Boolean models^14,15^, by allowing intermediate values for Boolean networks^16^, or introducing a probabilistic selection of Boolean functions^17,18^. A range of approaches is based on a logical description of a system that allows a formal verification of its properties^19–22^.

These aforementioned dynamic modeling approaches require an explicit consideration of the crosstalk of all simultaneous interactions. This can be done for example by defining precedencies or specifying outcomes of combinations. Thus, such methods imply further additional effort for the modeler. Especially, as only limited data on the effects of interactions’ combinations is available, they face further challenges in deducing appropriate model formulations (ODEs, Boolean formulae) manually as well as automatically^23–25^. In contrast, logical rules can capture the combinatorial nature of possible interactions in a more intuitive way by allowing the specification of each transition as a rule independent of all other rules^26–29^. Such rules can be implemented into mathematical models that can be simulated in-silico and analyzed using logical frameworks^30^.

Perhaps the most common setting in signaling networks is the transduction of an extracellular signal from the plasma membrane by a cascade of messengers towards a transcriptional response in the nucleus^31^. This is mediated by a set of diverse molecular reactions and mechanisms that take place in different spatial and temporal frames. In a static view, knowledge about possible interactions of components can be obtained comparatively easily as the conditions can be either controlled or averaged over a large number of combinations^6^. Under dynamics, the presence of specific preconditions for the action of an interaction can become crucial^31^. Thus, the interdependencies between the interactions define a logic succession of interaction activities whose stages are not equidistant. This constitutes a major reason for the difficulties encountered when modeling signal transduction using the previously mentioned modeling approaches. In contrast, computational analyses of cancer progression based on multiscale methods have been fruitful^32–34^. The different cell types involved in cancer are intertwined by mechanisms on multiple temporal and spatial scales, as are components of signaling networks within cells^31^.

Here, we develop a rule-based probabilistic modeling method, ProbRules, that can be used to predict the dynamic behavior of a complex signaling network, such as the computationally well investigated Wnt network^35–41^, based on qualitative data. This method is based on a more intuitive description of the involved interactions in the form of rules instead of rate equations. States of protein interactions are represented by probabilities for the occurrence of this interaction at each point in time. Successor states are derived using activities of rules based on current state probabilities.

As network motifs constitute basic building blocks of molecular networks^42,43^, they can serve as proof-of-principle applications for novel modeling methods. We show that the rule-based probabilistic method can represent various networks motifs comparable to quantitative models based on differential equations. Wnt signaling is vital in different contexts, such as during embryonic development^44^ and cancer^45^ and thus can serve as an exemplary signal transduction network for computational investigation. Important questions within the Wnt network focus on the interplay of the different branches^36^ and molecular mechanisms of β-catenin accumulation upon stimulation by Wnt^39–41^. We specify a comprehensive multi-scale rule-based probabilistic model of Wnt/β-catenin and Wnt/JNK (c-Jun N-terminal kinase) signaling based on literature. Specifically, we investigate whether the β-catenin level is inhibited at the level of β-catenin phosphorylation or ubiquitination. The computational results are confirmed by wet-lab experiments.

## Results

### ProbRules is a novel probabilistic modeling approach

**Fig. 1:**
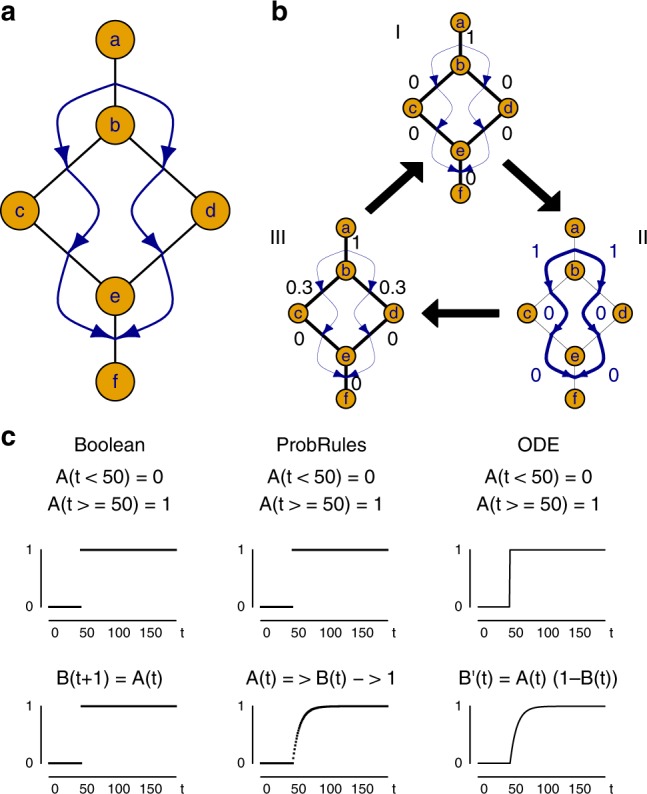
ProbRules modeling. **a** ProbRules models represent activities of interactions by probabilities on the edges of a static graph (i.e., PPIs). Rules specify interdependencies between the interactions (blue arrows). **b** The ProbRules simulation uses the specified probabilities of the edges (I, numbers) for determining the activity levels for the rules (II), and subsequently applies active rules to modify the target edge probabilities accordingly (III). Iterations of this process enable ProbRules to represent system dynamics. **c** Comparison of simple regulation dynamics specified by a Boolean model, by a ProbRules model and by an ordinary differential equation (ODE) model. Input A (upper graphs) is not present (0) before *t* = 50 and fully present (1) thereafter. Upon presence, it activates output B (lower graphs) and drives it from the initial value (0) towards presence (1)

Our rule-based probabilistic model consists of an interaction graph^46^ and a set of rules. Vertices in the graph constitute components of a system. Possible interactions among these correspond to undirected edges. The interaction graph forms the static representation of the model (Fig. 1a). Probabilities attached to the edges represent states of interactions. This is different from other approaches where the states of models correspond to the presence/absence (Boolean) or concentration (ODE) of components of the system. Rules drive target interactions’ probabilities based on logical conjunctions of source interactions towards defined values by so-called attack rates. These attack rates allow target interactions’ probabilities to take intermediate values during a transition towards a target value. Using such ProbRules models, we can represent systems dynamics comparable to analytic solutions of ordinary differential equations (Fig. 1c). Concurrent rules for a particular target interaction are combined by averaging the proposed intermediate values. We use exact probabilistic inference^47^ for deriving rules’ activities from source interactions and proposed intermediate values. This way, rules can formalize knowledge about interdependencies of interactions independent of the quantitative scales involved. Such multiple scales are predominant in signaling networks^31^. Generally, the probabilistic simulation approach works as described in Table 1 and Fig. 1a.

**Table 1:** ProbRules algorithm

Prerequisites	Components (i.e., compounds, molecules) are nodes in the graph Interactions (with probabilities) are edges between components Dependencies between interactions (rules) are curves arcs	
Algorithm	(1)	Initial edge probabilities are set
	(2)	Interactions are determined according to edge probabilities
	(3)	Determine and apply active rules
	(4)	Adjust edge probabilities
	(5)	GoTo (2) until termination condition is fulfilled

In the following we give a short mathematical description of our approach; a full account is given in the Supplementary Information.

### Static graph of possible interactions

The graph of interactions *GI* = (*V*, *E*) consists of a set *V* of vertices representing system components and a set *E* ⊆ *V* × *V *of edges representing possible interactions of the components. As such, the graph represents the static structure of the modeled system. The example shown in Fig. 1a is defined by *V* = {*a*,*b*,*c*,*d*,*e*,*f*} and *E* = {(*a*, *b*),(*b*, *c*),(*b*, *d*),(*c*, *e*),(*d*, *e*),(*e*, *f*)}.

### Model states, time points and dynamics

The state of an interaction (*i*, *j*) ∈ *E* of two components *i *∈ *V* and *j *∈ *V *at a time point t is denoted by the probability *p*_*t*_(*i*, *j*). A state *S*_*t*_(*E*) of the model for a time point *t* is defined by corresponding probabilities *p*_*t*_ attached to the edges *E* of the graph *GI*:

$$S_t\left( E \right) = \left\{ {p_t\left( {i,\,j} \right) \in \left[ {0,1} \right]|\left( {i,\,j} \right) \in E} \right\}$$

Each such *S*_*t*_ defines a random graph model which essentially is a probability distribution *D*_*t*_ over possible subgraphs *G* = (*V, E*_G_) of *GI* with *E*_*G*_ ⊆ *E *^47^. Therefore, the probability Pr(*G*) of a subgraph *G* is

$$\Pr \left( G \right) = \mathop {\prod }\limits_{e \in E_G} p_t(e)\mathop {\prod }\limits_{e \in E \setminus E_G} 1 - p_t(e)$$

The edges (*i*, *j*) can be viewed as independent random variables that are true with probability *p*_*t*_(*i*, *j*). Dynamics of a modeled system can be represented by a sequence of states *S*_0_*, S*_1_*,…, S*_*T*_. Thereby, the probabilities of the different interactions can evolve over time. Although the random variables corresponding to the edges are independent from each other at any particular point in time *t*, interdependencies between the interactions at different time points can be introduced. This is achieved in a controlled way by evaluating rules.

### Interdependency rules

A set *R* of rules defines the interdependencies between activity states of interactions. Each rule takes the form

$$r:\varphi \Rightarrow p\left( {i,\,j} \right)\mathop{\longrightarrow}\limits^{{a_r}}q$$

where *φ* represents a Boolean condition (formula) on source interaction states and *(i, j)* is the affected target interaction whose activity state *p(i,j)* is driven towards the target probability *q* by the attack rate *a*_*r*_. Interdependencies between interactions can act by driving target interactions’ probabilities towards arbitrary values using arbitrary attack rates. For the example in Fig. 1 six rules can be specified:

$$1:p\left( {a,b} \right) \Rightarrow p\left( {b,c} \right)\mathop{\longrightarrow}\limits^{{a_1}}1$$

$$2:p\left( {a,b} \right) \Rightarrow p\left( {b,d} \right)\mathop{\longrightarrow}\limits^{{a_2}}1$$

$$3:p\left( {b,c} \right) \Rightarrow p\left( {c,e} \right)\mathop{\longrightarrow}\limits^{{a_3}}1$$

$$4:p\left( {b,d} \right) \Rightarrow p\left( {d,e} \right)\mathop{\longrightarrow}\limits^{{a_4}}1$$

$$5:p\left( {c,e} \right) \Rightarrow p\left( {e,\,f} \right)\mathop{\longrightarrow}\limits^{{a_5}}1$$

$$6:p\left( {d,e} \right) \Rightarrow p\left( {e,\, f} \right)\mathop{\longrightarrow}\limits^{{a_6}}1$$

Given a model state *S*_*t*_ at time *t*, the probability of activation *φ*_*t*_ of a rule’s formula *φ* can be determined using the rules of probability calculus. Thus, *φ*_*t*_ denotes the probability that the logical formula *φ* holds in a randomly sampled subgraph according to the distribution *D*_*t*_.

The interdependency rules operate in a step-wise manner in regard to the evolution in time. At each point in time *t−1*, each rule *r* proposes a new probability value *q*_*t*_*(r, i, j)* for its target interaction *(i,j)*.

### Interaction states proposed by rules

A basic rule *r* with condition *φ*, target *(i, j)*, target probability *q*, and attack rate *a* has to be read as: whenever *φ* holds at time *t−1* the probability of the interaction *(i, j)* at time *t* will be

$$q_t\left( {r,i,j} \right) = \left( {1 - a} \right) \ast p_{t - 1}\left( {i,\,j} \right) + a \ast q$$

That means that whenever *φ*_*t-1*_ *=* *1* the new probability for *(i, j)* is an affine combination of the previous state *p*_*t−1*_*(i, j)* and the target probability *q* as determined by the attack rate *a*.

In general (for arbitrary *S*_*t*_) the condition *φ* will not hold on all subgraphs that can be sampled according to *D*_*t*_, and thus the probability *φ*_*t*_ will be < *1*. To account for this, the rules contribute only with a corresponding factor *φ*_*t−1*_ * q_*t*_*(r, i, j)* to the target *p*_*t*_*(i, j)*. Due to subgraphs where *φ*_*t*_ is false, a factor for the negation *(1−φ*_*t-1*_*)* would be lost at each time step. To account for this lost factor and subgraphs, a standard decay rule is introduced.

### Decay of interactions’ activities

The standard decay rule states that an interaction that is not affected by any active rule returns to its defined initial state *p*_0_*(i, j)* by a global decay rate *d*:

$$q_t\left( {d,i,j} \right) = \left( {1 - d} \right) \ast p_t\left( {i,\,j} \right) + d \ast p_0\left( {i,\,j} \right)$$

### Combining rules targeting an interaction with decays

Combining a single effective rule with the default decay rule yields the value

$$p_t\left( {i,\,j} \right) = \varphi _{t - 1} \ast q_t\left( {r,i,\,j} \right) + \left( {1 - \varphi _{t - 1}} \right) \ast q_t\left( {d,i,j} \right)$$

Thus the decay rule applies only when the other rule is not active.

Consider now rules *r*_*1*_*,…, r*_*n*_ with formulas *φ*_*1*_*,…,φ*_*n*_ which target the same interaction *(i,j)*. They each propose new states *q*_*t*_*(r*_*1*_*, i, j),…, q*_*t*_*(r*_*n*_*, i, j)* of that interaction. A method for combination can be obtained by considering the meaning of a rule *r*_*k*_. Such a rule basically states that whenever *φ*_*k*_ holds—for those subgraphs for which *φ*_*k*_ holds - the target has to be set to *q*_*t*_*(r*_*k*_*, i, j)*. A concrete realization consists of subgraphs for which exactly *m* conditions *φ*_*k1*_*,…, φ*_*km*_ hold with corresponding *q*_*t*_*(r*_*k1*_*, i, j),…, q*_*t*_*(r*_*km*_*, i, j)*. In these cases, the combination will be the average to these subgraphs:

$$q_t\left( {{\mathrm{\Phi }}_{\{ k1, \ldots ,km\} },i,\, j} \right) = \frac{1}{m}\mathop {\sum }\limits_{i = 1}^m q_t\left( {r_{ki},i,\,j} \right)$$

where

$${\mathrm{\Phi }}_{\{ k1, \ldots ,km\} } = \mathop { \wedge }\limits_{s \in \{ k1, \ldots ,km\} } \phi _s\mathop { \wedge }\limits_{s \in \left\{ {1, \ldots ,n} \right\} - \{ k1, \ldots ,km\} } \neg \varphi _s$$

As for the single rule case, the decay rule still applies for subgraphs in which ${\bigvee}_{i = 1}^n\varphi _i$ is false.

### Representing dynamics, inputs, and perturbations

After deriving the proposed next states *p*_*t*_ of all interactions *(i, j)* based on the previous interaction state *S*_*t-1*_ yields the new interaction state *S*_*t*_. Then, a new cycle can be started which yields new interaction states and so on, until some final time point *T* is reached. This allows to simulate dynamics of biochemical systems like the Wnt signaling networks using their static interaction graph and interdependency rules on the states of the interactions.

Inputs can be provided to a ProbRules model at specific interactions and times by specifying an explicit probability

$$p_t\left( {i,\,j} \right) = {\mathrm{fixed}}\left( {i{\mathrm{,}}\,j{\mathrm{,}}\,t} \right)$$

This also allows to investigate perturbations like inhibition and constitutive activation of a specific interaction *(i, j)* in a specified ProbRules model.

### Grounding on causal probabilistic time logic

Iterative evaluation of the rules can be used to derive sequences of new states (Fig. 1b) and thus to represent dynamics of systems by simulation of a ProbRules model. The probabilistic state sequences generated in this way correspond to relational processes that can be modeled in Causal Probabilistic Time Logic (CPT-L)^48^. This also allows a direct implementation of averaging upon the joint action of concurrent rules on a particular target interaction. Based on CPT-L, the model was implemented in ProbLog^47^, a probabilistic extension of Prolog^49^. We use these logical frameworks as theoretical and practical foundations of ProbRules (details of implementation in Supplementary Information).

### ProbRules models reproduce common network motifs dynamics

**Fig. 2:**
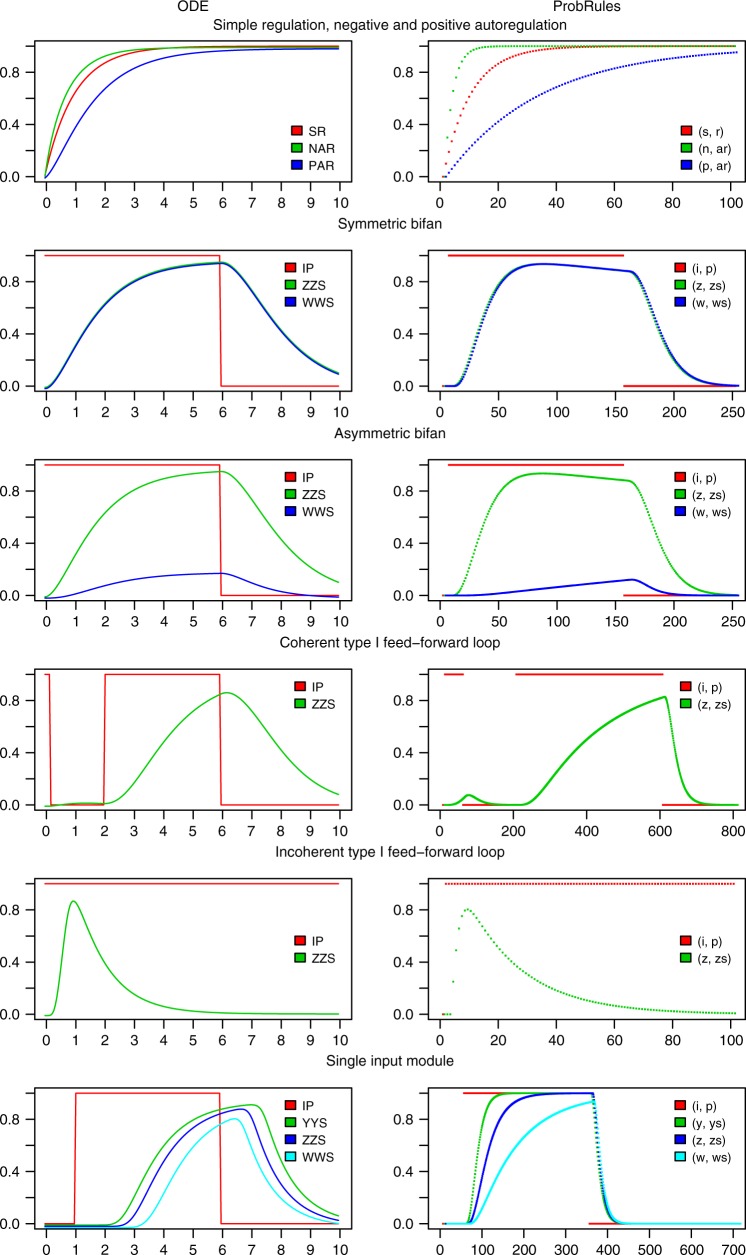
Representation of quantitative network motifs dynamics in ProbRules. Simple regulation (SR/(s, r) outputs), negative (NAR/(n,ar) outputs) and positive autoregulation (PAR/(p,ar) outputs), symmetric (ZZS/(z, zs) and WWS/(w,ws) outputs) and asymmetric (ZZS/(z, zs) and WWS/(w, ws) outputs) bifans, feed-forward loops of coherent type I (ZZS/(z,zs) outputs) and incoherent type I (ZZS/(z, zs) outputs) as well as the single input module (YYS/(y, ys), ZZS/(z, zs) and WWS/(w, ws) outputs) were modeled using ordinary differential equations (ODE, left) and ProbRules (right). Simple regulation, negative and positive autoregulation are provided with a constantly present input (not shown). All other motifs are provided by input IP in ODE models respectively (i, p) in ProbRules models. For interaction graphs, equations, and codes please see Supplementary Information

Network motifs are patterns of interconnections occurring in complex networks more often than expected in a randomly wired network^42,43^. Such patterns are assumed to form the basic building blocks of biological networks. They can enable cells to adopt specific functions such as detection of a fold-change^38,50^. We implemented simple regulation, positive autoregulation and negative autoregulation, two different bi-fan motifs, coherent and incoherent feed-forward loops and single input module motifs as rule-based probabilistic models (see Supplementary Information for interaction graphs and codes). As the function of the individual network motifs is highly dependent on the particular reactions, we used different attack rates in the individual rules to represent the local dynamics. The dynamics of the computational models match those of approaches based on differential equations (Fig. 2).

### A robust multi-scale ProbRules model of Wnt signaling

**Fig. 3:**
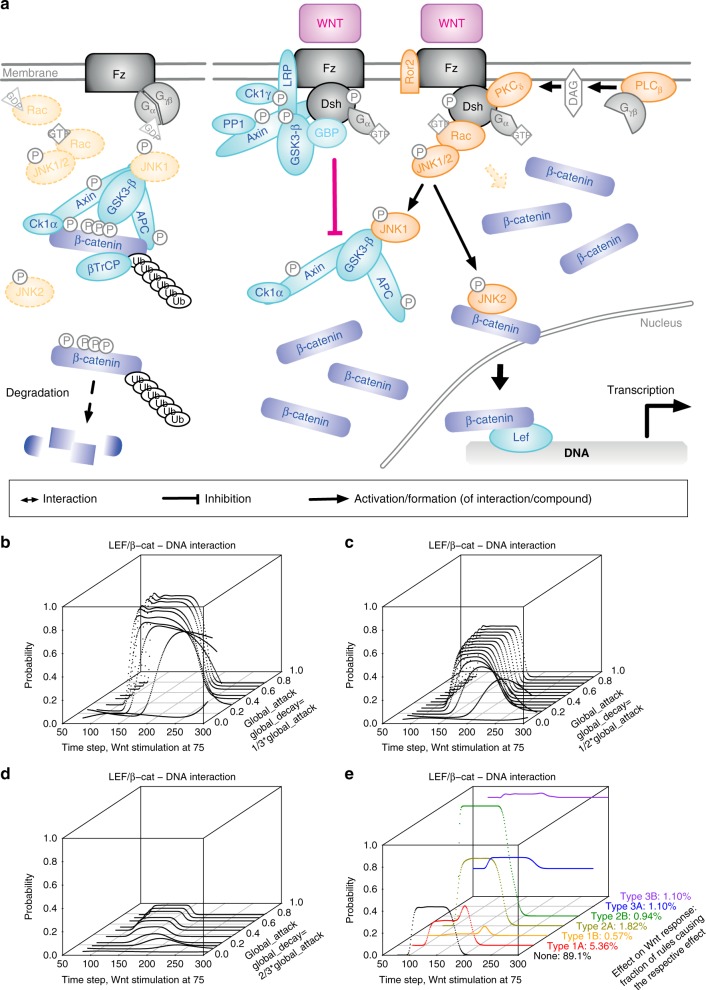
Key components and interactions in the ProbRules Wnt model. **a** Without extracellular Wnt, β-catenin is phosphorylated by the destruction complex and proteasomally degradated. Extracellular Wnt inhibits the destruction complex and cytoplasmic β-catenin accumulates. Wnt-induced disheveled activates Rac (Ras-related C3 botulinum toxin substrate) which further activates JNK1 (c-Jun N-terminal kinase 1)/JNK2. Activated JNK2 allows β-catenin to translocate into the nucleus to induce in combination with LEF (lymphoid enhancer factor) transcription. In contrast, JNK1 activates GSK3-β (glycogen synthase kinase 3β). The pale orange arrow represents a so far unknown positive influence of Rac on β-catenin accumulation that was predicted by our study. The model comprises 46 molecules with 21 logical relations for the Wnt/β-catenin and 19 logical relations for Wnt/JNK branches which were represented using 69 interaction edges and 93 rules. For details please see Supplementary Information. **b**–**d** Analyses of LEF/β-catenin–DNA interaction dynamics robustness to parameter values. Global attack rate ranges from 0.06 to 0.9. Global decay rate equals to 1/3 (**b**), 1/2 (**c**), or 2/3 (**d**) of the respective global attack rate. Global attack rate controls the onset of transcriptional response. Global decay rate determines the overall level of the response. **e** Structural robustness analysis by systematic introduction of additional rules. Rules source from all 69 interactions in the ProbRules model plus a constantly active interaction, target 67 interactions (i.e., excluding the two inputs) and drive the targets towards either ‘on’ or ‘off’, resulting in 70*67*2 = 9380 augmented models. The majority of the simulation results (89.1%) shows no or a negligible effect, around 5.93% show a moderate (Type 1A) to nearly total (Type 1B) decrease at the output, around 2.76% a moderate (Type 2A) to strong (Type 2B) increase and around 2.2% show phases of constant activation before stimulation (Type 3A) or during the complete simulation (Type 3B)

**Fig. 4:**
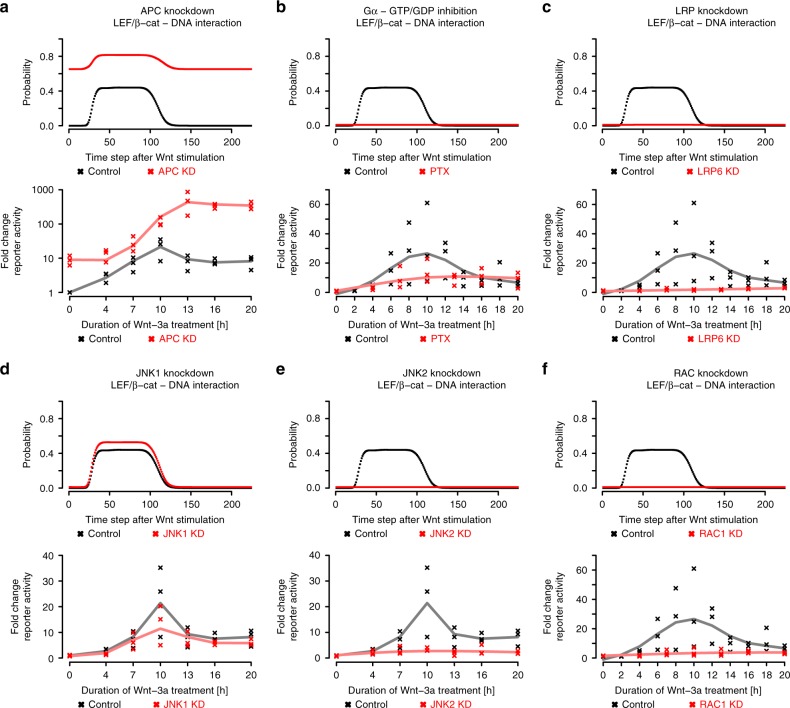
ProbRules predictions for Wnt model (upper parts of panels) and wet-lab validation (lower parts of panels). **a** Knockdown of APC strongly enhances basal as well as Wnt-3a induced Tcf/Lef transcriptional activity. **b** Inactivation of Gi, Go, and Gt α subunits reduces Wnt-3a induced Tcf/LEF transcriptional activity strongly. **c** In contrast, knockdown of LRP6 suppresses Wnt-3a induced Tcf/Lef transcriptional activity almost completely. Loss of JNK1 (**d**) does not notably affect the transcriptional response to Wnt-3a treatment whereas loss of JNK2 (**e**) clearly suppresses Wnt-3a induced Tcf/Lef transcriptional activity. **f** Furthermore, knockdown of RAC1 also strongly suppresses Wnt-3a induced Tcf/LEF transcriptional activity. Supplementary Figures 1 and 2 show RT-PCR analyses after treatment with different stealthRNA duplexes specific for APC (**a**), LRP6 (**c**), JNK1 (**d**), JNK2 (**e**), and RAC1 (**f**). Gα (**b**) inhibition was simulated by fixing the interaction probability between Gα and GTP to 0. Knock downs of APC (**a**), LRP6 (**c**), JNK1 (**d**), JNK2 (**e**) and RAC1 (**d**) were modeled in ProbRules by fixing all probabilities of interactions involving these to 0. Lower panels show fold change in Luciferase activity compared to cells treated with control siRNA: black points represent three independent control experiments, red points represent three independent perturbation experiments, and solid lines represent cubic splines fitted to the measurements

In order to assess the suitability of ProbRules models for representing an elaborate biological system, we collected core components and interactions of the Wnt signal transduction network (Fig. 3a) as it is a prototypical signal transduction subsystem with various functions in many organisms^36,39–41,44,45^. Under unstimulated conditions, β-catenin concentration within the cell is kept low by phosphorylation via the so-called destruction complex including APC (adenomatous polyposis coli), Axin and GSK3 (glycogen synthase kinase 3β). After stimulation by extracellular Wnt, this action of the destruction complex is inhibited, β-catenin accumulates in the cytoplasm, enters the nucleus and induces the transcription of target genes. Based on literature, we identified 21 logical relations in the Wnt/β-catenin branch and 19 logical relations in the Wnt/JNK branch involving 46 components and 69 interactions among them. These incorporate functional studies of various reaction types, species, and cell types found to be implicated in Wnt signaling using a range of measurement methods. We translated the logical relations into a set of 93 rules (see Supplementary Information) resulting in a comprehensive ProbRules model of Wnt signaling. This model also incorporates feedbacks which we provide in the Supplementary Information. We modeled an initial unstimulated phase during which interactions between APC, Axin, and GSK3 stabilize the destruction complex, followed by external Wnt stimulation and a slow decay to the unstimulated state. Besides an explicit attack rate for the slow Wnt stimulation decay all rules describing interdependencies between interactions within the ProbRules model of the Wnt network used a joint attack rate. Moreover, the ProbRules model of Wnt signal transduction network exhibited a remarkable robustness to a wide range of global attack and decay rates (Fig. 3b–d). We selected the global attack rate as 0.6 and the global decay rate as 0.3 based on the robustness of the model over large ranges of these parameters. Furthermore, we investigated the robustness to additional rules by systematically adding rules from all specified interactions plus an always active input to all non-input interactions for two target values (on and off) which resulted in 70*67*2 = 9380 ProbRules models. About 89% of them showed dynamics comparable to the original model of the Wnt signaling network (Fig. 3e). We contrasted the ProbRules model dynamics to measurements of LEF (lymphoid enhancer factor) transcription factor activation in cells under unperturbed and various siRNA mediated knockdown conditions using a TCF (T-cell factor)/LEF responding luciferase reporter as a readout. As expected we found a dramatic decrease in reporter gene activity upon loss of APC or LRP6 (lipoprotein receptor-related protein 6). Similar, inhibiting G protein function by pertussis toxin treatment (PTX) affected Wnt signaling. Also, loss of Rac1 (Ras-related C3 botulinum toxin substrate 1) or JNK2 affected the TCF/LEF reporter whereas a loss of JNK1 had only mild effects, likely due to low abundance of JNK1 (Fig. 4a–f, and more details in the Supplementary Figures 1, 2) Thus, we concluded that the model predictions are in accordance with wet-lab results.

### Evaluation of β-catenin accumulation upon Wnt stimulation

**Fig. 5:**
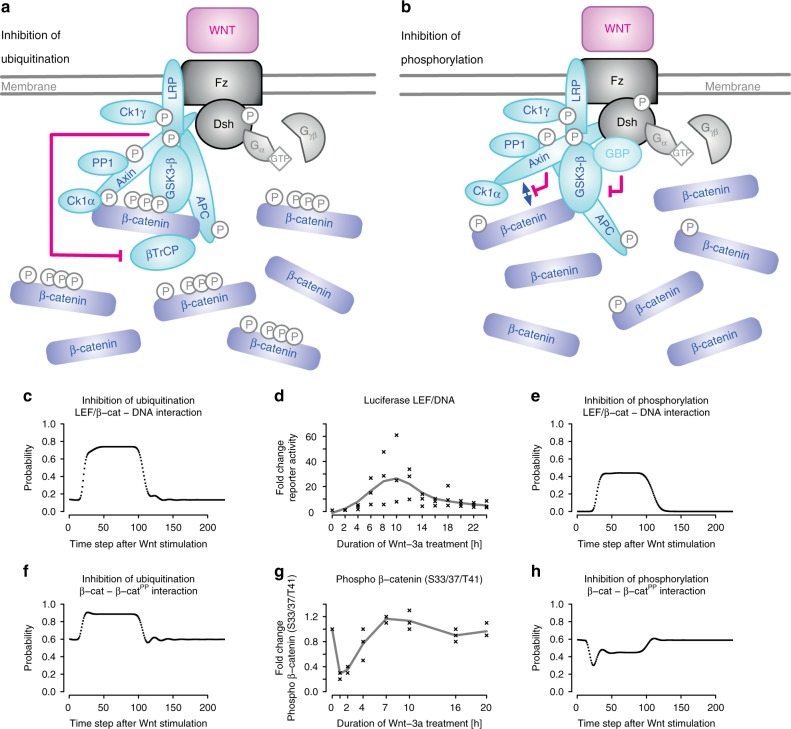
Assessment of two discussed mechanisms of destruction complex inhibition upon Wnt stimulation. **a** Inhibition of β-catenin ubiquitination^39^ prevents proteasomal degradation of β-catenin and leads to β-catenin accumulation. **b** Inhibition of β-catenin phosphorylation through inhibition of GSK-3β by GBP/FRAT^66^ or through axin dephosphorylation by protein phosphatase 1 (PP1) inhibiting axin/β-catenin interaction^40^ leads to accumulation of β-catenin. **c**, **e** Simulation of the LEF/β-catenin–DNA interaction dynamics shown as a measure for the transcriptional activity. **f**, **h** Simulation of β-catenin– β-catenin^PP^ dynamics shown as a measure for β-catenin phosphorylation. **d** Luciferase reporter activity in HEK293 cells is maximal about 8–12 h after addition of Wnt-3a while the amount of S33/S37/T41-phosphorylated β-catenin is minimal about one hour after addition of Wnt-3a and then returns to its initial level within three to four hours (**g**). **d**, **g** show fold change in Luciferase activity: black points represent three independent experiments, and solid lines represent cubic splines fitted to the measurements

Using the established model, we addressed whether the Wnt signal is transduced at the level of ubiquitination^39^ or at the level of phosphorylation of β-catenin^41^. Therefore, we integrated these mechanisms (Fig. 5a, b) into our ProbRules model of the Wnt network, simulated these and compared dynamics of β-catenin interactions (Fig. 5c, e, f, h) to results from wet-lab experiments (Fig. 5d, g) obtained using a well-characterized and validated suspension bead array-based β-catenin assay^51–55^. The results are in good agreement with published data^38^. We observed a transient drop in the amount of phosphorylated β-catenin if we included inhibition of β-catenin phosphorylation but not if we included inhibition of β-catenin ubiquitination into the model (Fig. 5f, h). Thus, our ProbRules model of Wnt signaling and our experimental (Fig. 5d, g) data provide further support for the signal transduction mechanism through inhibition of β-catenin phosphorylation^41^.

## Discussion

ProbRules is a novel probabilistic modeling approach for integrating multi-scale knowledge about the dynamics of interactions. It mitigates the costs of an investment into specifying an in-silico model of a biological system in several ways. First and foremost, the domain expert can focus on knowledge representation by rules as the translation into a mathematical model is done automatically using exact probabilistic inference^56^ methods used by the ProbRules implementation^47^. As with other approaches, modelers might start building a rough model with only core interactions, comparatively few rules connecting these and global rates without having to consider all possible effects of combinations of interactions. Such models can show merely qualitative dynamics, see the ProbRules model of the Wnt signaling network specified above. ProbRules also facilitates to implicitly integrate unknown reaction partners and conditions into rules at the early stages of a model by specifying placeholders. Later, the model can be gradually refined by additional rules, adjacent interactions, and distinct rates for rules eventually approaching quantitative dynamics like we demonstrated this for network motifs. Furthermore, ProbRules enables to incorporate mechanisms with diverse scales and speeds into a unified computational model without forcing the modeler to consider relations of temporal and spatial properties of subsequent interactions. Such mechanisms are prevalent in many biomedical domains, and particularly in signal transduction networks^31^.

Technically, ProbRules is based on probabilistic programming, an emerging subfield of artificial intelligence. Network models are derived from the static graph of possible interactions. Their activities are abstracted by probabilities. The corresponding product distribution defines a state of the system on the interaction graph. Dynamics are implemented by iteratively applying rules that modify target interactions’ probabilities. Probabilistic inference is used to drive interaction activities towards target probabilities.

In order to demonstrate capabilities of ProbRules, we implemented common network motifs of biological networks. Their dynamics were comparable to results of approaches based on differential equations. Furthermore, we provided a robust comprehensive model of Wnt signaling that highlights the integration of multi-scale knowledge from literature. This ProbRules Wnt model also allowed the investigation of controversially discussed mechanisms within the Wnt signal transduction network as our wet-lab experiments confirmed the predictions of the in-silico model. The ProbRules model of Wnt signaling considered here used global parameters for the rules and thereby represented at least three different temporal scales without additional work of the modelers. A refined ProbRules model can introduce spatial scales by specifying components in several compartments. Other approaches also allow specification of mechanisms on diverse scales^23^. ProbRules differs in this respect, as it facilitates to capture dynamic prerequisites of interactions as these are predominant in multi-scale contexts like signal transduction networks^31^.

ProbRules offers a probabilistic interpretation of the specified rules. It uses a default combination scheme for avoiding a combinatorial explosion in the specification of these rules. Together with the iterative state update this results in a model that is continuous in state space and discrete in time. The intermediate rates specified in rules and the resulting multi-stage transitions of activities between interaction are an outstanding feature of ProbRules. Other models like Boolean networks, that are discrete both in time and state space, can as well be approximated by specifying 0 or 1 as attack and decay rates. Using this approximation, we observed that all network motifs except for the incoherent feed-forward loop lost the ability to represent their dynamics in comparison to the ProbRules models with intermediate rates (for details see Supplementary Information). The same applies to our ProbRules model of the Wnt signaling network.

On the other side, a direct representation of interaction probabilities by products of concentrations can possibly be obtained for some given numerical values. From a computer scientists point of view, the decision whether there are feasible decompositions of arbitrary interaction probabilities into concentrations of the partners is considered a hard problem^57^. Additionally, a method for translation of rules and their combinations into differential equations would be required in order to derive models continuous both in state space and time. Therefore, the specification of differential equations for ProbRules models faces several challenges that require a more careful consideration in future attempts.

As exemplified with the presented model of Wnt signaling, ProbRules can integrate multiple scales of systems in a single model. At least three different scales were specified for the ProbRules model of Wnt signaling (see Supplementary Information). Nevertheless, the model is able to reproduce wildtype and perturbation dynamics using a small set of global parameters that do not differ by several orders of magnitude. This demonstrates the applicability of ProbRules for specification of an initial model of a signaling network.

In contrast to Probabilistic Boolean Networks^17,18^, ProbRules uses the same rules for each transition. As the state of a model is probabilistic, so are the rules’ activities. ProbRules integrates these rules’ activities into a single new probabilistic state. This allows an evolution of model states and thus, representation of system dynamics. Boolean Network Extension^16^ (BNE) aims at uncovering additional attractors corresponding to observed phenotypes by using existing Boolean models and allowing intermediate values for component states. An approach similar to the last, Boolean Kinetic Monte-Carlo^14,15^ (BKMC), focuses on also enabling continuous time. Both BNE and BKMC require the modeler to specify Boolean formulae that have to explicitly define the relations between all factors influencing a target value. Data about the combined effects of all possible inputs on a target is usually not available due to the combinatorial explosion and thus costs. Therefore, the specification of rules independent of each other utilizing available knowledge about interdependencies of interactions like it is done in ProbRules can be considered as a fruitful direction of computational modeling research.

ProbRules provides a basis for further interpretations of the rules in a similar way as this has been described before in approaches based on non-probabilistic rules^19,26,28–30,58,59^. Besides distinct model types with combinations of discrete and continuous state or time, novel semantics for the rules can explicitly implement spatial mechanisms like compartments, diffusion and membrane passages. As stochastic effects also play an important role in biological systems like signal transduction networks, new model types can also specify ensembles of representatives in order to approach the dynamics of corresponding phenomena. Moreover, based on available static data about interactions, new dynamic rules can be derived automatically in order to obtain model dynamics comparable to observations of phenotypes, their development, and homeostasis. This resembles structure learning methods^19–21,60^, and thus techniques for combinatorial inference of rules can become available for models built on ProbRules. The integration of ProbRules with tools for visualization and specification of computational models^61^ can further lower the burden for the domain expert.

Previous modeling approaches often suffered from an explosion of parameters that have to be estimated as the effect for every combination of interactions had to be considered. Targeting this challenge, ProbRules can provide an efficient factorization of combined interactions’ effects by introducing rules into these models. This can be especially useful for Bayesian networks, as the difficulties in parameter learning impede the application of traditional approaches based on distributions of probabilities. ProbRules addresses the increasing demand in formalization and analysis of dynamic computational biology by enabling a fine-grained control over the level of mechanism specification for incorporation of succinct models. Thus, ProbRules supports the shift in the focus from metabolism and gene regulation towards complex signal transduction networks in contemporary life sciences research.

## Methods

### Cell culture

Culturing of HEK293 cells was done in Dulbecco’s modified Eagle’s medium (DMEM) supplemented with 10% fetal bovine serum (GIBCO) at 37 °C in a 5% CO_2_ incubator. For generation of HEK293 clones stably expressing a Tcf/Lef-dependent luciferase reporter, cells were transfected with 0.8 µg pGL4.18-Tcf plasmid using Lipofectamine2000 (Invitrogen). The pGL4.18-Tcf plasmid was derived from the promotorless pGL4.18 plasmid (Promega) by insertion of 7 Tcf/Lef binding sites and a TATA minimal promotor into the MCS. Two days after transfection cells were selected in culture medium supplemented with 800 μg/ml of G418 (PAA). Three independent clones resistant to G418 were propagated and the one with the highest luciferase expression was used for all following experiments.

### stRNA knockdown

For the knockdown experiments pre-designed stealthRNA duplexes (=stRNA) from Invitrogen were used targeting human LRP6 (HSS106153, 106154, HSS106155), APC (HSS100547, HSS100548), RAC1 (VHS40447, VHS40448), JNK1 (=MAPK8) (VHS40722, VHS40724) and JNK2 (=MAPK9) (VHS40726, VHS40729) (stealthRNA sequences are given in Supplementary Methods). AllStars Negative control siRNA (Qiagen) was used as a control (sequence see Supplementary Methods). HEK293T cells were seeded in 6-well plates and on the next day treated with 10 nM stealthRNA by using LipofectamineRNAiMAX reagent (Invitrogen) according the manufacturer’s protocol. All stealthRNAs were checked for their knockdown efficiency on RNA level (Supplementary Figures 1, 2) and the best for each gene was used for further analysis. For western-blot and mass-spectrometry analysis, protein was isolated after 48 h of stealthRNA treatment. The knockdown efficiency of the best stealthRNA was further confirmed on protein level (Supplementary Figures 1, 2). For the Luciferase assay HEK293 cells expressing the Tcf/Lef-dependent luciferase reporter were treated with recombinant Wnt-3a (R&D Systems) at a concentration of 50 ng/ml for 0–20 h on the third day after addition of stealthRNA. In this case, the total duration of stealthRNA treatment was either 58 h (0–10 h of Wnt-3a treatment) or 72 h (12–20 h of Wnt-3a treatment).

### Western blot

Knockdown on protein level was checked by Western blot in case of APC and LRP6 (Supplementary Figures 1A, 1B, 2A, 2B). After 48 h of treatment with stealthRNAs total protein was extracted from HEK293T cells. Therefore, cells were lysed with RIPA buffer (50 mM Tris-HCl, pH 8.0, 150 mM NaCl, 1% Nonidet P-40, 0.1% SDS, 0.5% sodium deoxycholate) and incubated on ice for 10 min. After 15 min of centrifugation at 13,000 rpm and 4 °C, supernatant was collected. The protein concentration was measured using Bradford assay with BSA as standard. Standard procedures were used for Western Blotting. Nitrocellulose membranes were incubated for 4.5 h at room temperature with primary antibodies APC [ALi 12-28] (ab58, abcam) and LRP6 (C47E12) (#3395, Cell Signaling). As loading control blots were probed with a GAPDH (14C10) (#2118 S, Cell Signaling) antibody. Proteins were visualized using Li-COR ODYSSEY Imager.

### Mass spectrometry

Knockdown on protein level was checked by mass spectrometry in case of Rac1, JNK1, and JNK2 (Supplementary Figures 1C–1D). Sample preparation: HEK293T cells were treated for 48 h with stealthRNAs, suspended in PBS and centrifuged for 5 min at 1100 rpm. Supernatant was discarded and cell pellets were sent on dry ice to mass spectrometry. Proteins were separated using standard 12.5% SDS-Page followed by colloidal Coomassie staining. Two gel slices were cut at 21 kDa and 48 kDa for Rac1, JNK1, and JNK2, respectively. Individual pieces were washed by alternating incubation in 50 mM ammonium bicarbonate and 25 mM ammonium bicarbonate / 50% Acetonitrile (ACN) thrice for 10 min each. Following vacuum drying, samples were reduced with 5 mM DTT (AppliChem, Darmstadt, Germany) for 20 min at RT and subsequently alkylated with iodoacetamide (SigmaAldrich, St. Louis, USA) for 20 min at 37 °C. After a second vacuum drying step, proteins were subjected to tryptic digest overnight at 37 °C. Peptides were extracted in two rounds by adding 20 µl 0.1% Trifluoroacetic acid (TFA)/50% ACN and incubation in an ultrasonic bath for 10 min each. ACN was evaporated and samples filled to 15 µl with 0.1% TFA.

MS-analysis: Samples were measured using an LTQ Orbitrap Velos Pro system (Thermo Fisher Scientific, Bremen, Germany) online coupled to an U3000 RSLCnano (Thermo Fisher Scientific, Idstein, Germany) as described in Mohr et al.^62^, with the following exceptions: Separation was carried out using a binary solvent gradient consisting of solvent A (0.1% FA) and solvent B (86% ACN, 0.1% FA). The column was initially equilibrated in 5% B. In a first elution step, the percentage of B was raised from 5 to 15% in 5 min, followed by an increase from 15 to 40% B in 30 min. The column was washed with 95% B for 4 min and re-equilibrated with 5% B for 25 min.

MS data analysis and statistics: Database search was performed using MaxQuant Ver. 1.5.2.8 (www.maxquant.org)^63^. For peptide identification, MS/MS spectra were correlated with the UniProt human reference proteome set (www.uniprot.org) employing the built-in Andromeda search engine^64^. Carbamidomethylated cysteine was used as a fixed modification along with oxidation (M), and acetylated protein N-termini as variable modifications. False Discovery rates were set on both, peptide and protein level, to 0.01. Calculated intensity values for JNK2 and Rac1 were normalized to the summed intensity in the respective band to correct for gel loading differences.

### RNA isolation and PCR

RNA was isolated and PCR was performed to check the knockdown efficiency of the stealtRNAs (Supplementary Figures 1, 2C–2G). After 48 h of stealthRNA treatment total RNA was extracted from the cells by using the QIAshredder™ Kit and the RNeasy® Mini Kit (QIAGEN) according to the manufacturer’s protocol. Subsequent DNAse I digestion was performed with DNAse I recombinant, RNAse-free (Roche) for 30 min at 37 °C followed by an inactivation at 65 °C for 5 min. cDNA synthesis was done with Random Hexamer Primer (ThermoFisher) and SuperScript™ II Reverse Transcriptase (ThermoFisher). This cDNA was used in the subsequent PCR, here the following program was used: 95 °C for 45 sec followed by 27 or 35 cycles of 95 °C for 15 sec/ 55 °C for 30 sec/ 72 °C for 4 sec followed by a final extension at 72 °C for 10 sec. Primers against human LRP6, APC, Rac1, JNK1, and JNK2 were used as well as human GAPDH as controls (sequences are given in Supplementary Methods). PCR products were made visible on 1% agarose gels with Midori Green and UV-illumination.

### Pertussis toxin treatment

HEK293 cells expressing the Tcf/Lef-dependent luciferase reporter were seeded in 48-well plates. On the next day 50 ng/ml pertussis toxin (PTX) (Alexis) was added. On the next day cells were treated with recombinant Wnt-3a protein (R&D Systems) at a concentration of 50 ng/ml for 2 to 20 h while the toxin was still present in the medium. The total duration of pertussis toxin treatment was either 24 h (up to 10 h of Wnt-3a treatment) or 38 h (more than 10 h of Wnt-3a treatment).

### Luciferase assay

After treatment with stealthRNA or Pertussis toxin alone or in combination with Wnt-3a, the Luciferase Assay System from Promega was used. Lysation of cells was done in 50 µl of Reporter Lysis Buffer (Promega) and cells were frozen overnight at −80 °C. For the luciferase measurement 10 µl cell lysate was added to 40 µl luciferase substrate (Promega) and the light intensity was measured in a tube Luminometer (Bertold). Afterwards the protein concentration of the lysates was measured by Bradford Assay: in 96-well-plates 100 µl of Bradford reagent were added to 2 µl of cell lysate, after 5–10 min absorbance at 595 nm was measured using a plate reading photometer (Biorad) and protein concentration calculated by use of a calibration curve. Luciferase measurements were normalized to the protein content of the cell lysates to account for possible differences in cell numbers between the different treatments and replicate experiments. Additionally, the normalized luciferase measurements were divided by the values of the untreated (i.e. without PTX) respectively control siRNA-treated samples to yield fold-changes.

### Suspension bead array-based β-catenin assay

After treatment with stealthRNA alone or in combination with Wnt-3a, suspension bead array-based β-catenin assays were performed as described by Luckert et al. to assess changes in the amount of free or phosphorylated β-catenin^51–55^.

### Code availability

A comprehensive description of the mathematical background of the modeling method used here is given in the Supplementary Information. Interaction rules for the Wnt/β-catenin and Wnt/JNK signaling were extracted from published literature and are given fully referenced in the Supplementary Information. The CPT-L^48^ implementation of the simulation framework was done in ProbLog^47^ (https://dtai.cs.kuleuven.be/problog/problog1/problog1.html). Simulations were performed on Linux (48 cores) and MacBook Pro (6 cores) computers. SWI-Prolog^65^ served as the underlying programming environment (http://www.swi-prolog.org/). Analyses and plots were done with R (http://www.r-project.org). Furthermore, the network/model is publicly available at Github https://github.com/sysbio-bioinf/ProbRules.

## Supplementary information

Supplementary Information

## Data Availability

All data generated or analyzed during this study are included in this published article (and its Supplementary Information). Mass spectrometry proteomics data have been deposited to the ProteomeXchange Consortium via the PRIDE partner repository with the dataset identifier PXD011835.
