# Supplementary material for: Representing dynamic biological networks with multi-scale probabilistic models
Source: Commun Biol. 2019 Jan 17;2:21. doi: 10.1038/s42003-018-0268-3 (PMC6336720; doi:10.1038/s42003-018-0268-3)
Supplement: Supplementary file 1 — Supplementary Information [file 42003_2018_268_MOESM1_ESM.pdf]

Supplementary Information

Contents

1 Supplementary Figures **2**

2 Supplementary Note **9**

2.1 ProbRules: a novel rule-based probabilistic modeling approach for multi-scale systems 9

2.1.1 Introduction to ProbRules 9

2.1.2 Grounding on Causal Probabilistic Time Logic 13

2.1.3 Specifying ProbRules models 16

2.1.4 Runtimes of ProbRules models 17

2.1.5 Implementation of ProbRules in SWI-Prolog: `ProbRules.pl` 18

2.2 Network motifs interaction graphs and implementations 25

2.2.1 Simple regulation, positive and negative autoregulation 26

2.2.2 Symmetric bi-fan motif 28

2.2.3 Asymmetric bi-fan motif 30

2.2.4 Coherent feed-forward loop type I 32

2.2.5 Incoherent feed-forward loop type I 34

2.2.6 Single input module 36

2.2.7 Using immediate rates disables ProbRules models to reproduce network motifs dynamics 39

2.3 ProbRules model of Wnt/β-catenin and Wnt/JNK signaling 41

2.3.1 Logical relations in the Wnt signaling network 41

2.3.2 ProbRules source code for the Wnt signaling model 58

2.3.3 Wnt model feedbacks 73

2.3.4 Model dynamics and robustness 75

3 Supplementary Methods **81**

3.1 StealthRNA/siRNA sequences 81

3.2 RT-PCR primer sequences 81

4 Supplementary References **82**

1 Supplementary Figures

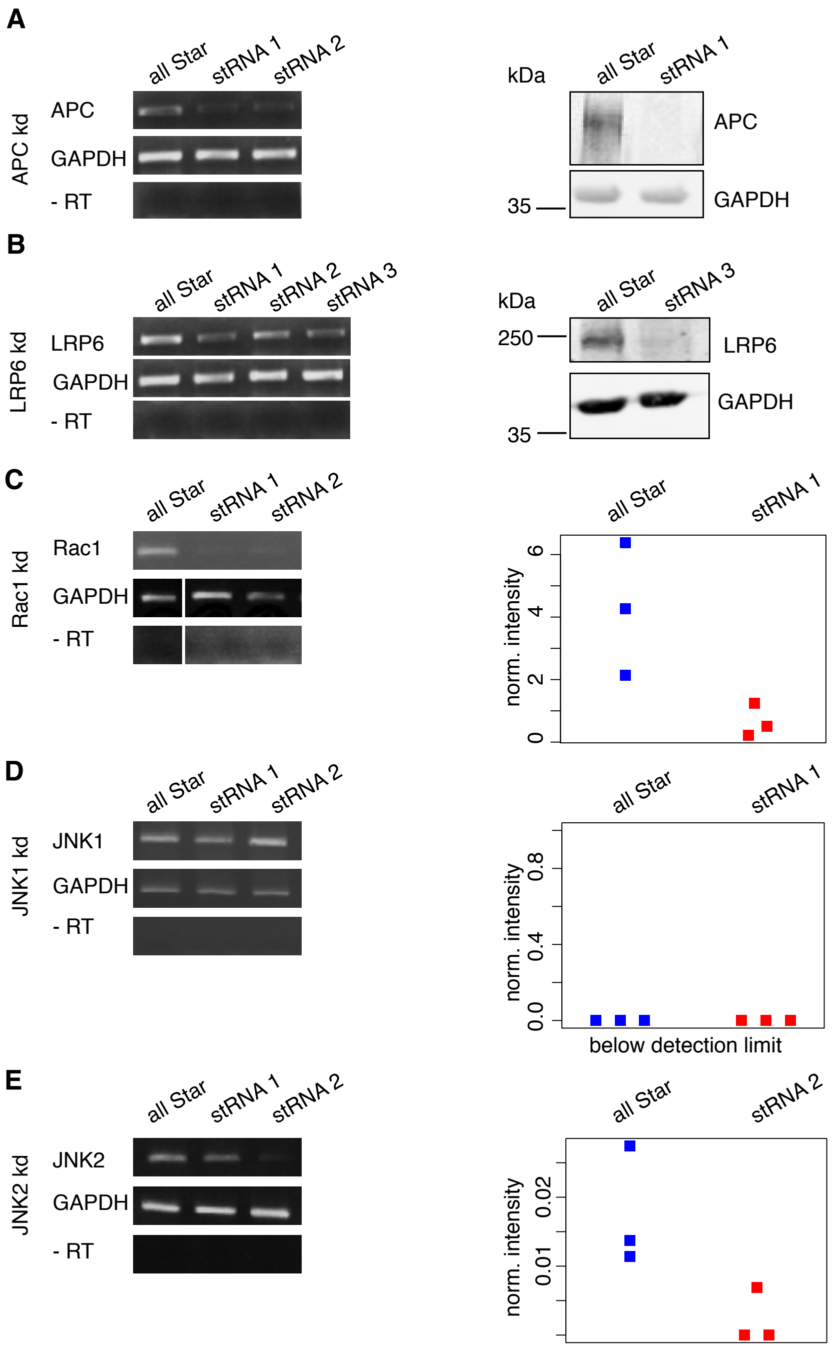

Supplementary Figure 1: Validation of knockdown (kd). The knockdown (kd) efficiencies of the various stealthRNAs (stRNA) of APC (adenomatous polyposis coli) (A), LRP6 (lipoprotein receptor related protein 6 (B), Rac1 (ras related C3 botulinum toxin substrate 1) (C), JNK1 (c-Jun N-terminal kinase 1) (D) and JNK2 (E) were validated on RNA level by PCR (left panels). To exclude possible contamination of the RNA with genomic DNA we performed cDNA synthesis without Reverse Transcriptase (-RT). In addition we validated the knockdown efficiency on protein level (right panels) by Western-Blot (A-B) as well as by mass spectrometry (C-D). GAPDH (glycerinaldheyd-3-phosphat-dehydrogenase) was used as loading control for PCRs and Western-Blots. In the case of JNK1, the protein amount of JNK1 was below the detection limit.

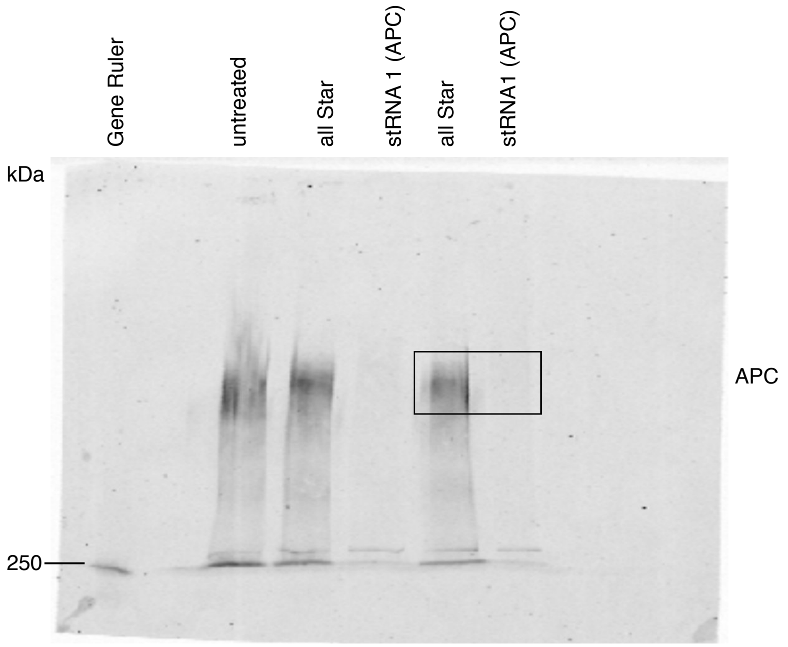

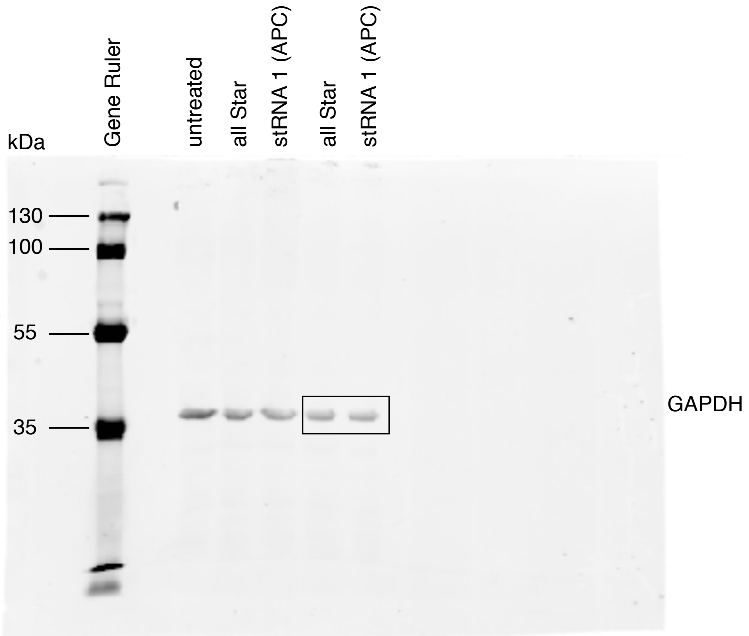

Supplementary Figure 2A: Uncropped images of the membranes of the APC (adenomatous polyposis coli) Western Blot for Supplementary Figure 1A. The image section shown in Supplementary Figure 1A is marked by a black box. GAPDH (glycerinaldehy-3-phosphat-dehydrogenase) was used as a house keeping gene. Note that samples were run on different geles due to the size of APC. The stealthRNA (stRNA) used is depicted above the images.

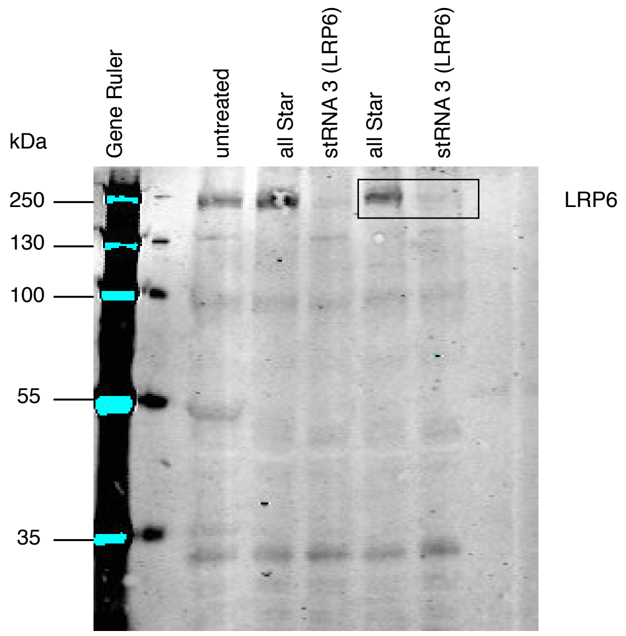

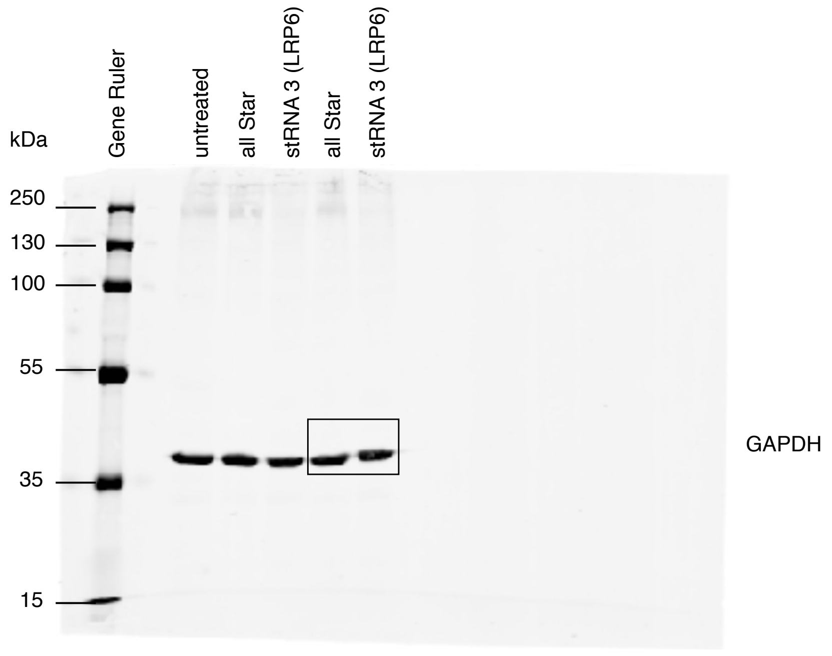

Supplementary Figure 2B: Uncropped images of the membrane of the LRP6 (lipoprotein receptor related protein 6) Western Blot for Supplementary Figure 1B. The image section shown in Supplementary Figure 1B is marked by a black box. GAPDH (glycerinaldehy-3-phosphat-dehydrogenase) was used as a house keeping gene. The stealthRNA (stRNA) used is depicted above the images.

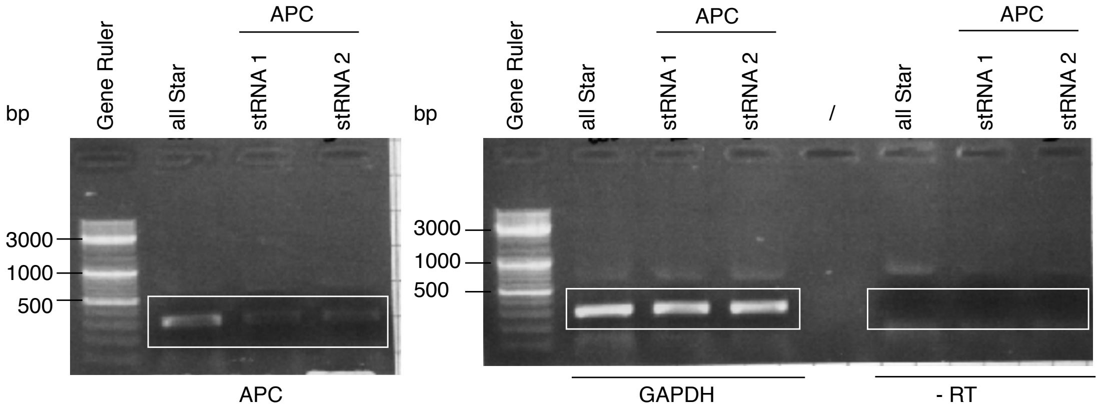

Supplementary Figure 2C: Uncropped images of agarose gels of APC (adenomatous polyposis coli) PCR (left). To exclude possible contamination of the RNA with genomic DNA we performed cDNA synthesis without Reverse Transcriptase (-RT). As control, we performed PCR with GAPDH (glycerinaldehy-3-phosphat-dehydrogenase) (right panel). The stealthRNAs (stRNA) used are depicted above the images. The image section shown in Supplementary Figure 1A is marked by white boxes.

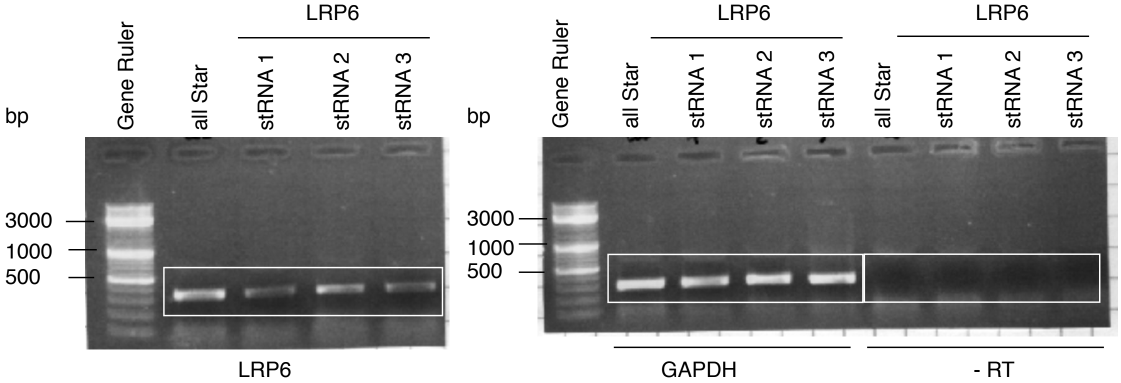

Supplementary Figure 2D: Uncropped images of agarose gels of LRP6 (lipoprotein receptor related protein 6) PCR (left). To exclude possible contamination of the RNA with genomic DNA we performed cDNA synthesis without Reverse Transcriptase (-RT). As control, we performed PCR with GAPDH (glycerinaldehy-3-phosphat-dehydrogenase) (right panel). The stealthRNAs (stRNA) used are depicted above the images. The image section shown in Supplementary Figure 1B is marked by white boxes.

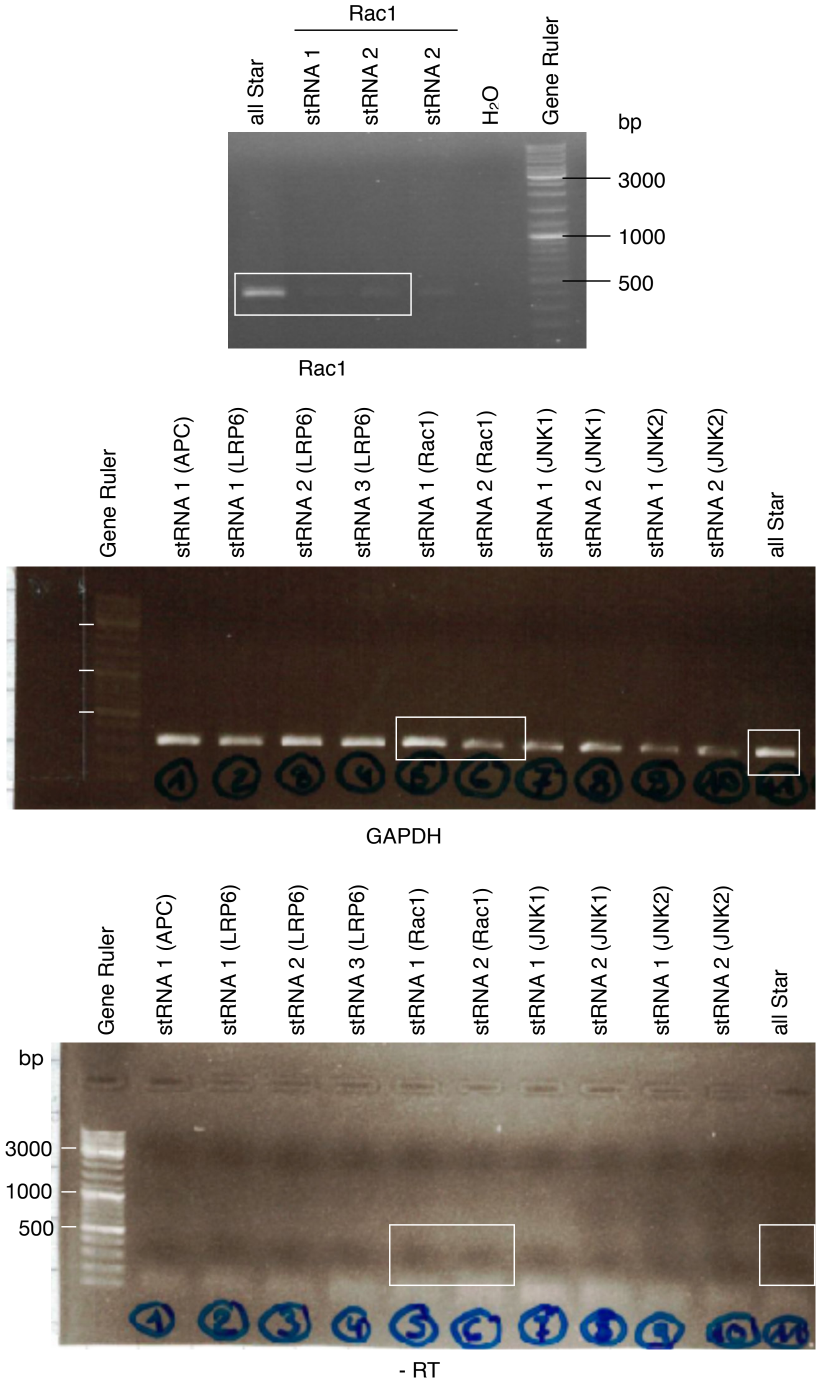

Supplementary Figure 2E: Uncropped images of agarose gels of Rac1 (ras-related C3 botulinum toxin substrate 1) PCR (upper gel). To exclude possible contamination of the RNA with genomic DNA we performed cDNA synthesis without Reverse Transcriptase (-RT) (lower image). As control, we performed PCR with GAPDH (glycerinaldehy-3-phosphat-dehydrogenase) (middle image). The stealthRNAs (stRNA) used are depicted above the images. The image section shown in Supplementary Figure 1C is marked by white boxes.

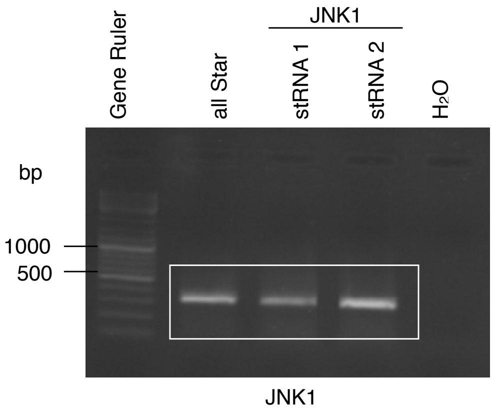

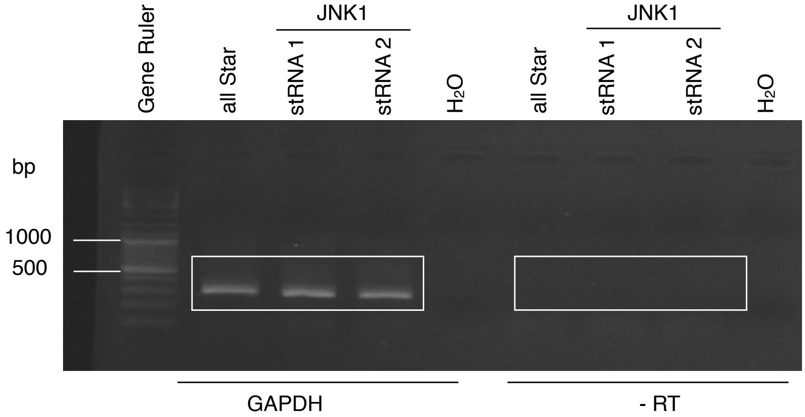

Supplementary Figure 2F: Uncropped images of agarose gels of JNK1 (c-Jun N-terminal kinase 1) PCR (upper image). To exclude possible contamination of the RNA with genomic DNA we performed cDNA synthesis without Reverse Transcriptase (-RT). As control, we performed PCR with GAPDH (glycerinaldehy-3-phosphat-dehydrogenase) (lower image). The stealthRNAs (stRNA) used are depicted above the images. The image section shown in Supplementary Figure 1D is marked by white boxes.

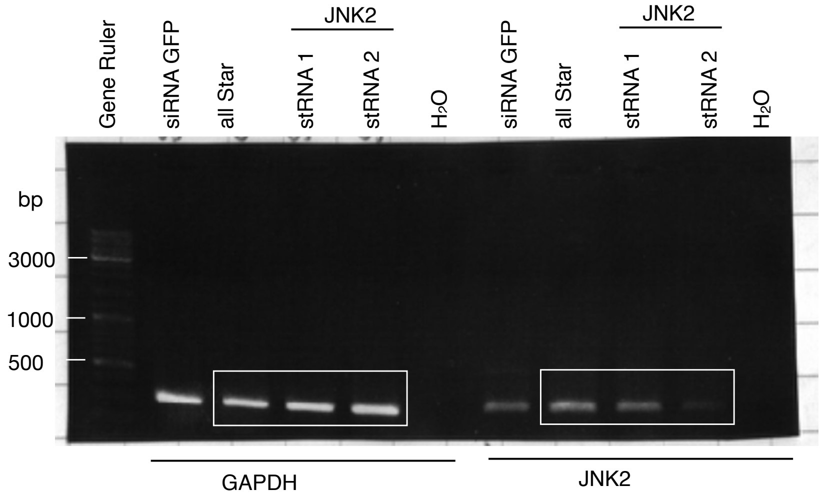

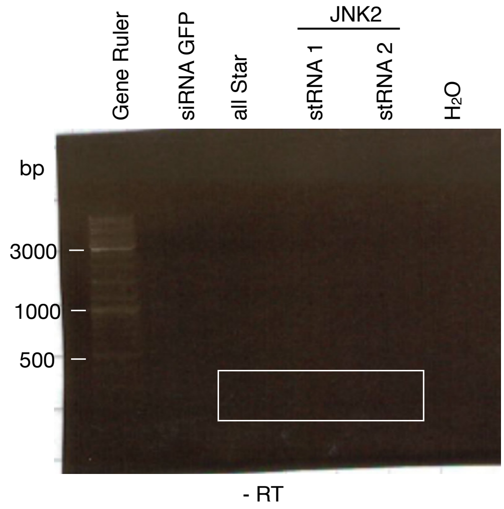

Supplementary Figure 2G: Uncropped images of agarose gels of JNK2 (c-Jun N-terminal kinase 2) PCR (upper image). To exclude possible contamination of the RNA with genomic DNA we performed cDNA synthesis without Reverse Transcriptase (-RT). As control, we performed PCR with GAPDH (glycerinaldehy-3-phosphat-dehydrogenase) (upper image). The stealthRNAs (stRNA) used are depicted above the images. The image section shown in Supplementary Figure 1E is marked by white boxes.

2 Supplementary Note

2.1 ProbRules: a novel rule-based probabilistic modeling approach for multi-scale systems

2.1.1 Introduction to ProbRules

A ProbRules model consists of a *graph* of interactions and a set of *rules*. The *vertices* of the graph represent components of a system. In models of biochemical systems, these can correspond to macromolecules like proteins, DNA and RNA or small molecule compounds like guanosine diphosphase (GDP). Possible interactions among these components are represented by *edges* in the graph. For a thorough introduction to graph theory see [1]. Besides direct interactions, such edges can also represent component states. For example, we used edges to represent protein modifications like phosphorylation in our study. Fig. N1 contains an example of a static interaction graph.

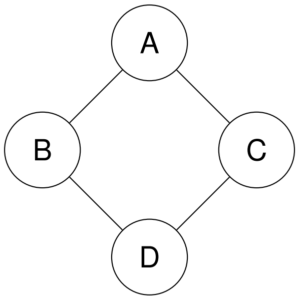

Figure N1: An example of a static interaction graph. Four components (A,B,C and D) and four interactions are specified. A interacts with B and C, and additionally D interacts with B and C.

To represent a *state* S_t at time t of the model, *probabilities* p_t are assigned to the interactions [2]. These probabilities represent *a measure of the presence* of the corresponding interactions. Accordingly, the presence of an interaction can range from 0, representing *complete absence*, to 1, representing *full presence*. These states S_t can be seen as random graph models in which each edge is sampled with the associated probability. Furthermore, as the probabilities of the interactions are assigned to distinct *time points* t, their sequences can represent *dynamic behavior*.

To represent the dynamics of the biochemical network in the model, *interdependencies* between the interactions are encoded by *rules*. Each rule affects a single *target interaction* driving its state towards a defined *target probability* by a specified *attack rate* and the *activity of the rule*. The *rule's activity* at time t is evaluated as the probability that its *Boolean formula* holds on state S_t using the rules of probability calculus. The interaction's state is also driven to its *initial* probability via a *global decay rate* when the rules for that interaction are not active. The example from Fig. N1 can be extended by interdependency rules as shown in Fig. N2. It includes two rules. In each of these rules a single source interaction triggers activation respectively inhibition of a target interaction. Now follows a more formal description of the ProbRules modeling approach.

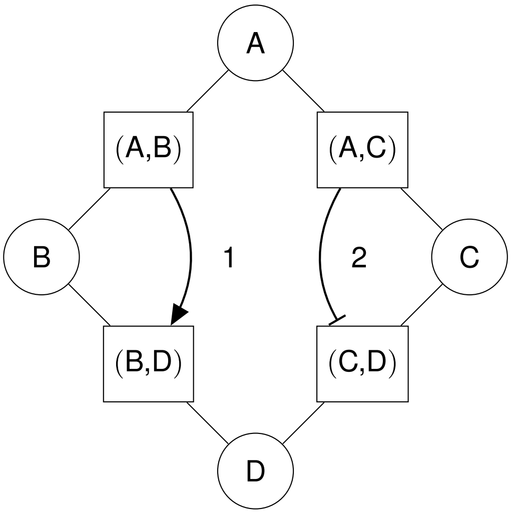

Figure N2: The example from Fig. N1 extended by interdependency rules between the interactions. Here, rule 1 lets interaction (A,B) activate (B,D) while rule 2 lets interaction (A,C) inhibit (C,D).

Static Interaction Graph

The graph of interactions $GI = (V, \mathcal{E})$ consists of a set V of vertices representing system components and a set $\mathcal{E} \subseteq V \times V$ of edges representing possible interactions of the components. As such, the graph represents the static structure of the modeled system. The example shown in Fig. N1 is defined by $V = \{$A,B,C,D$\}$ and $\mathcal{E} = \{$(A,B), (A,C), (B,D), (C,D)$\}$.

Model states, time points and dynamics

The state of an interaction $(i, j) \in \mathcal{E}$ of two components $i \in V$ and $j \in V$ at a time point t is denoted by the probability $p_t(i, j)$. A state $S_t(\mathcal{E})$ of the model for a time point t is defined by corresponding probabilities p_t attached to the edges $\mathcal{E}$ of the graph GI:

$$S_t(\mathcal{E}) = \{ \ p_t(i,j) \in [0,1] \quad | \quad (i,j) \in \mathcal{E} \ \}$$

Each such S_t defines a random graph model which essentially is a probability distribution $\mathcal{D}_t$ over possible subgraphs $G = (V, \mathcal{E}_G)$ of GI with $\mathcal{E}_G \subseteq \mathcal{E}$ [3]. Therefore the probability $Pr(G)$ of a subgraph G is

$$Pr(G) = \prod_{e \in \mathcal{E}_G} p_t(e) \prod_{e \in \mathcal{E} \setminus \mathcal{E}_G} (1 - p_t(e))$$

The edges (i, j) can be viewed as independent random variables that are true with probability $p_t(i, j)$.

Dynamics of a modeled system can be represented by a sequence of states $S_0, S_1, ..., S_T$. Thereby, the probabilities of the different interactions can evolve over time. Although the random variables corresponding to the edges are independent from each other at any particular point in time t, interdependencies between the interactions at different time points can be introduced. This is achieved in a controlled way by evaluating rules.

Interdependency Rules

A set $\mathcal{R}$ of rules defines the interdependencies between activity states of interactions. Each rule takes the form

$$r \quad : \quad \phi \quad \Longrightarrow \quad p(i,j) \quad \xrightarrow{a_r} \quad q$$

where ϕ represents a Boolean condition (*formula*) on *source* interaction states and (i,j) is the affected target interaction whose activity state $p(i,j)$ is driven towards the target probability q by the attack rate a. Interdependencies between interactions can act by driving target interactions' probabilities towards arbitrary values using arbitrary attack rates. The generality of this formulation enables a straightforward translation of biochemical experimental findings into a ProbRules model as this was experienced during the development of the Wnt signaling pathway model. For the present work it was sufficient to use a single conjunction (Boolean AND) of the states of several, possibly negated source interactions in the formula ϕ.

For the extended example in Fig. N2 two rules can be specified:

$$\begin{array}{lllllll} 1 & : & p(\text{A,B}) & \Longrightarrow & p(\text{B,D}) & \xrightarrow{a_1} & 1 \\ 2 & : & p(\text{A,C}) & \Longrightarrow & p(\text{C,D}) & \xrightarrow{a_2} & 0 \end{array}$$

Given a model state S_t at time t, the probability of activation ϕ_t of a rule's formula ϕ can be determined using the rules of probability calculus. Thus, ϕ_t denotes the probability that the logical formula ϕ holds in a randomly sampled subgraph according to the distribution $\mathcal{D}_t$.

The interdependency rules operate in a step-wise manner in regard to the evolution in time. At each point in time $t-1$, each rule r proposes a new probability value $q_t(r,i,j)$ for its target interaction (i,j). First, a discussion of the effects of rules independently of one another follows and then a description how the effects of different rules are combined.

Interaction states proposed by rules

A basic rule r with condition ϕ, target (i,j), target probability q, and attack rate a has to be read as: whenever ϕ holds at time $t-1$ the probability of the interaction (i,j) at time t will be

$$q_t(r,i,j) \quad = \quad (1-a)\cdot p_{t-1}(i,j) + a \cdot q$$

That means that whenever $\phi_{t-1} = 1$ the new probability for (i,j) is an affine combination of the previous state $p_{t-1}(i,j)$ and the target probability q as determined by the attack rate a.

In general (for arbitrary S_t) the condition ϕ will not hold on all subgraphs that can be sampled according to $\mathcal{D}_t$, and thus the probability ϕ_t will be < 1. To account for this, the rules contribute only with a corresponding factor $\phi_{t-1} \cdot q_t(r,i,j)$ to the target $p_t(i,j)$. Due to subgraphs where ϕ_t is false, a factor for the negation $(1-\phi_{t-1})$ would be lost at each time step. To account for this lost factor and subgraphs, a standard decay rule is introduced.

Decay

The standard decay rule states that an interaction that is not affected by any active rule returns to its defined initial state $p_0(i,j)$ by a global decay rate d:

$$q_t(d,i,j) \quad = \quad (1-d)\cdot p_{t-1}(i,j) + d\cdot p_0(i,j)$$

Combining one rule with the decay rule

Combining a single effective rule with the default decay rule yields the value

$$p_t(i,j) = \phi_{t-1}\cdot q_t(r,i,j) + (1-\phi_{t-1})\cdot q_t(d,i,j) \tag{1}$$

Thus the decay rule applies only when the other rule is not active.

Combining an arbitrary number of rules

Consider now rules $r_1,...,r_n$ with formulas $\phi_1,...,\phi_n$ which target the same interaction (i,j). They each propose new states $q_t(r_1,i,j),...,q_t(r_n,i,j)$ of that interaction. A method for combination can be obtained by considering the meaning of a rule r_k. Such a rule basically states that whenever ϕ_k holds - for those subgraphs for which ϕ_k holds - the target has to be set to $q_t(r_k,i,j)$. A concrete realization consists of subgraphs for which exactly m conditions $\phi_{k1},...,\phi_{km}$ hold with corresponding $q_t(r_{k1},i,j),...,q_t(r_{km},i,j)$. In these cases, the combination will be the average to these subgraphs:

$$q_t(\Phi_{\{k1,...,km\}},i,j) \quad = \quad \frac{1}{m}\sum_{i=1}^{m} q_t(r_{ki},i,j) \tag{2}$$

where

$$\Phi_{\{k1,...,km\}} = \bigwedge_{s\in\{k1,...,km\}}\phi_s \bigwedge_{s\in\{1,...,n\}-\{k1,...,km\}}\neg\phi_s \tag{3}$$

As for the single rule case, the decay rule still applies for subgraphs in which $\bigvee_{i=1}^{n}\phi_i$ is false.

Synchronous update

After deriving the proposed next states p_t of all interactions (i,j) based on the previous interaction state S_{t-1} yields the new interaction state S_t. Then, a new cycle can be started which yields new interaction states and so on, until some final time point T is reached. This allows to simulate dynamics of biochemical systems like the Wnt signaling networks using their static interaction graph and interdependency rules on the states of the interactions.

Inputs and perturbations

Inputs can be provided to a ProbRules model at specific interactions and times by specifying an explicit probability

$$p_t(i,j) = \texttt{fixed}(i,j,t)$$

This also allows to investigate perturbations like inhibition and constitutive activation of a specific interaction (i,j) in a specified ProbRules model.

2.1.2 Grounding on Causal Probabilistic Time Logic

The introduced ProbRules model is based on notions from probabilistic logic programming [4] and statistical relational learning [5]. In particular, it is inspired on the CPT-L framework which forms a logical foundation. This section describes how the ProbRules interdependency rules (like CPT-L) can be translated into ProbLog [3], a recently introduced probabilistic extension of the programming language Prolog [6].

Causal Probabilistic Time Logic (CPT-L)

The ProbRules interdependency rules are a variation on the rules used in the CPT-L formalism of [7]. [1] CPT-L rules are of the form

$$r \quad = \quad (h_1 : p_1) \quad \vee \quad ... \quad \vee \quad (h_n : p_n) \quad \leftarrow \quad \phi$$

where the h_i are propositional (Boolean) variables, ϕ is a Boolean expression over these variables, and $p_i \in [0,1]$ probabilities with $\sum_{i=1}^{n} p_i = 1$. The semantics of a CPT-L rule is that whenever the expression ϕ is true at time $t-1$, one of the h_i will be sampled with probability p_i and will become true at time t. As we are working in a probabilistic logic the expression ϕ will only be true with a particular probability p_ϕ. This probability will then be taken into account in the sampling process: the h_i's will be sampled with probability $p_\phi \cdot p_i$.

A CPT-L theory is a set of CPT-L rules. It may have multiple rules targeting the same propositional variables. These rules will be combined as two independent alternative causes to generate these targets. This corresponds to a disjunctive interpretation of the rules.

Stochastic Relational Process

A CPT-L theory specifies a probability distribution over sequences of logical interpretations. An interpretation is an assignment of truth-values to all propositional variables in the theory. So, a CPT-L theory defines a probability distribution over sequences of such interpretations $I_1, ..., I_t$ which is a stochastic relational process.

Semantics of interdependency rules

The ProbRules interdependency rules defined before are essentially variants of CPT-L rules. Summarizing on this:

- The propositions represent the presence or absence of an edge in the interaction graph. This implies that an interpretation corresponds to a subgraph of the interaction graph.
- The target probabilities of a CPT-L theory are immediate, whereas those of the ProbRules interdependency rules may involve more than one time step. Nevertheless, in the previous section was shown how the immediate probabilities can be computed.

[1] We do make some simplifications in the current presentation as we only need the propositional subset of CPT-L and not the first order logic one.

- Each ProbRules interdependency rule has only a single target, whereas a CPT-L theory may have multiple targets (each h_i is a target).
- Different ProbRules interdependency rules are combined using *averaging* whereas CPT-L rules are combined using *OR*.

Implementing ProbRules interdependency rules

Among the ProbRules interdependency rules that can easily be mapped onto a set of CPT-L rules are the cases with only a single rule for a target (i, j):

$$\phi \implies p(i,j) \xrightarrow{a} q$$

which can be represented in CPT-L by

$$\begin{aligned} p(i,j) &\leftarrow \phi \wedge \neg a \wedge p(i,j) \\ p(i,j) &\leftarrow \phi \wedge a \wedge q \\ p(i,j) &\leftarrow \neg\phi \wedge \neg d \wedge p(i,j) \\ p(i,j) &\leftarrow \neg\phi \wedge d \wedge p_0(i,j) \end{aligned}$$

Here each CPT-L rule has only one possible conclusion ($p(i, j)$). The probabilistic constants a, d and q are represented as Boolean variables that are true with their corresponding probability.

Evaluating the probability $p(i, j)$ using these CPT-L rules corresponds to computing the probability of the logical formula:

$$\begin{aligned} p_t(i,j) &= Prob\{(\phi \wedge \neg a \wedge p(i,j)) \bigvee (\phi \wedge a \wedge q) \\ &\quad \bigvee (\neg\phi \wedge \neg d \wedge p(i,j)) \bigvee (\neg\phi \wedge d \wedge p_0(i,j))\} \\ &= Prob\{\phi \wedge \neg a \wedge p(i,j)\} + Prob\{\phi \wedge a \wedge q\} + \\ &\quad Prob\{\neg\phi \wedge \neg d \wedge p(i,j)\} + Prob\{\neg\phi \wedge d \wedge p_0(i,j)\} \\ &= \phi_{t-1} \cdot (1-a) \cdot p_{t-1}(i,j) + \phi_{t-1} \cdot a \cdot p_{t-1}(i,j) + \\ &\quad (1-\phi_{t-1}) \cdot (1-d) \cdot p_{t-1}(i,j) + (1-\phi_{t-1}) \cdot d \cdot p_0(i,j) \end{aligned}$$

Since the four terms are disjoint, the probability of the disjunctive expression is the sum of the probabilities of the expressions. The resulting expression has the same value as in Equation 1.

A set of n ProbRules interdependency rules for the same target (i, j)

$$\begin{array}{ccccc} \phi_1 & \implies & p(i,j) & \xrightarrow{a_1} & q_1 \\ \vdots & \implies & p(i,j) & \vdots & \vdots \\ \phi_n & \implies & p(i,j) & \xrightarrow{a_n} & q_n \end{array}$$

can be represented in a similar way in CPT-L. The two rules for decay are retained yielding as before:

$$\begin{aligned} p(i,j) &\leftarrow \neg\Phi \wedge \neg d \wedge p(i,j) \\ p(i,j) &\leftarrow \neg\Phi \wedge d \wedge p_0(i,j) \end{aligned}$$

where $\Phi = \vee_i \phi_i$. The attack rules are now of the following form:

$$\begin{aligned} p(i,j) &\leftarrow \Phi_{\{k1,\dots,km\}} \wedge \neg a_{ki} \wedge p(i,j) \wedge m_{ki} \\ p(i,j) &\leftarrow \Phi_{\{k1,\dots,km\}} \wedge \ a_{ki} \wedge \ q_{ki} \ \wedge m_{ki} \end{aligned}$$

where the $\Phi_{\{k1,\dots,km\}}$ are as defined in Equation 3. There is a pair of such rules for each possible ki in any possible subset $\{k1,...,km\} \subseteq \{1,...,n\}$. The m_{ki} are defined using the following rule:

$$(m_{k1} : \frac{1}{m}) \vee ... \vee (m_{km} : \frac{1}{m}) \leftarrow$$

which assures that the m_{ki} are mutually exclusive and each of them has the correct probability $1/m$. For any fixed $\{k1,...,km\}$ exist $2m$ rules for $p(i,j)$. Each pair of rules will contribute $\frac{1}{m}q_t(r_{ki},i,j)$ to $p_t(i,j)$ when $\Phi_{\{k1,\dots,km\}}$ holds. This yields the same result as in Equation 2. Because the formulas $\Phi_{\{k1,\dots,km\}}$ are mutually exclusive, the $q_t(\Phi_{\{k1,\dots,km\}},i,j)$ can simply be summed to account for the attacking rules. This is combined with the decay rule.

This basically shows that a set of ProbRules interdependency rules inherits its function from CPT-L. It defines a probability distribution over finite sequences of subgraphs. As already indicated, particular interest is in the sequence of states $(S_t(\mathcal{E}))_{t\geq 0}$ that is produced using a ProbRules model $(GI = (V,\mathcal{E}),\mathcal{R})$, its rule set $\mathcal{R}$, its interaction graph $GI = (V,\mathcal{E})$ and given an initial state S_0. Using CPT-L, each $S_t = \{p_t(i,j)|(i,j) \in \mathcal{E}\}$ is represented as a set of probabilistic propositions. The next state is then determined by computing the probability of (i,j) at time $t+1$ using the above CPT-L rules. In this way, the sequence of states S_t imposes a *stochastic graph process* over *discrete time* points.

Implementation in Prolog

Effective techniques for probabilistic inference have been developed and integrated in the programming language Prolog [6, 3]. The ProbRules implementation requires SWI-Prolog, which is available for all major operating systems. Alternatively, one can also use the SWI-Prolog Docker container available from Docker Hub. Implementations of several network motifs and the Wnt model are available at Github.

Further details are available in the README file there. See also SWI-Prolog Quickstart for details on using SWI-Prolog.

2.1.3 Specifying ProbRules models

ProbRules models are specified based on Prolog. For a more elaborate introduction to Prolog see [8, 6].

How to use the ProbRules implementation for model simulation

In order to use the ProbRules implementation for the simulation of a model of interest, we need to define involved interactions, interdependency rules between these and parameters which are used. At the start, we load the ProbRules implementation from `ProbRules.pl`:

```
:- consult( 'ProbRules.pl').
```

Parameters for target probabilities and attack rates are defined directly. The ProbRules implementation uses the `global_decay` parameter, and therefore it should be defined using that name. Other parameter can be defined as required for the rules:

```
1.0  :: on.
0.0  :: off.
0.1  :: global_decay.

0.1 :: sr_rate.
0.3 :: nar_rate.
0.03:: par_rate.
```

Interactions consist of *ordered* pairs of components. The attached probability is used for initialization and as the target value for decay:

```
0.0 :: interaction(s,r).
0.0 :: interaction(n,ar).
0.0 :: interaction(p,ar).
```

Inputs by interactions can be defined using the `fixed` predicate. We will use this in the Wnt model. Thus, `fixed` can be used for perturbations like knockout and overexpression of compounds, inhibition and constitutive activation of interactions in ProbRules models of biological systems.

In this example, we define a single simple regulation rule for the target interaction using no source, defined attack and target probability facts and a label which is required for distinguishing the rules:

```
rule((s,r),[],sr_rate,on,'sr activation').
```

Empty brakets `[]` enable a constantly active rule.

For *autoregulation* motifs we provide both activation and autoregulation rules:

```
rule((n,ar),[],nar_rate,on,'nar activation').
rule((n,ar),[not (n,ar)],nar_rate,on,'nar autoregulation').

rule((p,ar),[],par_rate,on,'positive autoregulation activation').
rule((p,ar),[(p,ar)],par_rate,on,'positive autoregulation').
```

For executing the simulation up to the time point 100, we use `evaluation`:

```
:- evaluation(100).
:- halt.
```

`halt` ends the Prolog interpreter and thus allows analyses to be scripted. Omitting `halt` opens an interactive environment that can be used for investigation of the model.

The numbered lines can be stored in a file, e.g. `srnarpar.pl`. See also the interaction graphs in the next chapter. Running without any preambles or frills is achieved by typing `swipl srnarpar.pl` at a shell console or terminal.

2.1.4 Runtimes of ProbRules models

model	#interactions	#rules	time [s]
srnarpar	3	5	0.168
bifana	9	8	0.245
bifanb	9	8	0.248
cffl1	4	3	0.369
iffl1	4	3	0.171
sim	8	7	0.014
WntModel	69	93	37.561

Timings were obtained on a recent MacBook Pro (Model 2018, i9, 2.9 GHz).

2.1.5 Implementation of ProbRules in SWI-Prolog: `ProbRules.pl`

```
:- op(50, xfx, ::).
:- op(70, fx, not).

:- discontiguous (::)/2.
:- multifile rule/5.
:- multifile fixed/3.

%!  evaluation(+Timepoint:int) is det.
%
%   The entry point to this application.
%   Computes and prints the state of the probabilistic interactions at
%   each timepoint from 0 to Timepoint.
evaluation(S0) :-
    succ(S0, S),
    initialize_variables(Assoc),
    init_visualize(Assoc),
    visualize(0, Assoc),
    evaluation(1, S, Assoc).

evaluation(S, S, _) :- !.
evaluation(A, S, Assoc) :-
    newvalues(A, Assoc, New_Assoc),
    visualize(A, New_Assoc),
    succ(A, A1),
    evaluation(A1, S, New_Assoc).

%!  initialize_variables(-Assoc) is det.
%
%   Initialize Assoc with the probabilistic terms in the knowledge
%   base. Atoms are keys, probabilities are values.
initialize_variables(Assoc) :-
    empty_assoc(Assoc0),
    setof(X-P, P::X, Interactions),
    update_facts_assoc(Interactions, Assoc0, Assoc).

%!  init_visualize(+Assoc) is det.
%
%   Print a single tab-separated line with the probabilistic
%   interaction identifiers that serve as keys in Assoc (sorted asc).
init_visualize(Assoc) :-
    assoc_to_keys(Assoc, Xs),
    findall((A, B), member(interaction(A, B), Xs), Interactions),
    forall(member(Interaction, Interactions),
       format('\t(~w)', [Interaction])),
    format('~n', []).

%!  visualize(+Iteration, +Assoc:association_list) is det.
%
%   Print a tab-separated line of interaction probabilities
%   (sorted asc). An iteration number is prepended to the line.
visualize(Iteration, Assoc) :-
    assoc_to_list(Assoc, Xs),
    format('~w\t', [Iteration]),
    forall(member(interaction(_,_)-X, Xs), format('~w\t', [X])),
    format('~n', []).

%!  newvalues(T, Assoc, New_Assoc) is det.
```

```
%
%   Generate values for the next time point.
%   For each interaction (i.e. key) in Assoc do:
%   - check which rules are applicable and compute their target scores.
%   - take the average and associate it as the new value.
newvalues(T, Assoc, New_Assoc) :-
    assoc_to_keys(Assoc, Probabilistic_Atoms),
    findall(interaction(A,B),
        member(interaction(A,B), Probabilistic_Atoms),
        Interactions),
    newvalues_helper(T, Interactions, Assoc, New_Interactions),
    update_facts_assoc(New_Interactions, Assoc, New_Assoc).

%!  average(+Xs:number_list, -Y:number) is det.
%
%   Y is the average of the values in Xs.
average(Xs, Y) :-
    sum_list(Xs, T),
    length(Xs, L),
    Y is T / L.

%!  newvalues_helper(+Timepoint:int,
%!                   +Interactions,
%!                   +Assoc:association_list,
%!                   +Interaction_Probabilities) is det.
%
%   For each interaction in Interactions its probability for timepoint
%   Timepoint is computed and stored in Interaction_Probabilities.
%
%   The probability of an interaction is computed as follows:
%   - If a fixed probability for Interaction at timepoint Timepoint is
%     known, this becomes its probability.
%   - Otherwise, collect all rules potentially affecting Interaction
%     and compute the probability using interaction_probability/4.
newvalues_helper(_, [], _, []) :- !. % Ideally SWI Prolog would be
                     % able to identify that the empty
                     % list is mutually exclusive with
                     % the other clause for these
                     % arguments, however this is
                     % currently not the case, hence
                     % the cut to avoid a useless
                     % choice point.
newvalues_helper(A, [Interaction | Xs], Assoc, [Interaction-P | Ys]) :-
    ( static(A, Assoc, Interaction, P), !
    ; findall(Conditions-(Attack_Rate, Target_Probability),
          dynamics(Interaction, Conditions, Target_Probability, Attack_Rate),
          Rules),
      interaction_probability(Interaction, Assoc, Rules, P)
    ),
    newvalues_helper(A, Xs, Assoc, Ys).

%!  static(+Timepoint:int,
%!         +Assoc:association_list,
%!         +Interaction,
%!         -P:probability) is semidet.
%
%   Returns the probability P of a given Interaction at timepoint
%   Timepoint in case a fixed/3 statement is applicable.
%   Fails if not.
```

```
static(Timepoint, Assoc, interaction(X, Y), P) :-
    clause(fixed(X, Y, Timepoint), Body),
    conj_to_list(Body, Body_Literals),
    process(Assoc, Body_Literals, Ps),
    product(Ps, P).

%!  conj_to_list(Xs:conjunction, Ys:list) is det.
%
%   Ys is the order-preserved list of terms that made up Xs.
conj_to_list(’,’(H, Conj), [H | T]) :-
    !,
    conj_to_list(Conj, T).
conj_to_list(H, [H]).

%!  process(+Assoc:association_list,
%!          +Xs:list,
%!          -Ys:probability_list) is semidet.
%
%   Ys are the probabilities associated with their respective
%   terms in Xs.
%   Xs can contain probabilistic as well as nonprobabilistic terms
%   (assumed to be det). The deterministic terms are called using call/1.
%   The probability associated with a deterministic term is the
%   neutral element for multiplication 1.
process(_, [], []).
process(Assoc, [X|Xs], [P|Ps]) :-
    process_literal(Assoc, X, P),
    process(Assoc, Xs, Ps).

process_literal(Assoc, X, P) :-
    ( get_assoc(X, Assoc, P), !
    ; call(X), P = 1
    ).

dynamics(interaction(X, Y), Conditions, Target_Probability, Attack_Rate) :-
    rule((X, Y), Conditions, Target_Probability, Attack_Rate, _Description).

%!  evaluate_conditions(+Conditions:list,
%!                      +Assoc:association_list,
%!                      -P:probability) is semidet.
%
%   P is the probability that the probabilistic variables in
%   Conditions hold.
%
%   Conditions contains positive or negated independent probabilistic facts.
%   Assoc has interactions (interaction/2) as keys and probabilities
%   as values.
%   If Conditions = [] then P = 1.
%   If length(Conditions, L), L > 0 then a probability is derived from
%   a condition by taking its probability from
%   Assoc and taking the complement in case it is negated.
%   P then equals the product of all probabilities.
evaluate_conditions(Conditions, Assoc, P) :-
    maplist(condition_probability(Assoc), Conditions, Probabilities),
    product(Probabilities, P).

%!  condition_probability(+Assoc:association_list,
%!                        +Condition,
%!                        -P:probability) is semidet.
```

```
%
%   If Condition is positive, then P is the probability specified by
%   the interaction/2 associated with Condition
%   in Assoc.
%   If Condition is negated, then P is the complement of that probability.
condition_probability(Assoc, not (X, Y), P) :-
    get_assoc(interaction(X, Y), Assoc, P0),
    P is 1 - P0.
condition_probability(Assoc, (X, Y), P) :-
    get_assoc(interaction(X, Y), Assoc, P).

%!  update_facts_assoc(+Probabilistic_Facts:pairs,
%!                           +Assoc_In:association_list,
%!                           -Assoc_Out:association_list) is det.
%
%   Assoc_Out is Assoc_In extended with each pair (serving as
%   (Key,Value)) of Probabilistic_Facts.
update_facts_assoc([], Assoc, Assoc).
update_facts_assoc([Fact-P | Xs], Assoc0, Assoc) :-
    put_assoc(Fact, Assoc0, P, Assoc1),
    update_facts_assoc(Xs, Assoc1, Assoc).

%!  product(+Xs:numberlist, -P:number) is det.
%
%   True if P is the product of the elements in Xs.
product(Xs, P) :-
    product_helper(Xs, 1, P).

product_helper([], Acc, P) :-
    P is Acc.
product_helper([X | Xs], Acc, P) :-
    product_helper(Xs, X * Acc, P).

%!  interaction_probability(+Interaction,
%!                          +Assoc:association_list,
%!                          +Xs,
%!                          -P:probability) is semidet.
%
%   P is the probability of Interaction by taking into account
%   the potential effects of the rules in Xs on its state in the
%   previous time point as stored in Assoc.
%
%   Format of Xs = [[(i,p)]-(global_attack,on),
%                  [(x,z), not (y,z)]-(global_attack, on)]
interaction_probability(Interaction, Assoc, Xs, P) :-
    findall(P,
        (annotated_rules(Xs, World),
         evaluate_world(Assoc, Interaction, World, P)),
        Ps),
    sum_list(Ps, P).

%!  evaluate_world(+Assoc:association_list,
%!                 +Interaction,
%!                 +Xs,
%!                 -Probability) is semidet.
%
%   Xs is a set of rules associated with a particular Interaction
%   evaluated against a particular World. The World in question
%   is one world consisting of the variables used in the conditions of
```

```
%   that set of rules. Status indicates whether a particular Rule is
%   active in the World.
%
%   - If all the rules are evaluated as false in a particular world,
%     then the decay rule applies. The Weight of the world is used for
%     the Condition probability in the decay rule formula, except when
%     Xs = [], since then the rules affecting the Interaction have no
%     conditions (i.e. are always satisfied), in which case the Condition
%     probability is 1.
%   - If some rule is evaluated as true, then the probability of
%     each true rule is evaluated separately using the effective
%     rule formula, and then resulting probability are averaged out.
%
%   Note: format of Xs: for X in Xs:
%   X = World-Condition-(Attack_Rate, Target_Probability)-Status
%   where
%   - World = one World (truth table generated) over the variables
%             used in the conditions of the rules affecting Interaction.
%   - Condition = the Condition that triggers a particular rule
%                 on Interaction.
%   - Attack_Rate = The attack rate of that some rule.
%   - Target_Probability = The target probability of that same rule.
%   - Status = Whether Condition actually holds in World.
evaluate_world(Assoc, Interaction, Xs, Probability) :-
    ( maplist(status(false), Xs) ->
      get_assoc(global_decay, Assoc, Global_Decay),
      get_assoc(Interaction, Assoc, Previous_P),
      Initial_P :: Interaction,
      ( memberchk(World-_-_-_, Xs) ->
        evaluate_conditions(World, Assoc, Condition)
      ;
        Condition = 1
      ),
      default_decay_rule_formula(Condition, Global_Decay, Initial_P, Previous_P, Probability)
    ;
          include(status(true), Xs, Ys),
      maplist(evaluate(Assoc, Interaction), Ys, Ps),
      average(Ps, Probability)
    ).

%!  evaluate(+Assoc,
%!           +Interaction,
%!           +World_Rule,
%!           -P)  is semidet.
%
%   World_Rule = World-_-(Target_Probability_Atom,Attack_Rate_Atom)-_
%
%   P is the probability as specified by the formal effective rule
%   formula for Interaction where
%   - the condition probability is the probability of World.
%   - the Target Probability and Attack Rate is explicitly provided
%     through atoms that can be used as keys in Assoc.
%   - Interaction is used as a key in Assoc to retrieve its
%     probability at the previous time point.
evaluate(Assoc, Interaction, World-_-(Target_Probability_Atom, Attack_Rate_Atom)-_, P) :-
    evaluate_conditions(World, Assoc, Condition_P),
    get_assoc(Target_Probability_Atom, Assoc, Target_Probability),
    get_assoc(Attack_Rate_Atom, Assoc, Attack_Rate),
    get_assoc(Interaction, Assoc, Previous_P),
```

```
    effective_rule_formula(Condition_P, Target_Probability, Attack_Rate, Previous_P, P).

%!  default_decay_rule_formula(+Condition:probability,
%!                             +Global_Decay:probability,
%!                             +Initial_P:probability,
%!                             +Previous_P:probability,
%!                             -P:probability) is det.
%
%   P is the probability as specified by the formal default decay rule formula.
default_decay_rule_formula(Condition, Global_Decay, Initial_P, Previous_P, P) :-
    P is Condition * ((1 - Global_Decay) * Previous_P + Global_Decay * Initial_P).

%!  effective_rule_formula(+Condition:probability,
%!                         +Target_Probability:probability,
%!                         +Attack_Rate:probability,
%!                         +Previous_P:probability,
%!                         -P:probability) is det.
%
%   P is the probability as specified by the formal effective rule formula.
effective_rule_formula(Condition, Target_Probability, Attack_Rate, Previous_P, P) :-
    P is Condition * ((Target_Probability * Attack_Rate) + (1 - Attack_Rate ) * Previous_P).

status(State, _-_-_-State).

%!  annotated_rules(+Rules, -World_Rules_Satisfied:hyphen_quadruple_list) is multi
%
%    World_Rules_Satisfied is a list of quadruples.
%    Each binding of World_Rules_Satisfied is a list of Rules
%    evaluated against a particular World. Upon backtracking a list for
%    each possible world is returned.
annotated_rules(Rules, World_Rules_Satisfied) :-
    condition_TA_Pairs_condition_set(Rules, Condition_Variables),
    random_variables_world(Condition_Variables, World),
    maplist(condition_satisfaction_in_world(World), Rules, World_Rules_Satisfied).

%!  condition_satisfaction_in_world(+World,
%!                                  +Condition_L:hyphen_pair,
%!                                  -World_Conditions_L_State:hyphen_quadruple)
%!                                  is det.
%
%   State is true if Conditions are satisfied by World.
%   That is, if each element in Conditions is a member of World.
%   State is false otherwise.
condition_satisfaction_in_world(World, Conditions-L, World-Conditions-L-State) :-
    ( forall(member(Condition, Conditions), member(Condition, World))
    -> State = true
    ;  State = false
    ).

%!  condition_TA_Pairs_condition_set(+Condition_TA_Pairs:pairs,
%!                                   -Condition_Set:set) is det.
%
%   Condition_Set is the set of interactions contained in the
%   keys (a key is a list of positive or negated interactions) of
%  Condition_TA_Pairs.
condition_TA_Pairs_condition_set(Condition_TA_Pairs, Condition_Set) :-
    pairs_keys(Condition_TA_Pairs, Condition_Lists),
    append(Condition_Lists, Conditions),
    peeled_negation(Conditions, Positive_Conditions),
```

```
    sort(Positive_Conditions, Condition_Set).

%!  peeled_negation(+Xs:list, -Ys:list) is det.
%
%   True if Ys is Xs with one layer of not/1 peeled off.
%   not/1 represents negation.
%   If an element in Xs is negated, then that single negation is removed in Ys
%   If an element in Xs is not negated, then that element is left unchanged
%   in Ys.
peeled_negation([], []).
peeled_negation([X | Xs], [Y | Ys]) :-
    ( X = not Condition
    -> Y = Condition
    ;  Y = X
    ),
    peeled_negation(Xs, Ys).

%!  random_variables_world(+In:list, -Out:list) is multi.
%
%   Out is a row in the truth table derived from the variables
%   in In. Upon backtracking each row from the truth table is
%   generated.
%
%   if length(In, L), L>0 then random_variables_world/2 succeeds 2**L
%   times.
%   if length(In, 0) then random_variables_world/2 succeeds exactly
%   once with Out = [].
random_variables_world([], []).
random_variables_world([X | Xs], [X | Ys]) :-
    random_variables_world(Xs, Ys).
random_variables_world([X | Xs], [not(X) | Ys]) :-
    random_variables_world(Xs, Ys).
```

2.2 Network motifs interaction graphs and implementations

The following simulations reproduce the behavior of different network motifs introduced in [9, 10]. The investigations included simple regulation, negative and positive auto-regulation, two variants of the bi-fan motif, coherent and incoherent feed-forward loops and the single input module. We represent activated forms of a component A by A* in the interaction graphs and by "as" in input files.

In the ODE implementations, regulation was carried out using Hill functions:

$$\text{Hill}(X, K, n) = \frac{X^n}{X^n + K^n}$$

It runs from 0 to 1 when input is provided as X and from 1 to 0 for input at K. $X = K$ results in $\text{Hill}(X, K, n) = \frac{1}{2}$. The parameter n determines the slope of the sigmoidal curve.

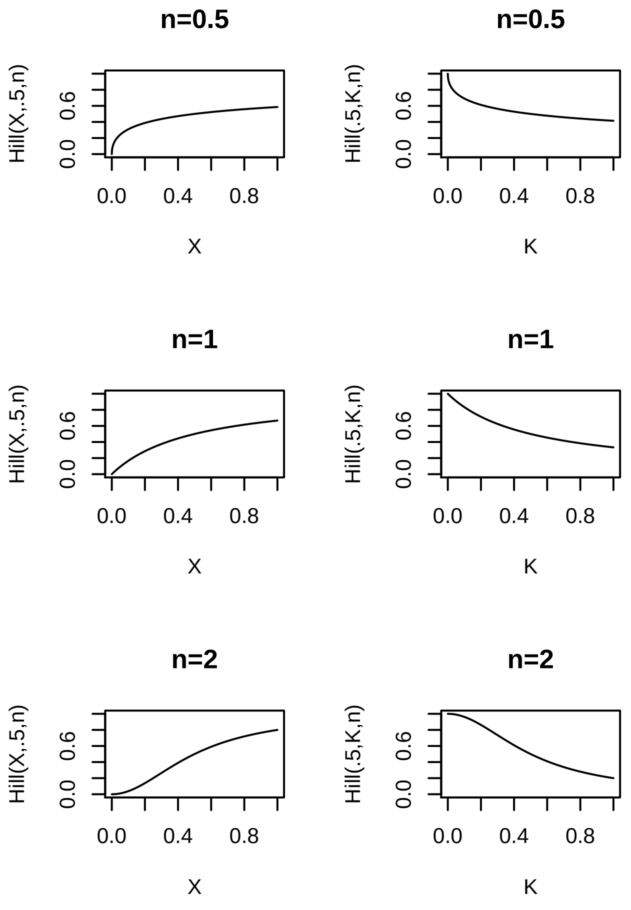

Figure N3: Hill functions for specified X, K and n.

2.2.1 Simple regulation, positive and negative autoregulation

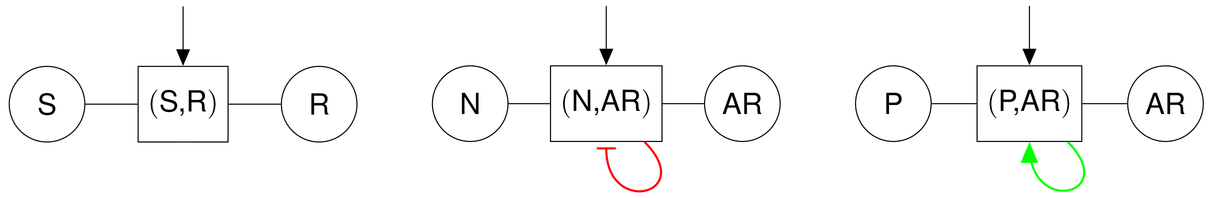

(a) In simple regulation, (S,R) is activated.

(b) In negative auto-regulation, an active interaction (N,AR) inhibits itself additionally to an activation by simple regulation.

(c) In positive auto-regulation, an active interaction (P,AR) activates itself additionally to an activation by simple regulation

Figure N4: Interaction graphs with rules for three basic regulatory motifs

These can be implemented as ODEs:

$$\begin{aligned}
\frac{d}{dt}[\text{SR}](t) &= 1 - [\text{SR}](t) \\
\frac{d}{dt}[\text{NAR}](t) &= 2\ \text{Hill}\left(1, [\text{NAR}](t), \frac{1}{2}\right) - [\text{NAR}](t) \\
\frac{d}{dt}[\text{PAR}](t) &= 2\ \text{Hill}\left([\text{PAR}](t), 1, \frac{1}{2}\right) - [\text{PAR}](t)
\end{aligned}$$

Together with the initial conditions

$$\begin{aligned}
[\text{SR}](0) &= 0.01 \\
[\text{NAR}](0) &= 0.01 \\
[\text{PAR}](0) &= 0.01
\end{aligned}$$

we obtain the dynamics as presented in Fig. 2 of the main text.

```
:- consult('../ProbRules.pl').

1.0 :: on.
0.0 :: off.
0.1 :: global_decay.

0.1 :: sr_rate.
0.3 :: nar_rate.
0.03:: par_rate.

0.0 :: interaction(s,r).
0.0 :: interaction(n,ar).
0.0 :: interaction(p,ar).

rule((s,r),[],sr_rate,on,'sr activation').

rule((n,ar),[],nar_rate,on,'nar activation').
rule((n,ar),[not (n,ar)],nar_rate,on,'nar autoregulation').

rule((p,ar),[],par_rate,on,'positive autoregulation activation').
rule((p,ar),[(p,ar)],par_rate,on,'positive autoregulation').

:- evaluation(100).
:- halt.
```

2.2.2 Symmetric bi-fan motif

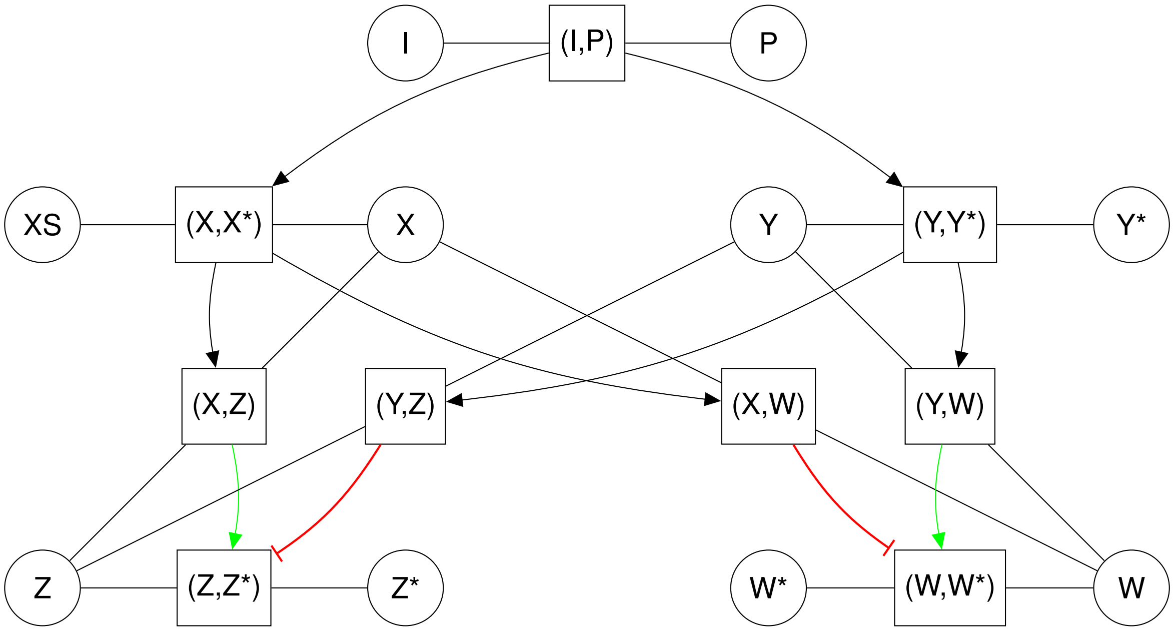

Figure N5: Interaction graph and rules of the symmetric bifan motif with two activating interactions and two inhibitory interactions.

These can be implemented as ODEs:

$$\begin{aligned}
[\text{IP}](t) &= \begin{cases} 1 & t \geq 6 \\ 0 & t < 6 \end{cases} \\
\frac{d}{dt}[\text{XXS}](t) &= [\text{IP}](t) - [\text{XXS}](t) \\
\frac{d}{dt}[\text{YYS}](t) &= [\text{IP}](t) - [\text{YYS}](t) \\
\frac{d}{dt}[\text{ZZS}](t) &= 7\,\text{Hill}\left([\text{XXS}](t), 5, 1\right)\ \text{Hill}\left(5, [\text{YYS}](t), 1\right) - [\text{ZZS}](t) \\
\frac{d}{dt}[\text{WWS}](t) &= 7\,\text{Hill}\left([\text{YYS}](t), 5, 1\right)\ \text{Hill}\left(5, [\text{XXS}](t), 1\right) - [\text{WWS}](t)
\end{aligned}$$

Together with the initial conditions

$$\begin{aligned}
[\text{XXS}](0) &= 0 \\
[\text{YYS}](0) &= 0 \\
[\text{ZZS}](0) &= 0 \\
[\text{WWS}](0) &= 0
\end{aligned}$$

we obtain the dynamics as presented in Fig. 2 of the main text.

```
:- consult('../ProbRules.pl').
1.0 :: on.
0.0 :: off.
0.1 :: global_attack.
0.001::local_attack.
0.1 :: global_decay.
0.0 :: interaction(i,p).
0.0 :: interaction(x,xs).
0.0 :: interaction(y,ys).
0.0 :: interaction(x,z).
0.0 :: interaction(x,w).
0.0 :: interaction(y,z).
0.0 :: interaction(y,w).
0.0 :: interaction(z,zs).
0.0 :: interaction(w,ws).
fixed(i,p,T) :- T>4, T<155, on.
fixed(i,p,_) :- off.
rule((x,xs),[(i,p)],global_attack,on,'xs').
rule((y,ys),[(i,p)],global_attack,on,'ys').
rule((x,z),[(x,xs)],global_attack,on,'xz').
rule((x,w),[(x,xs)],local_attack,on,'xw').
rule((y,z),[(y,ys)],local_attack,on,'yz').
rule((y,w),[(y,ys)],global_attack,on,'yw').
rule((z,zs),[(x,z),not (y,z)],global_attack,on,'zs').
rule((w,ws),[(y,w),not (x,w)],global_attack,on,'ws').
:- evaluation(250).
:- halt.
```

2.2.3 Asymmetric bi-fan motif

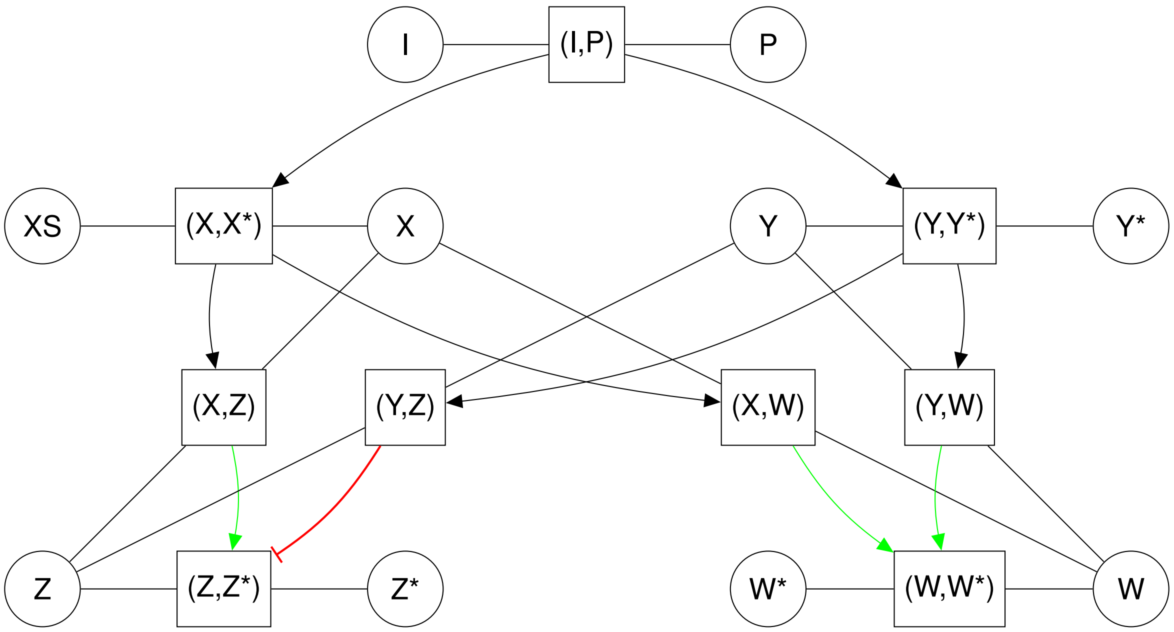

Figure N6: Interaction graph and rules of the asymmetric bifan motif with three activating interactions and one inhibitory interactions.

These can be implemented as ODEs:

$$
\begin{aligned}
[\text{IP}](t) &= \begin{cases} 1 & t \geq 6 \\ 0 & t < 6 \end{cases} \\
\frac{d}{dt}[\text{XXS}](t) &= [\text{IP}](t) - [\text{XXS}](t) \\
\frac{d}{dt}[\text{YYS}](t) &= [\text{IP}](t) - [\text{YYS}](t) \\
\frac{d}{dt}[\text{ZZS}](t) &= 7\,\text{Hill}\left([\text{XXS}](t), 5, 1\right)\ \text{Hill}\left(5, [\text{YYS}](t), 1\right) - [\text{ZZS}](t) \\
\frac{d}{dt}[\text{WWS}](t) &= 7\,\text{Hill}\left([\text{YYS}](t), 5, 1\right)\ \text{Hill}\left([\text{XXS}](t), 5, 1\right) - [\text{WWS}](t)
\end{aligned}
$$

Together with the initial conditions

$$
\begin{aligned}
[\text{XXS}](0) &= 0 \\
[\text{YYS}](0) &= 0 \\
[\text{ZZS}](0) &= 0 \\
[\text{WWS}](0) &= 0
\end{aligned}
$$

we obtain the dynamics as presented in Fig. 2 of the main text.

```
:- consult('../ProbRules.pl').
1.0 :: on.
0.0 :: off.
0.1 :: global_attack.
0.001::local_attack.
0.1 :: global_decay.
0.0 :: interaction(i,p).
0.0 :: interaction(x,xs).
0.0 :: interaction(y,ys).
0.0 :: interaction(x,z).
0.0 :: interaction(x,w).
0.0 :: interaction(y,z).
0.0 :: interaction(y,w).
0.0 :: interaction(z,zs).
0.0 :: interaction(w,ws).
fixed(i,p,T) :- T>4, T<155, on.
fixed(i,p,_) :- off.
rule((x,xs),[(i,p)],global_attack,on,'xs').
rule((y,ys),[(i,p)],global_attack,on,'ys').
rule((x,z),[(x,xs)],global_attack,on,'xz').
rule((x,w),[(x,xs)],local_attack,on,'xw').
rule((y,z),[(y,ys)],local_attack,on,'yz').
rule((y,w),[(y,ys)],global_attack,on,'yw').
rule((z,zs),[(x,z),not (y,z)],global_attack,on,'zs').
rule((w,ws),[(y,w),    (x,w)],global_attack,on,'ws').
:- evaluation(250).
:- halt.
```

2.2.4 Coherent feed-forward loop type I

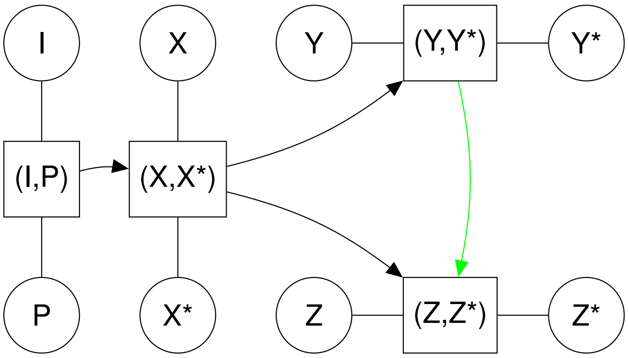

Figure N7: In the coherent feed-forward loop (FFL) type I motif (X,X*) activates (Y,Y*). Both jointly activate (Z,Z*).

These can be implemented as ODEs:

$$\begin{aligned}
[\text{IP}](t) &= \begin{cases} 1 & t < \frac{1}{5} \text{ or } t > 2 \text{ and } t < 6 \\ 0 & \text{otherwise} \end{cases} \\
\frac{d}{dt}[\text{XXS}](t) &= [\text{IP}](t) - [\text{XXS}](t) \\
\frac{d}{dt}[\text{YYS}](t) &= 2\,\text{Hill}\left([\text{XXS}](t), 1, 1\right) - [\text{YYS}](t) \\
\frac{d}{dt}[\text{ZZS}](t) &= 4\,\text{Hill}\left([\text{XXS}](t), 1, 1\right)\,\text{Hill}\left([\text{YYS}](t), 1, 1\right) - [\text{ZZS}](t)
\end{aligned}$$

Together with the initial conditions

$$\begin{aligned}
[\text{XXS}](0) &= 0 \\
[\text{YYS}](0) &= 0 \\
[\text{ZZS}](0) &= 0
\end{aligned}$$

we obtain the dynamics as presented in Fig. 2 of the main text.

```
:- consult('../ProbRules.pl').
1.0 :: on.
0.0 :: off.
0.05:: global_attack.
0.005::local_attack.
0.05:: global_decay.
0.0 :: interaction(i,p).
0.0 :: interaction(x,xs).
0.0 :: interaction(y,ys).
0.0 :: interaction(z,zs).
fixed(i,p,T) :- T>4, T<55, on.
fixed(i,p,T) :- T>199, T<600, on.
fixed(i,p,_) :- off.
rule((x,xs),[(i,p)],global_attack,on,'xs').
rule((y,ys),[(x,xs)],local_attack,on,'ys').
rule((z,zs),[(x,xs),(y,ys)],global_attack,on,'zs').
:- evaluation(800).
:- halt.
```

2.2.5 Incoherent feed-forward loop type I

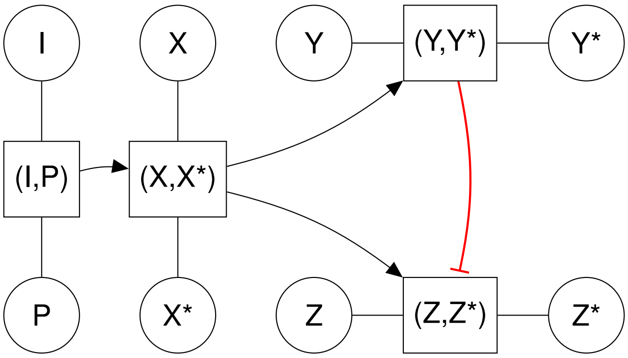

Figure N8: In the incoherent feed-forward loop (FFL) type I motif (X,X*) activates (Y,Y*). (Z,Z*) is activated while (X,X*) is active and (Y,Y*) is inactive.

These can be implemented as ODEs:

$$\begin{aligned}
[\text{IP}](t) &= 1 \\
\frac{d}{dt}[\text{XXS}](t) &= [\text{IP}](t) - [\text{XXS}](t) \\
\frac{d}{dt}[\text{YYS}](t) &= \text{Hill}\left([\text{XXS}](t), \frac{1}{3}, 5\right) - [\text{YYS}](t) \\
\frac{d}{dt}[\text{ZZS}](t) &= 3\ \text{Hill}\left([\text{XXS}](t), \frac{1}{3}, 5\right)\ \text{Hill}\left(\frac{1}{3}, [\text{YYS}](t), 5\right) - [\text{ZZS}](t)
\end{aligned}$$

Together with the initial conditions

$$\begin{aligned}
[\text{XXS}](0) &= 0 \\
[\text{YYS}](0) &= 0 \\
[\text{ZZS}](0) &= 0
\end{aligned}$$

we obtain the dynamics as presented in Fig. 2 of the main text.

```
:- consult('../ProbRules.pl').
1.0 :: on.
0.0 :: off.
0.5 :: global_attack.
0.05:: local_attack.
0.5 :: global_decay.
0.0 :: interaction(i,p).
0.0 :: interaction(x,xs).
0.0 :: interaction(y,ys).
0.0 :: interaction(z,zs).
fixed(i,p,_) :- on.
rule((x,xs),[(i,p)],global_attack,on,'xs').
rule((y,ys),[(x,xs)],local_attack,on,'ys').
rule((z,zs),[(x,xs),not (y,ys)],global_attack,on,'zs').
:- evaluation(100).
:- halt.
```

2.2.6 Single input module

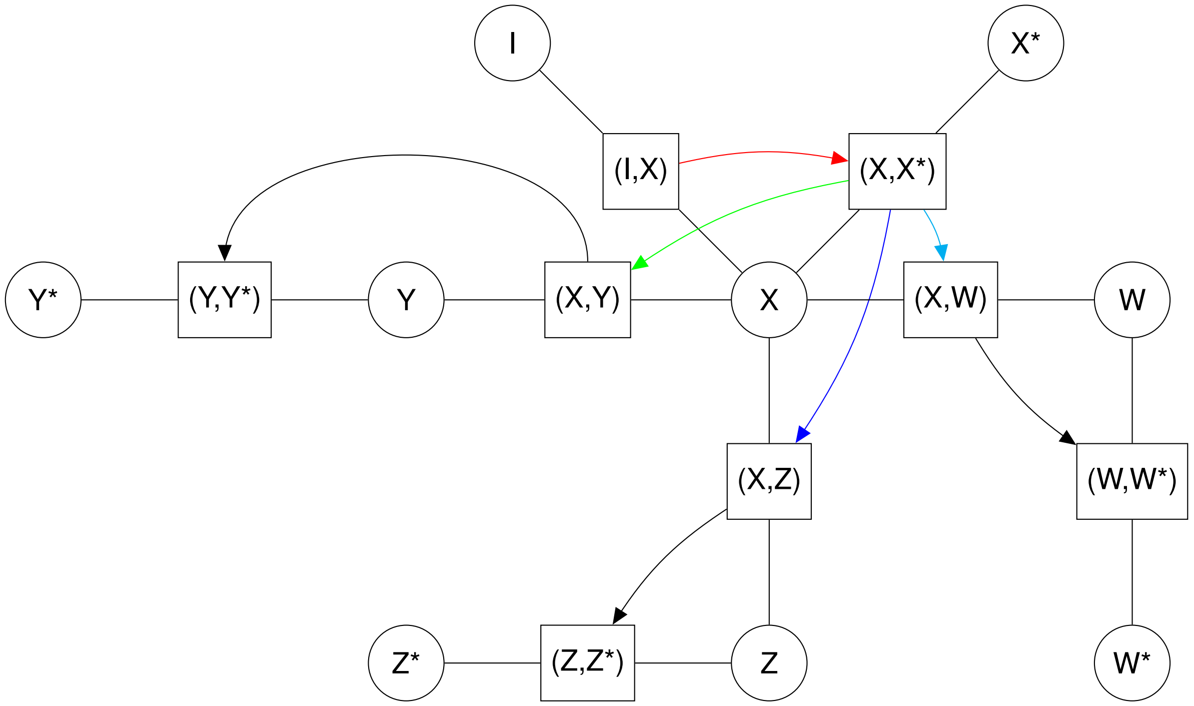

Figure N9: The single input module is a simple pattern in which one regulator activates a group of target genes with different strengths and thus speeds.

These can be implemented as ODEs:

$$\begin{aligned}
[\text{IP}](t) &= \begin{cases} 1 & t > 1 \text{ and } t < 6 \\ 0 & \text{otherwise} \end{cases} \\
\frac{d}{dt}[\text{XXS}](t) &= [\text{IP}](t) - [\text{XXS}](t) \\
\frac{d}{dt}[\text{XY}](t) &= \text{Hill}\left([\text{XXS}](t), \frac{1}{3}, 10\right) - [\text{XY}](t) \\
\frac{d}{dt}[\text{XZ}](t) &= \text{Hill}\left([\text{XXS}](t), \frac{1}{2}, 10\right) - [\text{XZ}](t) \\
\frac{d}{dt}[\text{XW}](t) &= \text{Hill}\left([\text{XXS}](t), \frac{2}{3}, 10\right) - [\text{XW}](t) \\
\frac{d}{dt}[\text{ZZS}](t) &= \text{Hill}\left([\text{XY}](t), \frac{3}{4}, 10\right) - [\text{ZZS}](t) \\
\frac{d}{dt}[\text{ZZS}](t) &= \text{Hill}\left([\text{XZ}](t), \frac{3}{4}, 10\right) - [\text{ZZS}](t) \\
\frac{d}{dt}[\text{WWS}](t) &= \text{Hill}\left([\text{XW}](t), \frac{3}{4}, 10\right) - [\text{WWS}](t)
\end{aligned}$$

Together with the initial conditions

$$\begin{aligned}
[\text{XXS}](0) &= 0 \\
[\text{XY}](0) &= 0 \\
[\text{XZ}](0) &= 0 \\
[\text{XW}](0) &= 0 \\
[\text{YYS}](0) &= 0 \\
[\text{ZZS}](0) &= 0 \\
[\text{WWS}](0) &= 0
\end{aligned}$$

we obtain the dynamics as presented in Fig. 2 of the main text.

```
:- consult('../ProbRules.pl').
1.0 :: on.
0.0 :: off.
0.1 :: global_attack.
0.09:: local_attack1.
0.03:: local_attack2.
0.01:: local_attack3.
0.1 :: global_decay.
0.0 :: interaction(i,p).
0.0 :: interaction(x,xs).
0.0 :: interaction(x,y).
0.0 :: interaction(x,z).
0.0 :: interaction(x,w).
0.0 :: interaction(y,ys).
0.0 :: interaction(z,zs).
0.0 :: interaction(w,ws).
fixed(i,p,T) :- T>49, T<350, on.
fixed(i,p,_) :- off.
rule((x,xs),[(i,p)],global_attack,on,'xs').
rule((x,y),[(i,p),(x,xs)],local_attack1,on,'xy').
rule((x,z),[(i,p),(x,xs)],local_attack2,on,'xz').
rule((x,w),[(i,p),(x,xs)],local_attack3,on,'xw').
rule((y,ys),[(x,y)],global_attack,on,'ys').
rule((z,zs),[(x,z)],global_attack,on,'zs').
rule((w,ws),[(x,w)],global_attack,on,'ws').
:- evaluation(700).
:- halt.
```

2.2.7 Using immediate rates disables ProbRules models to reproduce network motifs dynamics

In order to investigate advantages of the ProbRules models of network motifs over logical circuits we simplified the original network motifs implementations circumventing intermediate interactation activation probabilities. Instead, new target states are assigned immediately. This corresponds to setting all attack and decay rate values to 1.

Such simplifications for our ProbRules models of network motifs resulted in dynamics depicted in Fig. N10. Here, we specified the same interactions and interdependency rules as in the ProbRules implementations used for Fig. 2 and changed all attack and decay rates to 1.

The symmetric bifan and the incoherent type I feed-forward loop fail to show any activation at the corresponding outputs. Simple regulation, negative and positive autoregulation as well as the coherent type I feed-forward loop and the single input module fail to represent differential dynamics between the outputs. Remarkably, in the asymmetric bifan both effects can be observed for the outputs resulting in dynamics contradicting both ODE and ProbRules implementations with intermediate rates.

In summary, only by using intermediate interaction activations the ProbRules models are able to represent network motifs.

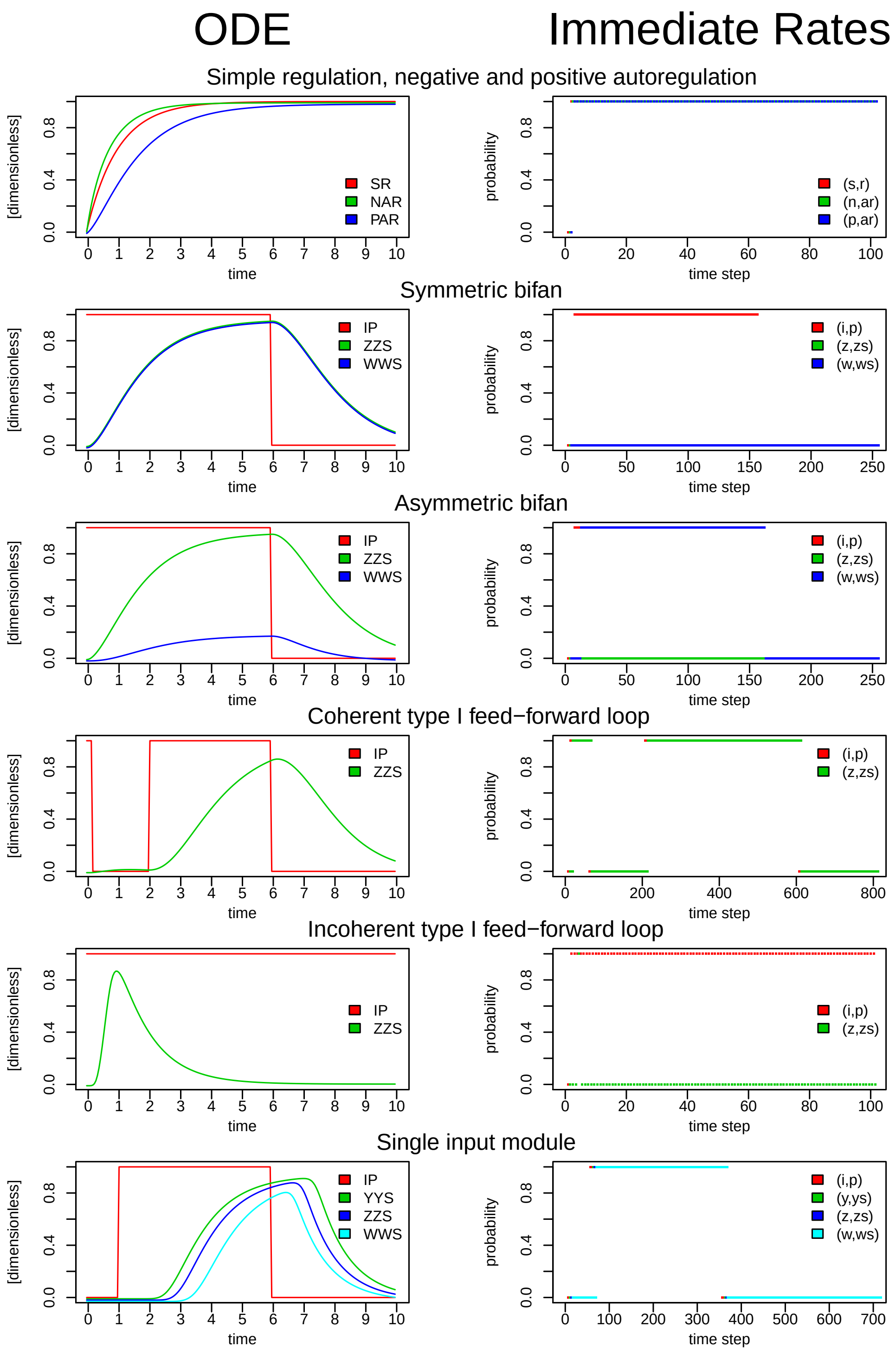

Figure N10: ODE models of network motifs on left side as in Fig. 2. ProbRules models of network motifs on right side with the same interactions and rules as in Fig. 2, but with immediate rates.

2.3 ProbRules model of Wnt/β-catenin and Wnt/JNK signaling

2.3.1 Logical relations in the Wnt signaling network

List of abbreviations

The following logical relations and rules describing Wnt/β-catenin and Wnt/JNK signaling use these abbreviations:

APC	Adenomatous polyposis coli
APC*	Phosphorylated adenomateous polyposis coli
axin*	Phosphorylated axin
β-cat*	Phosphorylated β-catenin
β-cat**	Double phosphorylated β-catenin
β-catUB	Ubiquitinated β-catenin
c-Jun	jun proto-oncogene
CKIα, γ, ε	Casein kinase I; α, γ, and ε isoforms are considered as indicated
DAG	Diacylglycerol
Dsh	Dishevelled
Dsh*	Phosphorylated dishevelled
Dsh**	Double phosphorylated dishevelled
DOCK4	Dedicator of cytokinesis 4 (a guanine nucleotide exchange factor)
E1/2/3	subunits of the ubiquitination system
Fz	Frizzled
Gα, Gβ, Gγ	Guanine nucleotide binding protein (G-protein) subunits α, β, γ
GαGDP	G-protein α subunit bound GDP (inactive)
GαGTP	G-protein α subunit bound GTP (active)
Gβγ	G-protein β/γ dimer
GBP/FRAT	GSK3 binding protein (also named FRAT)
GDP	guanosine diphosphate
GEF	Guanine nucleotide exchange factor
GSK	Glykogen synthase kinase 3-β
GTP	guanosine triphospahte
IP3	Inositol triphosphate
JNK	c-Jun N-terminal kinase
JNK1*	Phosphorylated c-Jun N-terminal kinase 1
JNK2*	Phosphorylated c-Jun N-terminal kinase 2
LEF	Lymphoid enhancer factor
LRP	Lipoprotein receptor related protein -5 or -6
LRP*	phosphorylated lipoprotein receptor related protein -5 or -6
MLK	Mixed linkage kinase
PIP	Phosphatidylinositol (4,5)-bisphosphate
PKCδ	Calcium sensitive protein kinase C δ
PKCδ *	Phosphorylated calcium sensitive protein kinase C δ
PLCβ	Phospholipase C β
PP1	Protein phosphatase 1
Rac	ras-related C3 botulinum toxin substrate (rho family, small GTP binding protein Rac)
Rho	Ras homolog
Ror2	Receptor tyrosine kinase-like orphan receptor 2
TCF	T-cell factor
Tiam1	T-cell lymphoma invasion and metastasis 1
Ub	Ubiquitin

Logical relations in the Wnt/β-catenin pathway

In absence of a Wnt ligand

Logical relation 1	In absence of Wnt, Fz does not interact with LRP.
Speed	fast
Comment	Both Fz [11, 12, 13, 14, 15] and LRP-5/6 [16, 17, 18, 19] have been shown to interact with Wnt and to be responsible for Wnt induced signal transduction. Fz and LRP do not interact in absence of a Wnt ligand [20]. It has been shown that Wnt interacts with both, Fz and LRP that are brought in close proximity by this binding [15, 18, 20, 21].

Logical relation 2	In absence of Wnt, Fz interacts with GαGDP and Gβγ
Speed	average
Comment	Fz receptors are seven-pass transmembrane receptors. A whole series of publications indicate that Wnt signaling is likely G-protein coupled [22, 23, 24, 25, 26, 27, 28]. Data from [26] strongly suggest a direct interaction of Fz receptors with heterotrimeric G-proteins. Logical relation 2 represents the common view of signaling through heterotrimeric G-protein coupled receptors.

Logical relation 3	In absence of Wnt, LRP does not interact with axin.
Speed	average
Comment	LRP has been shown to interact with axin upon Wnt stimulation [29, 30]. Wnt proteins induce the translocation of axin to the plasma membrane and increase the interaction of axin with LRP. This logical relation formalizes the situation in absence of a Wnt ligand.

Logical relation 4	In absence of Wnt, LRP does not interact with CKIγ or GSK3.
Speed	fast
Comment	LRP has been shown to interact with CKIγ and GSK-3β [31, 32]. Both enzymes are able to modify LRP. This logical relation formalizes the situation in absence of a Wnt ligand.

Logical relation 5	In absence of Wnt, dsh does not interact with Fz.
Speed	fast
Comment	Dsh can interact with Fz [33, 34, 35]. It is thought, that this interaction occurs after Wnt stimulation, i.e. dsh is translocated to the membrane upon ligand binding [36, 37] (see below for further details).

Logical relation 6	In absence of Wnt, the destruction complex forms in the cytosol and interacts with β-catenin.
Speed	average
Comment	This logical relation describes the initial formation of the destruction complex [38, 39, 40, 41, 42, 43, 44]. Axin can also be replaced by the close homolog axin2/axil/conductin [45, 46]

Logical relation 7	a) If GSK interacts with axin, and axin interacts with APC, APC interacts with GSK3.
Speed	fast
	b) If GSK interacts with axin and APC, and axin interacts with APC, APC is phosphorylated (by GSK3) to form APC*.
Speed	fast
	c) Phosphorylated APC* interacts with axin and β-catenin.
Speed	fast
	d) If GSK3 interacts with axin, axin is transferred into axin*.
Speed	fast
	e) If APC* interacts with β-catenin, then β-catenin interacts with axin*.
Speed	fast
Comment	These steps describe the formation of the *mature* destruction complex, considering that GSK-3β also phosphorylates APC, which is favorable for the degradation of β-catenin [44]. This set of rules also indicates that the destruction complex is stable in case of APC modification. Axin also can be phosphorylated by GSK-3β [47, 48] and this phosphorylation was shown to be required for the interaction between axin and β-catenin [49].

Logical relation 8	a) If CKIα interacts with axin, and axin interacts with β-catenin, then CKIα interacts with β-catenin.
Speed	fast
	b) If CKIα interacts with β-catenin, then β-catenin is modified to form β-cat*.
Speed	fast
	c) In the presence of the mature destruction complex, GSK3 can interact with β-cat* and modify it into β-cat** (that is GSK phosphorylates β-cat* a total of three times).
Speed	fast
	Inhibition of phosphorylation
	d) If β-cat** is formed, β-cat** can interact with E1/2/3.
Speed	average
	- - - *or* - - -
	Inhibition of ubiquitination
	d) If β-cat** is formed and axin does not interact with LRP* at the membrane, β-cat** can interact with E1/2/3.
Speed	average
	Remark: One recent publication [50] postulates, that upon Wnt-induced interaction of axin with phosphorylated LRP* at the plasma membrane, E1/2/3 dissociates from the destruction complex, which is included in this version of rule 8d).
	e) If β-cat** interacts with E1/2/3, β-cat** is modified into β-catUb.
Speed	fast
	f) β-catUb is degraded, therefore β-catenin cannot accumulate.
Speed	average
Comment	This logical relation describes that β-catenin is marked for degradation through ubiquitination and subsequently degraded in the proteasome [38, 51, 52]. As a pre-requisite for ubiquitination, β-catenin is phosphorylated through CKIα and GSK-3β in a dual kinase mechanism [36, 50]. Initially, β-catenin is phosphorylated by CKIα at Ser45. This modification is represented in this model as β-cat*. After this pre-phosphorylation, GSK-3β modifies residues Ser33, Ser37, and Thr41. This modified β-catenin is represented by β-cat**. This phosphorylated variant of β-catenin can be ubiquitinated through action of E1/2/3 to form β-catUb, which then is degraded through the proteasome.

Logical relation 9 — If β-catenin is accumulating and interacts with JNK2, β-catenin can be translocated in the nucleus and interact with LEF.

Speed — slow

Comment — This logical relation indicates that free β-catenin which is not degraded through the proteasome can interact with transcription factors of the TCF/LEF family [53, 54, 55]. Moreover, recently it has been shown that RAC and JNK activity are also required for canonical wnt signaling activity [56, 57, 58], which is in accordance with our own experimental findings. This cooperative effect likely involves RAC1 (and maybe RAC3) and JNK2 and may be mediated via control of β-catenin stabilization [59] or nuclear localization [58]. Another possibility is a potential function of c-jun [57] or RAC (together with its Rho-GEF Tiam1) [56] as transcriptional co-activators of TCF/LEF.

Remark: This positive effect of RAC and JNK on canonical signaling activity is also expressed by logical relation j19 b).

Logical relation 10 — If β-catenin in the nucleus interacts with LEF, LEF interacts with the DNA.

Speed — average

Comment — This logical relation formalizes the observation, that β-catenin functions as a transcriptional co-activator for TCF/LEF proteins [55, 60, 61, 62, 63, 64, 65]. The interaction of LEF/β-catenin with DNA results in a transcriptional response that was used as a read out for the pathway.

In presence of Wnt

Logical relation 11	In presence of Wnt, Wnt, Fz and LRP form a trimeric complex.
Speed	average
Comment	It has been shown that Wnt interacts with both, Fz and LRP that are brought in close proximity by this binding [15, 18, 20, 21].

Logical relation 12	If Wnt interacts with LRP, LRP interacts with CKIγ and GSK3.
Speed	slow

Logical relation 13	If LRP interacts with CKIγ/GSK3, LRP is modified and activated to form LRP*.
Speed	fast
Comment	LRP has been shown to interact with CKIγ and GSK-3β [31, 32]. Both enzymes are able to modify LRP upon ligand stimulation.

Logical relation 14	a) If Wnt interacts with Fz, GαGDP is transferred into GαGTP. G$\beta\gamma$ still interacts with Gα at this step.
Speed	slow
	b) If GαGDP is transferred into GαGTP, G dissociates into GαGTP and G$\beta\gamma$. Both leave the receptor.
Speed	average
Comment	This logical relation summarizes the common view of how activation of G-protein coupled receptors is transferred through the different subunits of heterotrimeric G-proteins into the cell.

Logical relation 15	a) GαGTP is hydrolyzed to GαGDP.
Speed	average
	b) Inactivated GαGDP interacts with G$\beta\gamma$ and Fz.
Speed	fast
Comment	GαGTP can be inactivated to GαGDP. GαGDP can interact again with G$\beta\gamma$ and Fz. This rule summarizes a common mechanism how signaling through heterotrimerc G-proteins can be turned off again.

Logical relation 16	a) If GαGTP is released, GαGTP interacts with dsh.
Speed	average
	b) If GαGTP interacts with dsh, dsh* is formed.
Speed	fast
Comment	This logical relation indicates the formation of an activated form of dishevelled, named dsh*. It has been shown that this requires activated Gα subunits [26]. The precise mechanism of this activation is unknown so far but likely involves CKIγ. CKIγ is one kinase that phosphorylates dsh and is required for Wnt induced stabilization of β-catenin [66, 67]. Dishevelled has also been shown to interact with G$\beta\gamma$ [68].<br>If inactivated GαGDP interacts again with G$\beta\gamma$ before activation of dsh, no downstream signaling can occur.
Hint	Logical relations 12/13 and 14-16 are active at the same time.
Comment	For transduction of the Wnt signal through the Wnt/β-catenin signaling pathway, Wnt interacts with both, LRP and Fz. This logical relation indicates that the different branches through LRP and Fz occur simultaneously.

Logical relation 17	a) If dsh* is formed, dsh* interacts with axin.
Speed	average

Inhibition of phosphorylation

	b) If dsh* is formed, dsh* can interact with GBP/FRAT.
Speed	average
	c) If dsh* interacts with axin and GBP, GBP interacts with GSK3.
Speed	average
	d) If GSK3 interacts with GBP, then GSK3 does not interact with axin, APC or β-catenin.
Speed	fast
Comment	There are two branches described, how β-catenin phosphorylation in the destruction complex can be inhibited. This set of rules summarizes how β-catenin phosphorylation is inhibited through inactivation of GSK-3β via dsh and GBP/FRAT. Dsh is required for Wnt signal transduction [36, 40, 66, 69]. The phosphorylated form of dsh (likely phosphorylated by CKIγ) has a high affinity to GBP/FRAT that can interact with GSK-3β and can inactivate this enzyme [67, 70, 71]. The consequence of GSK-3β inactivation is that β-catenin accumulates; logical relations 9 and 10 will become effective!

- - - or - - -

Inhibition of ubiquitination

	Remark: This logical relation does not make use of logical subrelations 17 b-d.
Comment	The signaling mechanism through inhibition of β-catenin ubiquitination as postulated by Li et al. [50] does not assume inhibition of β-catenin phosphorylation through inactivation of GSK-3β.

Logical relation 18	a) If dsh* is formed, dsh* can interact with Fz.
Speed	slow
	b) If phosphorylated axin* interacts with phosphorylated dsh*, axin* can interact with phosphorylated LRP*.
Speed	average
	c) If dsh* interacts with Fz and axin, and LRP*interacts with Fz and axin, then axin interacts with PP1.
Speed	average
Comment	Phosphorylated axin can interact with LRP, in particular with the activated form LRP* [30, 31, 32, 37, 49]. Dsh interacts with Fz receptors [21, 33] and is required for proper translocation of axin to the membrane [37].

Inhibition of phosphorylation

	d) If phosphorylated axin* interacts with PP1, axin* is dephosphorylated (i.e. it cannot interact with β-catenin).
Speed	fast

- - - or - - -

Inhibition of ubiquitination

	d) If axin interacts with LRP, E1/2/3 dissociates from β-catenin.
Speed	fast

Logical relation 19

Inhibition of phosphorylation

a) If the destruction complex is inhibited and β-catenin is not degraded, β-catenin can accumulate.

Speed average

This set of rules summarizes the second possibility how β-catenin phosphorylation is inhibited, through a weakening of the interaction between β-catenin and axin, when axin is dephosphorylated by PP1 in response to the Wnt signal [49].

- - - or - - -

Inhibition of ubiquitination

a) If E1/2/3 dissociates from the destruction complex upon Wnt stimulation and β-catenin thus is not degraded, β-catenin can accumulate.

Speed average

Remark: Upon association of axin with phosphorylated LRP at the membrane, dissociation of E1/2/3 from the destruction complex was observed in one recent study [50]. Upon this dissociation of E1/2/3 from the destruction complex, β-catenin is no longer ubiquitinated and degraded [72].

Comment Consequence of both versions of logical relations 18d) and 19a) is that β-catenin will accumulate and therefore logical relations 9 and 10 will become effective.

Concerning logical relation 19a) knockdown data obtained in this study hints, that Rac1 plays a role in β-catenin stabilization (independent of JNK) as a loss of Rac1 (but not JNK) diminishes the increase in free β-catenin after activation of canonical Wnt signaling (see also logical relation j17). This influence could be mediated e.g. via the guanine exchange factor DOCK4 [59].

Remark: Logical relation 19a) in both versions now assumes a positive influence of Rac on the amount of free β-catenin that is necessary for the model to fit the experimental data obtained in this study.

b) If β-catenin accumulates, the high β-catenin concentration will feed back to re-activate the destruction complex.

Speed average

Comment Two recent studies [73] as well as our own experimental data show, that upon Wnt stimulation the amount of phosphorylated β-catenin is markedly decreased for a short time but subsequently is recovered quickly. Based on simulation results obtained with an ODE model of β-catenin synthesis, phosphorylation and degradation, Hernandez et al. [74] hypothesize, that this transient drop occurs due to incomplete inactivation of the destruction complex, which thus can be re-activated at high β-catenin levels.

Logical relation 20	If one of the scaffold proteins axin or APC is missing, no destruction complex can be formed.
Speed	fast
Comment	This logical relation formalizes that the scaffold proteins axin and APC are critical components of the destruction complex. They are necessary to bring GSK-3β and β-catenin in close proximity thus enabling β-catenin phosphorylation (reviewed e.g. in [73]).

Logical relation 21	Independent of the presence or absence of Wnt, β-catenin is newly synthesized.
Speed	average
Comment	To be able to model the comparably fast turnover of β-catenin appropriately, besides the degradation through the proteasome (see logical relation 8f), also the β-catenin synthesis is modeled explicitly as in previous ODE-based models of Wnt signaling (e.g. [74, 75]).

Logical relations in the Wnt/JNK pathway

In absence of a Wnt ligand

Logical relation j1	In absence of Wnt, dsh does not interact with Fz.
Speed	fast
Comment	Dsh can interact with Fz [21, 33, 35]. It is thought that this interaction occurs upon Wnt stimulation, i.e. dsh is translocated to the membrane upon ligand binding [33, 35].

Logical relation j2	In absence of Wnt, Fz does not interact with Ror2.
Speed	fast
Comment	Ror2 was found to associate with Fz and participate in JNK activation upon Wnt stimulation [76].

Logical relation j3	In absence of Wnt, Fz interacts with GαGDP and G$\beta\gamma$.
Speed	average
Comment	A series of publications [22, 23, 25, 26, 27] indicate that Wnt signaling is likely G-protein coupled. Data from [26] strongly suggest a direct interaction of Fz receptors with heterotrimeric G-proteins.

Logical relation j4	In absence of Wnt, part of the Rac-GTPases is already present in the active, GTP-bound form.
Speed	average
Comment	It is known that small RhoGTPases of the Rho and Rac family and the subsequent MAPK (mitogen-activated protein kinase) cascade can be activated by different cytokines, coupling to tyrosine-kinase and G-protein coupled receptors (see e.g. [77, 78]). Therefore it has to be assumed that even in the absence of Wnt ligands a certain amount of Rac will be present in the active GTP-bound state as long as other cytokines are present (e.g. from FCS (fetal calf serum) in the cell culture medium).

In presence of Wnt

Logical relation j5	In the presence of Wnt, Wnt interacts with Fz and Ror2.
Speed	average
Comment	It has been shown that Wnt interacts with both Fz [15] and Ror2 [76], with the latter two forming a complex [76].

Logical relation j6	a) If Wnt interacts with Fz, $G\alpha$GDP is transferred into $G\alpha$GTP. $G\beta\gamma$ still interacts with $G\alpha$ at this step.
Speed	slow
	b) If $G\alpha$GDP is transferred into $G\alpha$GTP, G dissociates into $G\alpha$GTP and $G\beta\gamma$. Both leave the receptor.
Speed	average
Comment	This logical relation summarizes the common view of how activation of G-protein coupled receptors is transferred through the different subunits of heterotrimeric G-proteins into the cell.

Logical relation j7	a) $G\alpha$GTP is hydrolyzed to $G\alpha$GDP.
Speed	average
	b) Inactivated $G\alpha$GDP interacts with $G\beta\gamma$ and Fz.
Speed	fast
Comment	$G\alpha$GTP can be inactivated to $G\alpha$GDP. $G\alpha$GDP can interact again with $G\beta\gamma$ and Fz. This logical relation summarizes a common mechanism how signaling through heterotrimerc G-proteins can be turned off again.

Logical relation j8	If active $G\alpha$GTP is released, $G\alpha$GTP interacts with dsh.
Speed	average
Comment	It has been shown that the activation of dsh requires activated $G\alpha$ subunits [26], although the precise mechanism for this activation is unknown so far. If inactivated $G\alpha$GDP interacts again with $G\beta\gamma$ before activation of dsh, no downstream signaling can occur.

Logical relation j9	If G dissociates into GαGTP and Gβγ and Gβγ does not interact anymore with Gα or Fz, Gβγ interacts with PLCβ.
Speed	average
Comment	Gβγ has been shown to activate PLCβ [79]. If Gβγ interacts again with GαGDP before activation of PLCβ no downstream signaling can occur [79].

Logical relation j10	If free Gβγ interacts with and activates PLCβ, PLCβ forms DAG.
Speed	fast
Comment	PLCβ is known to hydrolyze PIP2 into water-soluble IP3 and lipid-soluble DAG (reviewed e.g. in [80]).
Logical relation j11	a) DAG can interact with PKCδ.
Speed	average
	b) If DAG interacts with PKCδ, PKCδ is activated to form PKCδ*.
Speed	fast
Comment	As described in [81], PKCδ belongs to the PKC subfamily of novel PKCs. This class of PKCs generally binds to and is activated by DAG (comp. [81]) that is produced on the membrane upon extracellular signaling.

Logical relation j12	If PKCδ is activated by DAG to form PKCδ*, PKCδ can interact with dsh.
Speed	average
Comment	PKCδ was found to form a complex with dsh [81] and dsh also was shown to be phosphorylated by PKCδ [82]. Logical relation j12 reflects the essential role PKCδ plays in the Wnt/JNK pathway by activating dsh [81].

Logical relation j13	If Wnt interacts with Fz and Ror2, Ror2 interacts with Fz.
Speed	average
Comment	Ror2 has been shown to interact with Wnt-5a [76, 83, 84] and to form a complex with Fz [76]. Also Wnt is known to interact with Fz (see logical relation j5) [15].

Logical relation j14	If Fz interacts with Ror2, Fz interacts with dsh.
Speed	slow
Comment	Fz can interact with dsh upon Wnt stimulation (see logical relation j1) [21, 33, 35] and form a complex with Ror2 (see rule j13) [76]. Logical relation 13 represents the fact that Ror2 is likely involved in JNK activation [76], which is thought to occur through activation of dsh [13, 34, 85, 86].

Logical relation j15	If dsh interacts with Fz and dsh interacts with GαGTP and dsh interacts with PKCδ, dsh is activated to form dsh**.
Speed	average
Comment	It has been shown that the activation of dsh requires activated Gα subunits (see also logical relation j8) [26]. Moreover PKCδ - which forms a complex with dsh - also has been found to be important for the signaling from Fz to dsh [81].

Logical relation j16	If dsh** is formed, dsh can interact with Rac.
Speed	average
Comment	Dsh has been observed to form a Wnt-induced complex with Rac [87, 88] which has been found to function downstream of dsh in planar polarity signaling [89].

Logical relation j17 — a) If dsh interacts with Rac, Rac is activated to form RacGTP.

Speed — fast

b) Only active GTP-bound Rac can interact with JNK1 and JNK2 and influence β-catenin.

Speed — average

Remark: logical relations j17 b) and c) include a connection from Rac to β-catenin (possibly via DOCK4, but not including JNK) that is necessary for the model to fit the experimental data obtained in this study.

Comment — Logical relations j17 a) and b) summarize the common view how small RhoGTPases (like Rac) are activated: They are transferred from an inactive GDP-bound to an active GTP-bound state by Rho-GEFs in response to a stimulus and this GTP-bound form is required for further signal transduction. Concerning logical relation j17b) and c) knockdown data obtained in this study hints, that Rac1 plays a role in β-catenin stabilization independent of JNK, as a loss of Rac1 (but not JNK) diminishes the increase in free β-catenin after activation of canonical Wnt signaling. Therefore a connection between Rac and β-catenin independent of JNK has to be assumed in the model for the simulation results to fit the experimental data. This link could e.g. function via the guanine exchange factor DOCK4 [59].

Logical relation j18 — If Rac interacts with JNK1 and JNK2, they are activated to form JNK1* and JNK2*.

Speed — fast

Comment — Logical relations j17 and j18 indicate that dsh, more exactly the c-terminal half of dsh which contains the DEP (EGL-10 and pleckstrin) domain [85], has been found to activate the JNK pathway [85, 86] with the small GTPase Rac acting downstream of dsh. The signal transduction from dsh to JNK, reflected in logical relations j17 and j18, likely occurs through Rac, as a full correlation between JNK and Rac activation has been observed in different studies [77, 78, 87]. Possibly MLK3 serves as a link between Rac and JNK as shown in [90]. However there also exists data considering Rac unlikely to play a significant role in dsh-induced JNK activation [69].

Logical relation j19	a) If JNK1 and JNK2 are activated, they can interact with β-catenin.
Speed	average
	b) If JNK2 interacts with free β-catenin, β-catenin can interact with LEF.
Speed	slow
Comment	It has been shown that Rac and JNK activity are required for canonical Wnt signaling activity [56, 57, 58] which is in accordance with our own experimental findings. This cooperative effect likely involves Rac1 (and maybe Rac3) and JNK2 and may be mediated via control of β-catenin stabilization [59] or nuclear localization [58]. Another possibility is a potential function of c-jun [57] or Rac (together with its Rho-GEF Tiam1) [56] as transcriptional co-activators of TCF/LEF.<br><br>Remark: This positive effect of RAC and JNK on canonical signaling activity is also expressed in logical relation 10.
	c) IF JNK1 is activated, JNK1 can activate GSK3.
Speed	average
	d) If JNK1 interacts with β-catenin and GSK3, and β-catenin is in the destruction complex, β-catenin can be phosphorylated by GSK3.
Speed	average
Comment	Contrary to the above mentioned findings there also exists data that activation of JNK can inhibit the transcription of canonical Wnt target genes by preventing β-catenin accumulation through induction either of β-catenin nuclear export [91] or β-catenin phosphorylation by GSK-3β [92].

2.3.2 ProbRules source code for the Wnt signaling model

```
%%% --------------
%%%  Wnt Rule Set
%%% --------------

:- consult('../ProbRules.pl').

%% Target probabilities for the rules:
1.0 :: on.
0.0 :: off.

%% Global attack and decay rates:
0.15 :: syn_rate.
0.6 :: global_attack.
0.3 :: global_decay.

%% Parameters for perturbations:
%%    (required only for the simulation of perturbations,
%%    i.e. knockdown, inhibition, or constitutive
%%    activation)
0.0 :: knockdown.
0.0 :: inhibition.
1.0 :: constact.


% -----------------------------------------------------------


%% Definition of interactions
%%    with their initial probabilities (used for decay)


%%% Wnt/beta-catenin branch %%%

0.0 :: interaction(ligand,wnt).
0.0 :: interaction(wnt,fz).
0.0 :: interaction(wnt,lrp).

0.0 :: interaction(lrp,lrpp).
0.0 :: interaction(ga,gtp).
0.0 :: interaction(ga,dsh).
```

```
0.0 :: interaction(gbg,gbg).
0.0 :: interaction(dsh,dshp).
0.0 :: interaction(bcat,e123).
0.0 :: interaction(bcat,bcatub).
0.0 :: interaction(dshp,axin).
0.0 :: interaction(axin,pp1).
0.0 :: interaction(dsh,gbp).
0.0 :: interaction(gbp,gsk3).

1.0 :: interaction(new,bcat).
0.5 :: interaction(bcat,bcat).
0.0 :: interaction(bcat,bcatfree).
0.0 :: interaction(bcat,lef).
0.0 :: interaction(lef,dna).

% RULE 1: In absence of Wnt, Fz does not interact with LRP.
0.0 :: interaction(fz,lrp).

% RULE 2: In absence of Wnt, Fz interacts with GaGDP and Gbg.
1.0 :: interaction(fz,ga).
1.0 :: interaction(fz,gbg).
1.0 :: interaction(ga,gdp).
1.0 :: interaction(ga,gbg).

% RULE 3: In absence of Wnt, LRP does not interact with axin.
0.0 :: interaction(lrp,axin).

% RULE 4: In absence of Wnt, LRP does not interact with CKIg
%         or GSK3.
0.0 :: interaction(lrp,ck1g).
0.0 :: interaction(lrp,gsk3).

% RULE 5: In absence of Wnt, dsh does not interact with Fz.
0.0 :: interaction(fz,dsh).

% RULE 6: In the absence of Wnt, the destruction complex
% can be formed in the cytosol and interact with b-catenin.
1.0 :: interaction(gsk3,axin).
1.0 :: interaction(axin,ck1a).
0.5 :: interaction(axin,apc).
0.5 :: interaction(apc,gsk3).
```

```
0.5 :: interaction(axin,bcat).
0.5 :: interaction(apc,bcat).
0.5 :: interaction(gsk3,bcat).
0.5 :: interaction(ck1a,bcat).

0.0 :: interaction(apc,apcp).
0.0 :: interaction(axin,axinp).
0.0 :: interaction(bcat,bcatp).
0.0 :: interaction(bcat,bcatpp).


%%% Wnt/JNK branch %%%

0.0 :: interaction(ligandj,wntj).
0.0 :: interaction(wntj,fzj).
0.0 :: interaction(wntj,ror2).
0.0 :: interaction(pkcd,dshj).
0.0 :: interaction(dshj,dshjpp).
0.0 :: interaction(dshj,rac).

0.0 :: interaction(gaj,gtp).
0.0 :: interaction(gaj,dshj).
0.0 :: interaction(gbgj,gbgj).
0.0 :: interaction(gbgj,plcbj).
0.0 :: interaction(plcbj,dag).
0.0 :: interaction(dag,pkcd).
0.0 :: interaction(pkcd,pkcdp).

0.0 :: interaction(rac,bcat).
0.0 :: interaction(rac,jnk1).
0.0 :: interaction(rac,jnk2).
0.0 :: interaction(jnk1,jnk1p).
0.0 :: interaction(jnk2,jnk2p).
0.0 :: interaction(jnk2,bcat).
0.0 :: interaction(jnk1,gsk3).
0.0 :: interaction(jnk1,bcat).

% RULE j1: In absence of Wnt, dsh does not interact with Fz.
0.0 :: interaction(fzj,dshj).

% RULE j2: In absence of Wnt, Fz does not interact with Ror2.
```

```
0.0 :: interaction(fzj,ror2).

% RULE j3: In absence of Wnt, Fz interacts with GaGDP and
%          Gbg.
1.0 :: interaction(fzj,gaj).
1.0 :: interaction(fzj,gbgj).
1.0 :: interaction(gaj,gbgj).
1.0 :: interaction(gaj,gdp).

% RULE j4: In absence of Wnt, part of the Rac-GTPases is
%          already present in the active, GTP-bound form.
0.5 :: interaction(rac,gtp).
0.5 :: interaction(rac,gdp).


% ------------------------------------------------------------

% INPUT signal

%%% Wnt/beta-catenin branch %%%

fixed(ligand,wnt,T) :- T> 100, T< 175, on.
fixed(ligand,wnt,_) :- off.

%%% Wnt/JNK branch %%%

fixed(ligandj,wntj,T) :- T> 100, T< 175, on.
fixed(ligandj,wntj,_) :- off.

% ------------------------------------------------------------


%% This rules replace the default decay for input interactions.
0.02 :: slow_attack.
rule((ligand,wnt),[(ligand,wnt)],slow_attack,off,'input decay canonical').
rule((ligandj,wntj),[(ligandj,wntj)],slow_attack,off,'input decay jnk').


%% ----------
%%  RULE SET
%% ----------
```

```
%% Rules are defined as:
%     "rule ((target interaction), [source interaction(s)],
%       attack rate, target probability, label)."


%%% Rules Wnt/beta-catenin %%%

% RULE 7a: If GSK3 interacts with axin, and axin interacts
%          with APC, APC interacts with GSK3.
rule((apc,gsk3),[(gsk3,axin),(axin,apc)],
    global_attack,on,'7a').

% RULE 7b: If GSK3 interacts with axin and APC, and axin
%          interacts with APC, APC is phosphorylated (by
%          GSK3) to form APCp [=APC*].
rule((apc,apcp),[(gsk3,axin),(axin,apc),(apc,gsk3)],
    global_attack,on,'7b').

% RULE 7c: Phosphorylated APCp interacts with axin and
%          b-catenin. (interaction probabilities up to 1)
rule((apc,bcat),[(apc,apcp),(bcat,bcat)],global_attack,on,'7c_1').
rule((axin,apc),[(apc,apcp)],global_attack,on,'7c_2').

% RULE 7d: If GSK3 interacts with axin,
%     axin is transferred into axinp [=axin*].
rule((axin,axinp),[(gsk3,axin)],global_attack,on,'7d').

% RULE 7e: If APCp interacts with b-catenin,
% then b-catenin can
%          interact with axinp.
%          (These steps describe the formation of the "mature"
%           destruction complex.)
rule((axin,bcat),[(apc,bcat),(apc,apcp),(axin,axinp),(bcat,bcat)],
    global_attack,on,'7d_1').
rule((axin,bcat),[not (axin,axinp), (axin,pp1)],

    global_attack,off,'7d_2').


% RULE 8a: If CK1a interacts with axin, and axin interacts
%          with b-catenin, then CK1a interacts with b-catenin.
```

```
rule((ck1a,bcat),[(axin,ck1a),(axin,bcat),(axin,axinp),(bcat,bcat)],
    global_attack,on,'8a').

% RULE 8b: If CK1a interacts with b-catenin, then b-catenin is
%          modified to form b-catp [=b-cat*] (that is CK1a
%          phosphorylates b-catenin).
%           Remark: Formation of b-catp has no influence on other
%          interactions in the destruction complex.
rule((bcat,bcatp),[(ck1a,bcat),(bcat,bcat)],global_attack,on,'8b').

% RULE 8c: In the presence of the mature destruction complex, GSK3 can
%          interact with b-catp and modify it into b-catpp [=b-cat**]
%          (that is GSK phosphorylates b-catp a total of three times).
rule((gsk3,bcat),[(axin,axinp),(axin,bcat),(axin,apc),(apc,bcat),

    (apc,gsk3),(bcat,bcat)],global_attack,on,'8c_1').
rule((bcat,bcatpp),[(gsk3,bcat),(bcat,bcatp),

    (bcat,bcat)],global_attack,on,'8c_2').

%%%%%%%%%%%%%%%%%%%%%%%%%%%%%%%%%%%%%%%%%%%%%%%%%%%%%%%%%%%%%%%%%%%%%%%%%

%%% ----------------------------------------------------------------- %%%
%%%                 Inhibition of phosphorylation                     %%%


%%% ----------------------------------------------------------------- %%%
% RULE 8d: If b-catpp is formed,

%          b-catpp can interact with E1/2/3.
rule((bcat,e123),[(bcat,bcatpp),(gsk3,bcat),(bcat,bcat)],

    global_attack,on,'8d').
                           %%% or %%%

%%% ----------------------------------------------------------------- %%%
%%%                 Inhibition of ubiquitination                      %%%

%%% ----------------------------------------------------------------- %%%
% RULE 8d: If b-catpp is formed
%          and axin does not interact with
```

```
%          LRPp [=LRP*] at the membrane,

%          b-catpp can interact with E1/2/3.
rule((bcat,e123),[(bcat,bcatpp),(gsk3,bcat),(bcat,bcat),

    not (lrp,axin)],global_attack,on,'8d').
%%%%%%%%%%%%%%%%%%%%%%%%%%%%%%%%%%%%%%%%%%%%%%%%%%%%%%%%%%%%%%%%%%%%%%%%


% RULE 8e: If b-catpp interacts with E1/2/3,
%           b-catpp is modified into b-catUb.
rule((bcat,bcatub),[(bcat,e123),(bcat,bcatpp)],global_attack,on,'8e').

% RULE 8f: b-CatUb is degraded,
%           therefore b-catenin cannot accumulate.
rule((bcat,bcatfree),[(bcat,bcatub),(bcat,e123)],global_attack,off,'8f_1').
rule((bcat,bcat),[(bcat,bcatub),(bcat,e123)],global_attack,off,'8f_2').


% RULE 9: If b-catenin is accumulating and interacts with
%         JNK2, b-catenin can be translocated in the nucleus
%         and interact with Lef.
rule((bcat,lef),[(bcat,bcatfree),(jnk2,bcat)],
    global_attack,on,'9').


% RULE 10: If b-catenin in the nucleus interacts with Lef,
%        Lef interacts with the DNA.
rule((lef,dna),[(bcat,lef)],global_attack,on,'10').




% RULE 11: In presence of Wnt, Wnt, Fz and LRP form a
%          trimeric complex.
rule((wnt,fz),[(ligand,wnt)],global_attack,on,'11_1').
rule((wnt,lrp),[(ligand,wnt)],global_attack,on,'11_2').
rule((fz,lrp),[(ligand,wnt)],global_attack,on,'11_3').


% RULE 12: If Wnt interacts with LRP, LRP interacts with CK1g
```

```
%          and GSK3.
rule((lrp,ck1g),[(wnt,lrp)],global_attack,on,'12_1').
rule((lrp,gsk3),[(wnt,lrp)],global_attack,on,'12_2').


% RULE 13: If LRP interacts with CK1g/GSK3, LRP
%          is modified and activated to form LRPp [=LRP*].
rule((lrp,lrpp),[(lrp,ck1g),(lrp,gsk3)],
    global_attack,on,'13').


% RULE 14a: If the trimeric complex of Wnt, Fz, and LRP is
%           formed, GaGDP is transfered into GaGTP. Gbg still
%           interacts with Ga at this step (see RULE 2).
rule((ga,gdp),[(wnt,fz),(wnt,lrp),(fz,lrp),(fz,ga),(fz,gbg)],
    global_attack,off,'14a_1').
rule((ga,gtp),[(wnt,fz),(wnt,lrp),(fz,lrp),(fz,ga),(fz,gbg)],
    global_attack,on,'14a_2').

% RULE 14b: If Ga is transfered into GaGTP,
%        G dissociates into GaGTP and Gbg.
%        Both leave the Fz receptor.
rule((ga,gbg),[(ga,gtp)],global_attack,off,'14b_1').
rule((fz,gbg),[(ga,gtp)],global_attack,off,'14b_2').
rule((fz,ga),[(ga,gtp)],global_attack,off,'14b_3').
rule((gbg,gbg),[(ga,gtp)],global_attack,on,'14b_4').


% RULE 15a: GaGTP is hydrolyzed to GaGDP.
rule((ga,gdp),[(ga,gtp)],global_attack,on,'15a_1').
rule((ga,gtp),[(ga,gtp)],global_attack,off,'15a_2').

% RULE 15b: Inactivated GaGDP interacts with Gbg and Fz.
rule((fz,ga),[(ga,gdp)],global_attack,on,'15b_1').
rule((fz,gbg),[(ga,gdp)],global_attack,on,'15b_2').

% RULE 16a: If active GaGTP is released,
%        GaGTP interacts with dsh.
rule((ga,dsh),[(ga,gtp)],global_attack,on,'16a').

% RULE 16b: If GaGTP interacts with dsh,
```

```
%     dshp [=dsh*] is formed (this likely involves CK1e).
rule((dsh,dshp),[(ga,dsh)],global_attack,on,'16b').


%% RULES 17-19: In presence of Wnt, b-catenin degradation is inhibited.


% RULE 17a: If dshp is formed, dshp interacts with axin.
rule((dshp,axin),[(dsh,dshp)],global_attack,on,'17a').


%%%%%%%%%%%%%%%%%%%%%%%%%%%%%%%%%%%%%%%%%%%%%%%%%%%%%%%%%%%%%%%%%%%%%%%%%

%%% ----------------------------------------------------------------- %%%
%%%                 Inhibition of phosphorylation                     %%%


%%% ----------------------------------------------------------------- %%%
% RULE 17b: If dshp is formed, dshp can interact with GBP.
rule((dsh,gbp),[(dsh,dshp)],global_attack,on,'17b').

% RULE 17c: If dshp interacts with axin and GBP,
%           GBP interacts with GSK3.
rule((gbp,gsk3),[(dsh,dshp),(dshp,axin),(dsh,gbp)],
    global_attack,on,'17c').


% RULE 17d: If GSK3 interacts with GBP, then GSK3 does
%           not interact with axin, APC or b-catenin.
rule((gsk3,axin),[(gbp,gsk3)],global_attack,off,'17d_1').
rule((apc,gsk3),[(gbp,gsk3)],global_attack,off,'17d_2').
rule((gsk3,bcat),[(gbp,gsk3)],global_attack,off,'17d_3').

                           %%% or %%%

%%% ----------------------------------------------------------------- %%%
%%%                 Inhibition of ubiquitination                      %%%

%%% ----------------------------------------------------------------- %%%
%%% no rules 17b-d
%%%%%%%%%%%%%%%%%%%%%%%%%%%%%%%%%%%%%%%%%%%%%%%%%%%%%%%%%%%%%%%%%%%%%%%%%
```

```
% RULE 18a: If dshp is formed, dshp can interact with Fz.
rule((fz,dsh),[(dsh,dshp)],global_attack,on,'18a').

% RULE 18b: If phoshorylated axinp interacts with phosphorylated dshp,
%           axinp can interact with phosphorylated LRPp.
rule((lrp,axin),[(lrp,lrpp),(axin,axinp),(dshp,axin),not (axin,pp1)],
    global_attack,on,'18b_1').
rule((lrp,axin),[not (axin,axinp), (axin,pp1)],global_attack,off,'18b_2').


% RULE 18c: If dshp interacts with Fz and axin,

%           and LRPp interacts with Fz and axin,
%           then axin interacts with protein phosphatase-1 (PP1).
rule((axin,pp1),[(dsh,dshp),(fz,dsh),(dshp,axin),
    (lrp,axin),(fz,lrp)],global_attack,on,'18c').

%%%%%%%%%%%%%%%%%%%%%%%%%%%%%%%%%%%%%%%%%%%%%%%%%%%%%%%%%%%%%%%%%%%%%%%%%

%%% ----------------------------------------------------------------- %%%
%%%                 Inhibition of phosphorylation                     %%%


%%% ----------------------------------------------------------------- %%%
% RULE 18d: If phosphorylated axin interacts with PP1,
%           axin is dephosphorylated

%           (i.e. it cannot interact with b-catenin).
%           Remark: Consequence of RULE 18d is, that b-catenin accumulates;
%                   RULES 9 and 10 will become effective!!!
rule((axin,axinp),[(axin,pp1),(lrp,axin)],global_attack,off,'18d').




% RULE 19a: If the destruction is inhibited and b-catenin is not degraded,
%           b-catenin can accumulate.
%           Remark: RULE 19a now also assumes
%           a positive influence of Rac on the amount of free
```

```
%           b-catenin, that is necessary for the model to fit
%           the experimental data obtained in this study.
rule((bcat,bcatfree),[(bcat,bcat),not (gsk3,bcat),not (axin,bcat),(rac,bcat)],
    global_attack,on,'19a').

                           %%% or %%%

%%% ---------------------------------------------------------------- %%%
%%%                 Inhibition of ubiquitination                     %%%

%%% ---------------------------------------------------------------- %%%
% RULE 18d: If axin interacts with LRPp, E1/2/3 dissociates from b-catenin.

%           Remark: Consequence of RULE 18d is, that b-catenin accumulates;
%                   RULES 9 and 10 will become effective!!!
rule((bcat,e123),[(lrp,axin),(lrp,lrpp)],global_attack,off,'18d_1').
rule((bcat,bcatub),[(lrp,axin),(lrp,lrpp)],global_attack,off,'18d_2').


% RULE 19a: If E/1/2/3 dissociates from the destruction complex upon
%          Wnt stimulation and b-catenin thus is not degraded,
%          b-catenin can accumulate.
%          Remark: RULE 19a now also assumes a positive influence of
%          Rac on the amount of free b-catenin, that is necessary for
%          the model to fit the experimental data obtained in this study.
rule((bcat,bcatfree),[(bcat,bcat),not (bcat,e123),not (bcat,bcatub),
    (rac,bcat)],global_attack,on,'19a').

%%%%%%%%%%%%%%%%%%%%%%%%%%%%%%%%%%%%%%%%%%%%%%%%%%%%%%%%%%%%%%%%%%%%%%%%%

% RULE 19b: If b-catenin accumulates, the high b-catenin concentration
%          will re-activate the destruction complex.
rule((gsk3,bcat),[(bcat,bcatfree)],global_attack,on,'19b_1').
rule((axin,bcat),[(bcat,bcatfree)],global_attack,on,'19b_2').
rule((ck1a,bcat),[(bcat,bcatfree)],global_attack,on,'19b_3').
rule((apc,bcat),[(bcat,bcatfree)],global_attack,on,'19b_4').


% RULE 20: If one of the scaffold proteins axin or APC is
%          missing, no destruction complex can be formed.
rule((apc,bcat),[not (axin,bcat),not (axin,apc),
```

```
    not (gsk3,axin),not (axin,ck1a)],
    global_attack,off,'20_1').
rule((gsk3,bcat),[not (axin,bcat),not (axin,apc),
    not (gsk3,axin),not (axin,ck1a)],
    global_attack,off,'20_2').
rule((ck1a,bcat),[not (axin,bcat),not (axin,apc),
    not (gsk3,axin),not (axin,ck1a)],
    global_attack,off,'20_3').
rule((axin,bcat),[not (apc,bcat),not (axin,apc),
    not (apc,gsk3)], global_attack,off,'20_4').
rule((gsk3,bcat),[not (apc,bcat),not (axin,apc),
    not (apc,gsk3)],global_attack,off,'20_5').
rule((ck1a,bcat),[not (apc,bcat),not (axin,apc),
    not (apc,gsk3)],global_attack,off,'20_6').


% RULE 21: Independent of the presence or absence of Wnt,
%          b-catenin is newly snthesized.
rule((bcat,bcat),[(new,bcat)],syn_rate,on,'21').





%%% Rules Wnt/JNK %%%

% RULE j5: In the presence of Wnt, Wnt interacts with
%          Fz and Ror2.
rule((wntj,fzj),[(ligandj,wntj)],global_attack,on,'j5_1').
rule((wntj,ror2),[(ligandj,wntj)],global_attack,on,'j5_2').


% RULE j6a: If Wnt interacts with Fz, GaGDP is transferred
%           into GaGTP. Gbg still interacts with Ga at this
%           step.
rule((gaj,gdp),[(wntj,fzj),(fzj,gaj),(fzj,gbgj)],
    global_attack,off,'j6a_1').
rule((gaj,gtp),[(wntj,fzj),(fzj,gaj),(fzj,gbgj)],
    global_attack,on,'j6a_2').

% RULE j6b: If GaGDP is transfered into GaGTP,
```

```
%        G dissociates into GaGTP and Gbg.
%        Both leave the receptor.
rule((gaj,gbgj),[(gaj,gtp)],global_attack,off,'j6b_1').
rule((fzj,gbgj),[(gaj,gtp)],global_attack,off,'j6b_2').
rule((fzj,gaj),[(gaj,gtp)],global_attack,off,'j6b_3').
rule((gbgj,gbgj),[(gaj,gtp)],global_attack,on,'j6b_4').


% RULE j7a: GaGTP is hydrolyzed to GaGDP.
rule((gaj,gdp),[(gaj,gtp)],global_attack,on,'j7a_1').
rule((gaj,gtp),[(gaj,gtp)],global_attack,off,'j7a_2').

% RULE j7b: Inactivated GaGDP interacts with Gbg and Fz.
rule((fzj,gaj),[(gaj,gdp)],global_attack,on,'j7b_1').
rule((fzj,gbgj),[(gaj,gdp)],global_attack,on,'j7b_2').


% RULE j8: If active GaGTP is released,
%       GaGTP interacts with dsh.
rule((gaj,dshj),[(gaj,gtp)],global_attack,on,'j8').


% RULE j9: If G dissociates into GaGTP and Gbg,
%       released Gbg can interact with PLCb.
rule((gbgj,plcbj),[(gaj,gtp),(gbgj,gbgj)],
    global_attack,on,'j9').


% RULE j10: If free Gbg interacts with and activates PLCb,
%           PLCb forms DAG.
rule((plcbj,dag),[(gbgj,plcbj)],global_attack,on,'j10').


% RULE j11a: DAG can interact with PKCd.
rule((dag,pkcd),[(plcbj,dag)],global_attack,on,'j11a').
% RULE j11b: If DAG interacts with PKCd,
%     PKCd is activated to form PKCdp [=PKCd*].
rule((pkcd,pkcdp),[(plcbj,dag),(dag,pkcd)],
    global_attack,on,'j11b').
```

```
% RULE j12: If PKCd is activated by DAG to form PKCdp,
%     PKCd can interact with dsh.
rule((pkcd,dshj),[(dag,pkcd),(pkcd,pkcdp)],
    global_attack,on,'j12_1').


% RULE j13: If Wnt interacts with Fz and Rror2,
%           Ror2 interacts with Fz.
rule((fzj,ror2),[(wntj,fzj),(wntj,ror2)],
    global_attack,on,'j13').


% RULE j14: If Fz interacts with Ror2, Fz interacts with dsh.
rule((fzj,dshj),[(fzj,ror2)],global_attack,on,'j14').


% RULE j15: If dsh interacts with Fz and dsh interacts with
%        GaGTP and dsh interacts with PKCdp,
%     dsh is activated to form dshpp [=dsh**].
rule((dshj,dshjpp),[(fzj,dshj),(gaj,dshj),(pkcd,dshj)],
    global_attack,on,'j12_2').


% RULE j16: If dshpp is formed, dsh can interact with Rac.
rule((dshj,rac),[(dshj,dshjpp)],global_attack,on,'j12_3').


% RULE j17a: If dsh interacts with Rac, Rac is activated to
%            form RacGTP.
rule((rac,gtp),[(dshj,rac)],global_attack,on,'j17a_1').
rule((rac,gdp),[(dshj,rac)],global_attack,off,'j17a_2').

% RULE j17b: Only active, GTP-bound Rac can interact with
%            JNK1 and JNK2 and influence b-catenin.
%            Remark: RULE j17b includes a hypothetical
%            connection from Rac to b-catenin (possibly
%            via DOCK4, but not including JNK) that is
%            necessary for the model to fit the experimental
%            data obtained in this study.
rule((rac,jnk1),[(rac,gtp)],global_attack,on,'j17b_1').
rule((rac,jnk2),[(rac,gtp)],global_attack,on,'j17b_2').
```

```
rule((rac,bcat),[(rac,gtp)],global_attack,on,'j17b_3').


% RULE j18: If Rac interacts with JNK1 and JNK2, they are
%        activated to form JNK1p [=JNK1*] and JNK2p [=JNK2*].
rule((jnk1,jnk1p),[(rac,jnk1)],global_attack,on,'j18_1').
rule((jnk2,jnk2p),[(rac,jnk2)],global_attack,on,'j18_2').


% RULE j19a: If JNK1 and JNK2 are activated,
%         they can interact with b-catenin.
rule((jnk1,bcat),[(jnk1,jnk1p),(bcat,bcat),not (bcat,bcatub)],
    global_attack,on,'j19a_1').
rule((jnk2,bcat),[(jnk2,jnk2p),(bcat,bcatfree)],
    global_attack,on,'j19a_2').

% RULE j19b: If JNK2 interacts with free b-catenin,
%     free b-catenin can interact with Lef.
%     -> ProbLog-Code see RULE 9

% RULE j19c: If JNK1 is activated, JNK1 can activate GSK3.
rule((jnk1,gsk3),[(jnk1,jnk1p)],global_attack,on,'j19c').

% RULE j19d: If JNK1 interacts with b-catenin and GSK3, and
%         b-catenin is in the destruction complex,
%         b-catenin is phosphorylated by GSK3.
rule((gsk3,bcat),[(jnk1,bcat),(jnk1,gsk3),(apc,bcat),
    (axin,bcat)],global_attack,on,'j19d').

:- init.
:- evaluation(300).
```

2.3.3 Wnt model feedbacks

The ProbRules model of Wnt signaling contains a range of feedbacks. Fig. N11A contains all 69 interactions of the model represented as boxed rectangles. Each interaction in the Boolean formula of a rule targeting an interaction was depicted by an arrow. We found several subgraphs consisting of interactions that are all connected via loops to each other. The largest 5 are highlighted by colors. Fig. N11B exemplifies the relations between the interactions in the largest subgraph.

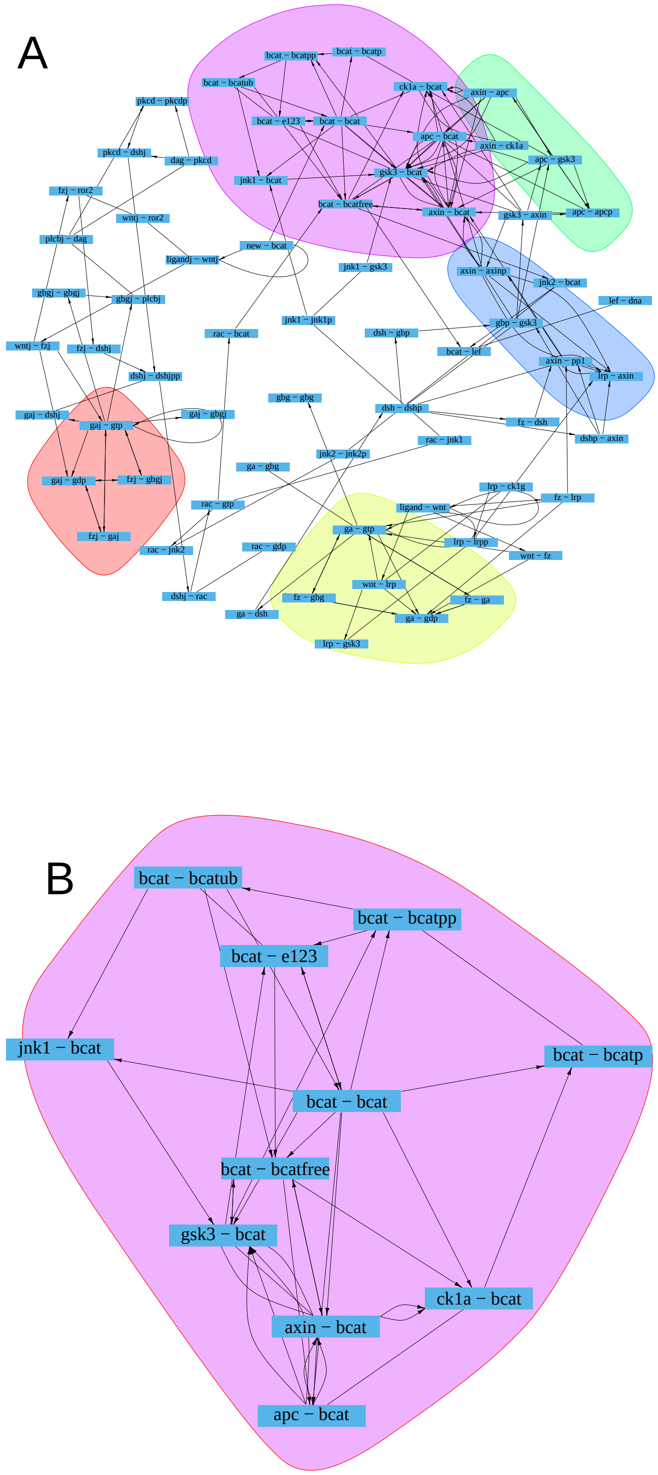

Figure N11: (A) Rectangles represent interactions in the ProbRules model of Wnt signaling. In highlighted subgraphs all interactions are involved in feedback loops. (B) The largest highlighted subgraph: β-catenin interactions with the destruction complex.

2.3.4 Model dynamics and robustness

Reaching a stable state before Wnt stimulation

Our ProbRules model of Wnt signaling includes an initial maturation phase of the destruction complex. In this phase, the complex is not yet fully stable and thus also not yet fully active. During this initial phase of complex stabilization the output of the Wnt model shows an initial peak until the destruction complex is fully active and the output reaches a stable state after about 75 time steps.

This is demonstrated in Fig. N12. However, this initial stabilization phase cannot be captured with experimental measurements in the used cell culture system where we always have to assume the presence of some fully mature destruction complexes in the absence of Wnt stimulation. In order to start our simulations of Wnt signal transduction from these defined conditions, we supplied the Wnt stimulus in our model only after 100 time steps. Additionally, for all further analyses we will concentrate on the network behavior after this initial stabilization phase and provide the simulation results starting from time step 76.

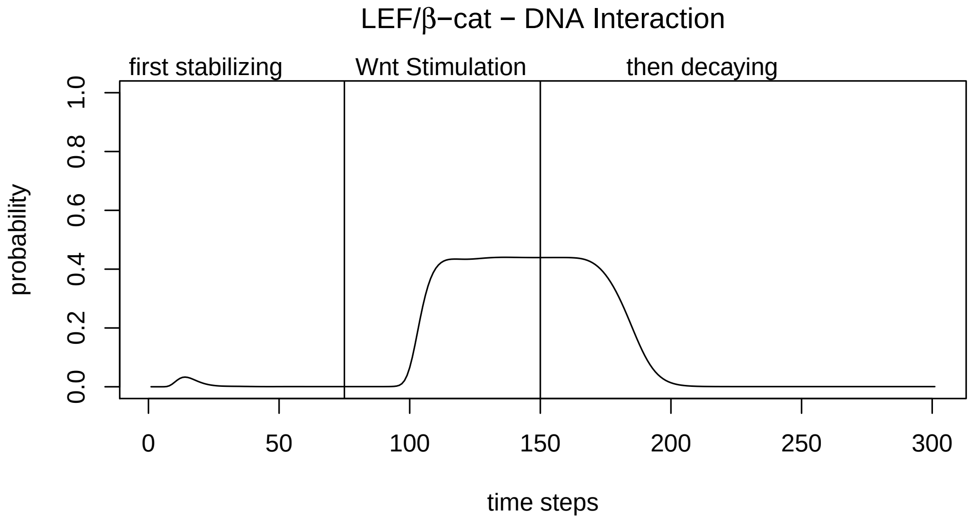

Figure N12: The output shows a small initial peak before Wnt stimulation due to a not fully established destruction complex.

Effect of the decay speed of the input signal

The input signal to the Wnt signaling network model was specified by predetermining the probabilities of Wnt ligand edges. The stimulus effectively consisted of a square pulse followed by an exponential decay:

$$p_t(\texttt{ligand}, \texttt{wnt}) = \begin{cases} 1 & \text{if } t \in [t_0, t_k) \\ e^{-(t-t_k) \, * \, ln \, \frac{1}{1-s}} & \text{otherwise} \end{cases}$$

with $t_0 = 100$, $t_k = 175$ and $s = 0.02$.

In order to investigate the influence of the decay speed (s) of the input signal, we ran the simulation using a range of values for the corresponding parameter, i.e. the input decay rate (in the model implementation, the corresponding parameter is called `slow_attack`) . Fig. N13 shows the dynamics of the input interaction depending on the decay speed. Fig. N14 shows the resulting behavior of the output interaction (LEF/β-catenin - DNA). Although low values of `slow_attack` lead to a decrease of the decay speed at the input, for moderate and higher values of `slow_attack` we did not see a qualitative difference.

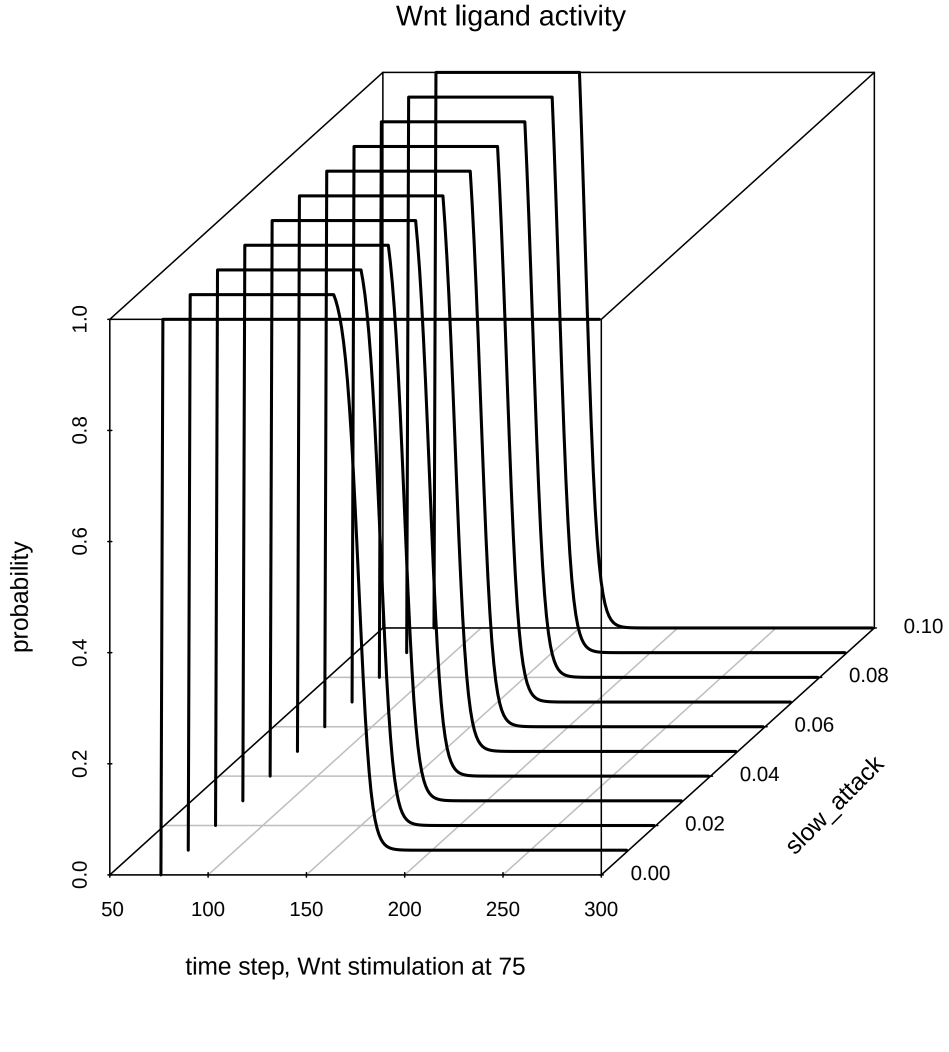

Figure N13: Illustration of the model input signal for different input decay rates

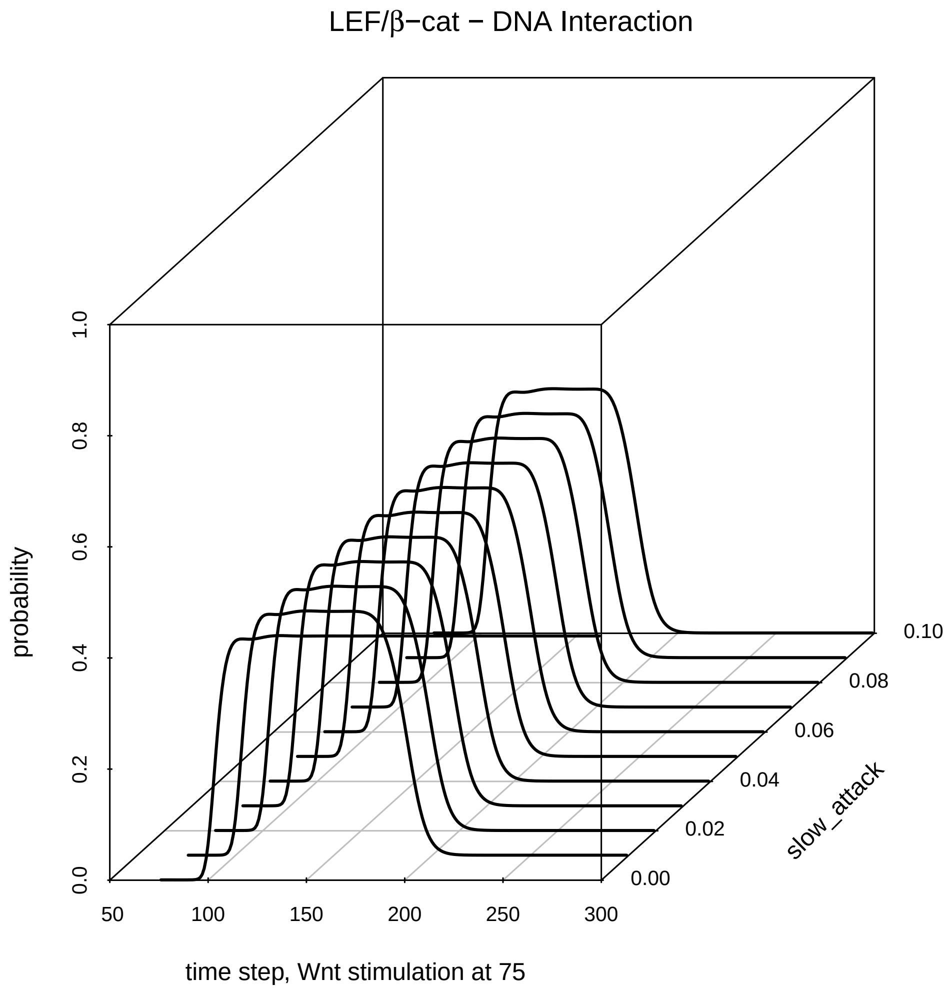

Figure N14: Time courses of the output signal for different input decay rates

Effect of the β-catenin synthesis rate

In a further analysis, we investigated the influence of the β-catenin synthesis rate on the output signal. Therefore, we ran the simulation using a range of values for the corresponding parameter (called `syn_rate` in the model implementation). Fig. N15 shows the resulting behavior of the output interaction (LEF/β-catenin - DNA). Here, turning off β-catenin synthesis ($\texttt{syn\_rate} = 0$) leads to a lower response. The response is not completely abolished as a certain basal amount of β-catenin is assumed to be present initially in our model to mimic the experimental situation in our cell culture system. There, a certain basal amount of β-catenin is also present initially [74, 75]. As the behavior at the output does not change over a wide range ($0.03...0.3$) of the parameter `syn_rate`, we chose an intermediate value (0.15) for the parameter in our further investigations.

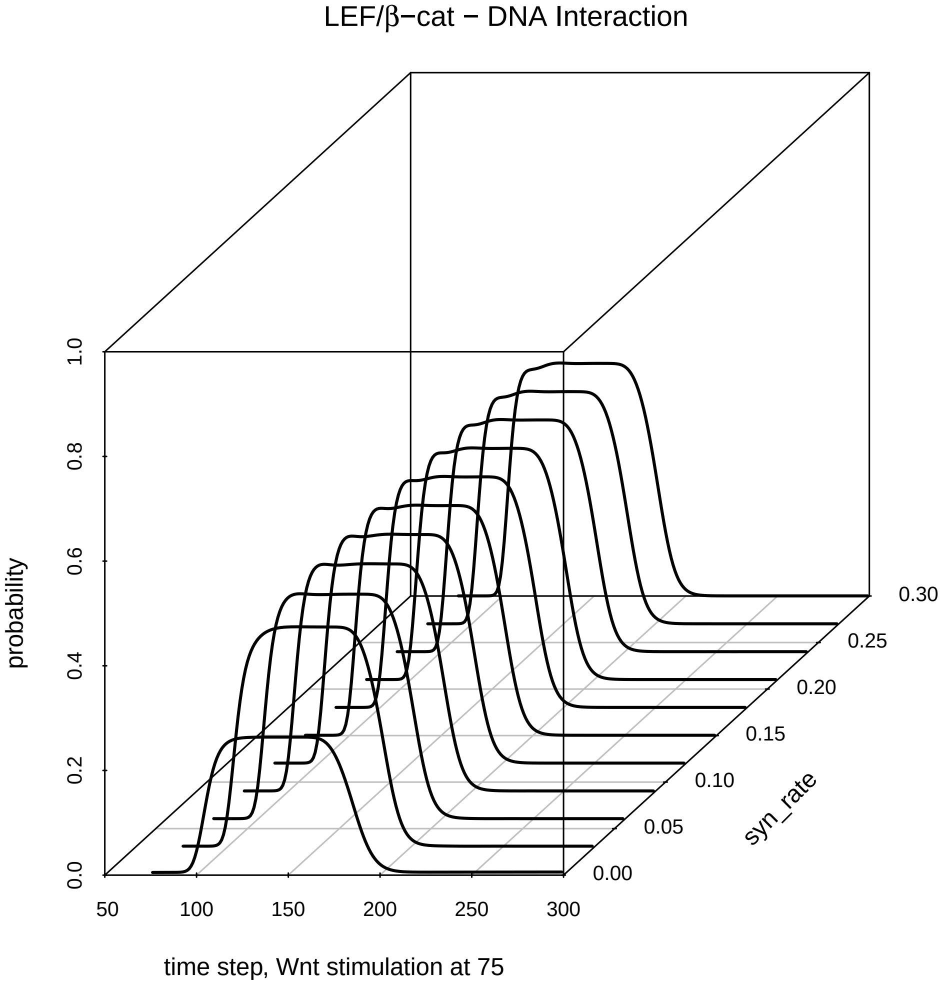

Figure N15: Time courses of the output interaction (LEF/β-catenin - DNA) for different β-catenin synthesis rates

Effects of additional rules

In this investigation, the original ProbRules model of Wnt signaling was perturbed systematically by adding artificial rules. Each of these rules is activated by one of the existing interactions in the network or a constitutively present interaction intended for permanent activation. They act on each of the non-input interactions in the network by either driving them towards full presence or full absence. This procedure results in 9380 new models, which were simulated and compared to the original specification.

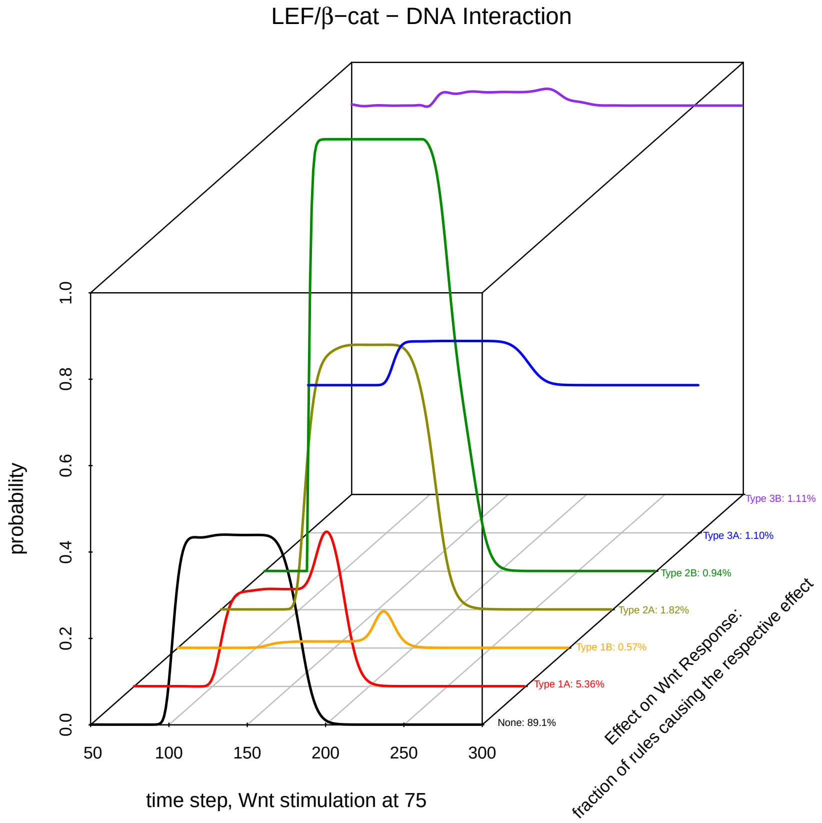

Figure N16: Effects of a complete exploration by adding rules. Besides the original behavior, three major types of aberrant behavior were observed, each of which is shown in the plot by means of two representative curves. Additionally, for each type the percentage of additional rules inducing the respective behavior is given.

As mentioned in the main text, 89% of the 9830 added rules did not induce significant changes in the transcriptional Wnt response, i.e. after addition of each of these 8358 rules, the output of the modified models showed basically the same behavior as in the original model. Each of the remaining 11% of additional rules, on the other hand, induced some change in the transcriptional Wnt response. According to the elicited change in the transcriptional response these 1022 rules can be roughly divided in three types (see also main text Fig. 6D, reproduced here as Fig. N16), which we want to discuss in the following:

Type 1: Rules of this type induce a reduction of the transcriptional Wnt response. Of the 576 rules of this type, 503 only reduce the strength of the signal (Type 1A), while 53 almost completely abolish the output signal (Type 1B). The observed reduction or elimination of the output signal can be caused through the inhibition of a target edge that is required for signal transduction or the activation of a target edge that inhibits signal transduction. For example, a reduction of the signal was caused by the addition of a rule through which the presence of the Wnt ligand induces the interaction of GSK3-β with β-catenin (example "Type 1A" in Fig. N16). An almost complete elimination of the output signal was for example caused by the addition of a rule through which the presence of the Wnt ligand inhibits the phosphorylation of dsh (example "Type 1B" in Fig. N16).

Type 2: Rules of this type induce an increase of the signal strength. An increase of the signal strength can be caused for example by introducing a shortcut through which an upstream source edge directly activates the output edge or by introducing a positive feedback loop through which an upstream target edge is activated by a downstream source edge. The introduced shortcut or feedback loop then leads to a stronger up-regulation and/or slower down-regulation of the output signal. Of the 259 rules of this type, 171 cause only a moderate increase of the signal strength (Type 2A), while 88 rules induce a strongly increased output signal (Type 2B). A moderate increase (Type 2A) was caused for example by addition of a rule through which the interaction between TCF/LEF and the DNA feeds back to induce the interaction between JNK2 and β-catenin (example "Type 2A" in Fig. N16). A strongly increased output signal (Type 2B), on the other hand, was caused for example by addition of a rule through which the presence of the Wnt ligand directly induces the interaction between β-catenin and TCF/LEF (example "Type "2A" in Fig. N16).

Type 3: Rules of this type induce TCF-dependent transcriptional activity independent of the Wnt stimulation. This Wnt-independent transcriptional activity can occur for example, if a source interaction that is present in the absence of Wnt activates a target interaction that in turn can activate downstream signaling. In contrast to rules of Type 2, in this case, the Wnt signal is not required for initiation of the response. Depending on the source and target edges, the strength of the observed effect can vary considerably. Such a Wnt-independent transcriptional activity was caused for example by addition of a rule through which the interaction between axin and CK1α induces the phosphorylation of dsh (example "Type 3A" in Fig. N16) or a rule through which an interaction between axin and β-catenin induces the presence of free β-catenin (example "Type 3B" in Fig. N16).

Strikingly, of the 67 interactions that occur as target edges in the additional rules (i.e. all network interactions except the two input signals), only 20 were observed to be involved in rules that caused abnormal network behavior. Rules in which the remaining 47 edges appeared as target edges did not affect the network behavior, irrespective of the source edge involved. Such sensitive target interactions were, besides the LEF-DNA interaction and the free β-catenin, mainly components of the destruction complex, the Wnt-receptor complex and interactions involving the heterotrimeric G proteins.

3 Supplementary Methods

3.1 StealthRNA/siRNA sequences

APC	Stealth 1	HSS100547	AAAGGAUGGAAUCUGAAUCAGACGA
	Stealth 2	HSS100548	CCCACCUAAUCUCAGUCCCACUAUA
LRP6	Stealth 1	HSS106153	ACGCAGCAUUGAGCGUGCCAACAAA
	Stealth 2	HSS106154	AAUACUAUCCGGUUAGCACCUGAGA
	Stealth 3	HSS106155	CAAAUCAGAUGAGAAGAACUGUGAA
RAC1	Stealth 1	VHS40447	AAAGACAGUAGGGAUAUAUUCUCCA
	Stealth 2	VHS40448	AGGGUCUAGCCAUGGCUAAGGAGAU
JNK1	Stealth 1	VHS40722	AUAAGAACUAGCUCUCUGUAGGCCC
	Stealth 2	VHS40724	AUCUAGCUCCAUCUGAAUCACUUGG
JNK2	Stealth 1	VHS40726	AUGCAGCACAAACAAUCCCUUGGGC
	Stealth 2	VHS40729	GCCAACUGUGAGGAAUUAUGUCGAA
AllStars	Negative control siRNA		UGCUGACUCCAAAGCUCUG

3.2 RT-PCR primer sequences

APC 5'-GTCCCTCCGTTCTTATGGAAG-3'
5'-ACTCTGATTTGCCTTGCTTCA-3'

LRP6 5'-GCGTGAAATCCATTCTGACAT-3'
5'-AGCCTTCCACAGGATCGTAAT-3'

RAC1 5'-AACCTTTGTACGCTTTGCTCA-3'
5'-ATCCTCCACGTCTGTGAACGA-3'

JNK1 5'-CTGTGTGGAATCAAGCACCTT-3'
5'-CTTGTCGAACCTTGTGGTACA-3'

JNK2 5'-CAAGTCGCAGACTCAACCTTC-3'
5'- CCAATAAGTGTACCTCGACCT-3'

GAPDH 5'-AGCCACATCGCTCAGACACC-3'
5'-GTACTCAGCGGCCAGCATCG-3'

4 Supplementary References

[1] Bollobás, B., *Modern Graph Theory*, (Springer, New York1998), ISBN 0387984887.

[2] Adams, E. W., *A Primer of Probability Logic*, (Center for the Study of Language and Information, Stanford1996).

[3] Kimmig, A., Demoen, B., Raedt, L. D., Costa, V. S., & Rocha, R., On the implementation of the probabilistic logic programming language ProbLog, *Theory and Practice of Logic Programming* **11**, 235–262, 2011.

[4] Raedt, L. D., Frasconi, P., Kersting, K., & Muggleton, S., eds., *Probabilistic Inductive Logic Programming - Theory and Applications*, vol. 4911 of *Lecture Notes in Computer Science*, (Springer, Berlin2008).

[5] Getoor, L. & Taskar, B., eds., *Introduction to Statistical Relational Learning*, (MIT Press, Cambridge2007).

[6] Bratko, I., *Prolog Programming for Artificial Intelligence*, (Addison Wesley, New York1990), 2nd Edition.

[7] Thon, I., Landwehr, N., & Raedt, L. D., Stochastic relational processes: Efficient inference and applications, *Machine Learning* **82**, 239–272, 2011.

[8] Blackburn, P., Bos, J., & Striegnitz, K., *Learn Prolog Now!*, vol. 7 of *Texts in Computing*, (College Publications, London2006), ISBN 1-904987-17-6.

[9] Alon, U., *Introduction to Systems Biology: Design Principles of Biological Circuits*, (Chapman & Hall/CRC, London2006).

[10] Ingram, P. J., Stumpf, M. P. H., & Stark, J., Network motifs: structure does not determine function, *BMC Genomics* **7**, 108, 2006.

[11] Bhanot, P. *et al.*, A new member of the frizzled family from Drosophila functions as a Wingless receptor, *Nature* **382**, 225–230, 1996, ISSN 0028-0836, 1476-4687, doi:10.1038/382225a0.

[12] Yang-Snyder, J., Miller, J. R., Brown, J. D., Lai, C. J., & Moon, R. T., A frizzled homolog functions in a vertebrate Wnt signaling pathway, *Current biology* **6**, 1302–1306, 1996, ISSN 0960-9822.

[13] Hsieh, J. C., Rattner, A., Smallwood, P. M., & Nathans, J., Biochemical characterization of Wnt-frizzled interactions using a soluble, biologically active vertebrate Wnt protein, *Proceedings of the National Academy of Sciences of the United States of America* **96**, 3546–3551, 1999, ISSN 0027-8424.

[14] Rulifson, E. J., Wu, C. H., & Nusse, R., Pathway specificity by the bifunctional receptor frizzled is determined by affinity for wingless, *Molecular Cell* **6**, 117–126, 2000, ISSN 1097-2765.

[15] Wu, C.-h. & Nusse, R., Ligand Receptor Interactions in the Wnt Signaling Pathway in *Drosophila*, *Journal of Biological Chemistry* **277**, 41762–41769, 2002, ISSN 0021-9258, 1083-351X, doi: 10.1074/jbc.M207850200.

[16] Wehrli, M. *et al.*, arrow encodes an LDL-receptor-related protein essential for Wingless signalling, *Nature* **407**, 527–530, 2000, ISSN 0028-0836, doi:10.1038/35035110.

[17] Pinson, K. I., Brennan, J., Monkley, S., Avery, B. J., & Skarnes, W. C., An LDL-receptor-related protein mediates Wnt signalling in mice, *Nature* **407**, 535–538, 2000, ISSN 0028-0836, doi: 10.1038/35035124.

[18] Tamai, K. *et al.*, LDL-receptor-related proteins in Wnt signal transduction, *Nature* **407**, 530–535, 2000, ISSN 0028-0836, doi:10.1038/35035117.

[19] Kelly, O. G., Pinson, K. I., & Skarnes, W. C., The Wnt co-receptors Lrp5 and Lrp6 are essential for gastrulation in mice, *Development* **131**, 2803–2815, 2004, ISSN 0950-1991, doi:10.1242/dev.01137.

[20] Semënov, M. V. *et al.*, Head inducer Dickkopf-1 is a ligand for Wnt coreceptor LRP6, *Current biology* **11**, 951–961, 2001, ISSN 0960-9822.

[21] Cong, F., Schweizer, L., & Varmus, H., Wnt signals across the plasma membrane to activate the beta-catenin pathway by forming oligomers containing its receptors, Frizzled and LRP, *Development* **131**, 5103–5115, 2004, ISSN 0950-1991, doi:10.1242/dev.01318.

[22] Liu, T., Liu, X., Wang, H. Y., Moon, R. T., & Malbon, C. C., Activation of rat frizzled-1 promotes Wnt signaling and differentiation of mouse F9 teratocarcinoma cells via pathways that require Gα(q) and Gα(o) function, *The Journal of Biological Chemistry* **274**, 33539–33544, 1999, ISSN 0021-9258.

[23] Liu, X. *et al.*, Activation of a frizzled-2/beta-adrenergic receptor chimera promotes Wnt signaling and differentiation of mouse F9 teratocarcinoma cells via Gαo and Gαt, *Proceedings of the National Academy of Sciences of the United States of America* **96**, 14383–14388, 1999, ISSN 0027-8424.

[24] Liu, T. *et al.*, G protein signaling from activated rat frizzled-1 to the β-catenin-Lef-Tcf pathway, *Science* **292**, 1718–1722, 2001, ISSN 0036-8075, doi:10.1126/science.1060100.

[25] Penzo-Mendèz, A., Umbhauer, M., Djiane, A., Boucaut, J.-C., & Riou, J.-F., Activation of G$\beta\gamma$ signaling downstream of Wnt-11/Xfz7 regulates Cdc42 activity during Xenopus gastrulation, *Developmental Biology* **257**, 302–314, 2003, ISSN 0012-1606.

[26] Liu, X., Rubin, J. S., & Kimmel, A. R., Rapid, Wnt-induced changes in GSK3β associations that regulate β-catenin stabilization are mediated by Gα proteins, *Current biology* **15**, 1989–1997, 2005, ISSN 0960-9822, doi:10.1016/j.cub.2005.10.050.

[27] Katanaev, V. L., Ponzielli, R., Sémériva, M., & Tomlinson, A., Trimeric G protein-dependent frizzled signaling in Drosophila, *Cell* **120**, 111–122, 2005, ISSN 0092-8674, doi:10.1016/j.cell.2004.11.014.

[28] Quaiser, T., Anton, R., & Kühl, M., Kinases and G proteins join the Wnt receptor complex, *BioEssays: News and Reviews in Molecular, Cellular and Developmental Biology* **28**, 339–343, 2006, ISSN 0265-9247, doi:10.1002/bies.20386.

[29] Mao, J. *et al.*, Low-density lipoprotein receptor-related protein-5 binds to Axin and regulates the canonical Wnt signaling pathway, *Molecular Cell* **7**, 801–809, 2001, ISSN 1097-2765.

[30] Tamai, K. *et al.*, A mechanism for Wnt coreceptor activation, *Molecular Cell* **13**, 149–156, 2004, ISSN 1097-2765.

[31] Zeng, X. *et al.*, A dual-kinase mechanism for Wnt co-receptor phosphorylation and activation, *Nature* **438**, 873–877, 2005, ISSN 1476-4687, doi:10.1038/nature04185.

[32] Davidson, G. *et al.*, Casein kinase 1 gamma couples Wnt receptor activation to cytoplasmic signal transduction, *Nature* **438**, 867–872, 2005, ISSN 1476-4687, doi:10.1038/nature04170.

[33] Wong, H.-C. *et al.*, Direct binding of the PDZ domain of Dishevelled to a conserved internal sequence in the C-terminal region of Frizzled, *Molecular Cell* **12**, 1251–1260, 2003, ISSN 1097-2765.

[34] Cong, F., Schweizer, L., & Varmus, H., Casein kinase Iepsilon modulates the signaling specificities of dishevelled, *Molecular and Cellular Biology* **24**, 2000–2011, 2004, ISSN 0270-7306.

[35] Umbhauer, M. *et al.*, The C-terminal cytoplasmic Lys-Thr-X-X-X-Trp motif in frizzled receptors mediates Wnt/β-catenin signalling, *The EMBO journal* **19**, 4944–4954, 2000, ISSN 0261-4189, doi:10.1093/emboj/19.18.4944.

[36] Yanagawa, S.-i. *et al.*, Casein kinase I phosphorylates the Armadillo protein and induces its degradation in Drosophila, *The EMBO journal* **21**, 1733–1742, 2002, ISSN 0261-4189, doi:10.1093/emboj/21.7.1733.

[37] Cliffe, A., Hamada, F., & Bienz, M., A role of Dishevelled in relocating Axin to the plasma membrane during wingless signaling, *Current biology* **13**, 960–966, 2003, ISSN 0960-9822.

[38] Kitagawa, M. *et al.*, An F-box protein, FWD1, mediates ubiquitin-dependent proteolysis of β-catenin, *The EMBO journal* **18**, 2401–2410, 1999, ISSN 0261-4189, doi:10.1093/emboj/18.9.2401.

[39] Farr, G. H. *et al.*, Interaction among GSK-3, GBP, axin, and APC in Xenopus axis specification, *The Journal of Cell Biology* **148**, 691–702, 2000, ISSN 0021-9525.

[40] Salic, A., Lee, E., Mayer, L., & Kirschner, M. W., Control of β-catenin stability: reconstitution of the cytoplasmic steps of the wnt pathway in Xenopus egg extracts, *Molecular Cell* **5**, 523–532, 2000, ISSN 1097-2765.

[41] Hinoi, T. *et al.*, Complex formation of adenomatous polyposis coli gene product and axin facilitates glycogen synthase kinase-3 beta-dependent phosphorylation of beta-catenin and down-regulates beta-catenin, *The Journal of Biological Chemistry* **275**, 34399–34406, 2000, ISSN 0021-9258, doi:10.1074/jbc.M003997200.

[42] Ikeda, S. *et al.*, Axin, a negative regulator of the Wnt signaling pathway, forms a complex with GSK-3β and β-catenin and promotes GSK-3β-dependent phosphorylation of β-catenin, *The EMBO journal* **17**, 1371–1384, 1998, ISSN 0261-4189, doi:10.1093/emboj/17.5.1371.

[43] Kishida, S. *et al.*, Axin, a negative regulator of the wnt signaling pathway, directly interacts with adenomatous polyposis coli and regulates the stabilization of β-catenin, *The Journal of Biological Chemistry* **273**, 10823–10826, 1998, ISSN 0021-9258.

[44] Ikeda, T. *et al.*, Mutational analysis of the CTNNB1 (β-catenin) gene in human endometrial cancer: frequent mutations at codon 34 that cause nuclear accumulation, *Oncology Reports* **7**, 323–326, 2000, ISSN 1021-335X.

[45] Behrens, J. *et al.*, Functional interaction of an axin homolog, conductin, with β-catenin, APC, and GSK3β, *Science* **280**, 596–599, 1998, ISSN 0036-8075.

[46] Yamamoto, H. *et al.*, Axil, a member of the Axin family, interacts with both glycogen synthase kinase 3β and β-catenin and inhibits axis formation of Xenopus embryos, *Molecular and Cellular Biology* **18**, 2867–2875, 1998, ISSN 0270-7306.

[47] Yamamoto, H. *et al.*, Phosphorylation of axin, a Wnt signal negative regulator, by glycogen synthase kinase-3β regulates its stability, *The Journal of Biological Chemistry* **274**, 10681–10684, 1999, ISSN 0021-9258.

[48] Tolwinski, N. S. *et al.*, Wg/Wnt signal can be transmitted through arrow/LRP5,6 and Axin independently of Zw3/Gsk3β activity, *Developmental Cell* **4**, 407–418, 2003, ISSN 1534-5807.

[49] Kim, S.-E. *et al.*, Wnt stabilization of β-catenin reveals principles for morphogen receptor-scaffold assemblies, *Science* **340**, 867–870, 2013, ISSN 1095-9203, doi:10.1126/science.1232389.

[50] Liu, C. *et al.*, Control of β-catenin phosphorylation/degradation by a dual-kinase mechanism, *Cell* **108**, 837–847, 2002, ISSN 0092-8674.

[51] Ciechanover, A., The ubiquitin-proteasome pathway: on protein death and cell life, *The EMBO journal* **17**, 7151–7160, 1998, ISSN 0261-4189, doi:10.1093/emboj/17.24.7151.

[52] Aberle, H., Bauer, A., Stappert, J., Kispert, A., & Kemler, R., β-catenin is a target for the ubiquitin-proteasome pathway, *The EMBO Journal* **16**, 3797–3804, 1997, ISSN 0261-4189, doi:10.1093/emboj/16.13.3797.

[53] Behrens, J. *et al.*, Functional interaction of beta-catenin with the transcription factor LEF-1, *Nature* **382**, 638–642, 1996, ISSN 0028-0836, doi:10.1038/382638a0.

[54] Huber, O. *et al.*, Nuclear localization of β-catenin by interaction with transcription factor LEF-1, *Mechanisms of Development* **59**, 3–10, 1996, ISSN 0925-4773.

[55] Molenaar, M. *et al.*, XTcf-3 transcription factor mediates beta-catenin-induced axis formation in Xenopus embryos, *Cell* **86**, 391–399, 1996, ISSN 0092-8674.

[56] Buongiorno, P., Pethe, V. V., Charames, G. S., Esufali, S., & Bapat, B., Rac1 GTPase and the Rac1 exchange factor Tiam1 associate with Wnt-responsive promoters to enhance beta-catenin/TCF-dependent transcription in colorectal cancer cells, *Molecular Cancer* **7**, 73, 2008, ISSN 1476-4598, doi:10.1186/1476-4598-7-73.

[57] Gan, X.-q. *et al.*, Nuclear Dvl, c-Jun, β-catenin, and TCF form a complex leading to stabilization of beta-catenin-TCF interaction, *The Journal of Cell Biology* **180**, 1087–1100, 2008, ISSN 1540-8140, doi:10.1083/jcb.200710050.

[58] Wu, X. *et al.*, Rac1 activation controls nuclear localization of β-catenin during canonical Wnt signaling, *Cell* **133**, 340–353, 2008, ISSN 1097-4172, doi:10.1016/j.cell.2008.01.052.

[59] Upadhyay, G. *et al.*, Molecular association between beta-catenin degradation complex and Rac guanine exchange factor DOCK4 is essential for Wnt/β-catenin signaling, *Oncogene* **27**, 5845–5855, 2008, ISSN 1476-5594, doi:10.1038/onc.2008.202.

[60] Aoki, M., Hecht, A., Kruse, U., Kemler, R., & Vogt, P. K., Nuclear endpoint of Wnt signaling: neoplastic transformation induced by transactivating lymphoid-enhancing factor 1, *Proceedings of the National Academy of Sciences of the United States of America* **96**, 139–144, 1999, ISSN 0027-8424.

[61] van de Wetering, M. *et al.*, Armadillo coactivates transcription driven by the product of the Drosophila segment polarity gene dTCF, *Cell* **88**, 789–799, 1997, ISSN 0092-8674.

[62] Hsu, S. C., Galceran, J., & Grosschedl, R., Modulation of transcriptional regulation by LEF-1 in response to Wnt-1 signaling and association with beta-catenin, *Molecular and Cellular Biology* **18**, 4807–4818, 1998, ISSN 0270-7306.

[63] Hecht, A., Litterst, C. M., Huber, O., & Kemler, R., Functional characterization of multiple transactivating elements in β-catenin, some of which interact with the TATA-binding protein in vitro, *The Journal of Biological Chemistry* **274**, 18017–18025, 1999, ISSN 0021-9258.

[64] Vleminckx, K., Kemler, R., & Hecht, A., The C-terminal transactivation domain of beta-catenin is necessary and sufficient for signaling by the LEF-1/β-catenin complex in Xenopus laevis, *Mechanisms of Development* **81**, 65–74, 1999, ISSN 0925-4773.

[65] Bauer, A., Huber, O., & Kemler, R., Pontin52, an interaction partner of beta-catenin, binds to the TATA box binding protein, *Proceedings of the National Academy of Sciences of the United States of America* **95**, 14787–14792, 1998, ISSN 0027-8424.

[66] Kishida, M. *et al.*, Synergistic activation of the Wnt signaling pathway by Dvl and casein kinase Iϵ, *The Journal of Biological Chemistry* **276**, 33147–33155, 2001, ISSN 0021-9258, doi:10.1074/jbc.M103555200.

[67] Hino, S.-i., Michiue, T., Asashima, M., & Kikuchi, A., Casein kinase Iϵ enhances the binding of Dvl-1 to Frat-1 and is essential for Wnt-3a-induced accumulation of β-catenin, *The Journal of Biological Chemistry* **278**, 14066–14073, 2003, ISSN 0021-9258, doi:10.1074/jbc.M213265200.

[68] Angers, S. *et al.*, The KLHL12-Cullin-3 ubiquitin ligase negatively regulates the Wnt-beta-catenin pathway by targeting Dishevelled for degradation, *Nature Cell Biology* **8**, 348–357, 2006, ISSN 1465-7392, doi:10.1038/ncb1381.

[69] Li, L. *et al.*, Dishevelled proteins lead to two signaling pathways. Regulation of LEF-1 and c-Jun N-terminal kinase in mammalian cells, *The Journal of Biological Chemistry* **274**, 129–134, 1999, ISSN 0021-9258.

[70] Kishida, M. *et al.*, Axin prevents Wnt-3a-induced accumulation of β-catenin, *Oncogene* **18**, 979–985, 1999, ISSN 0950-9232, doi:10.1038/sj.onc.1202388.

[71] Lee, E., Salic, A., & Kirschner, M. W., Physiological regulation of β-catenin stability by Tcf3 and CK1epsilon, *The Journal of Cell Biology* **154**, 983–993, 2001, ISSN 0021-9525, doi:10.1083/jcb.200102074.

[72] Li, V. S. W. *et al.*, Wnt signaling through inhibition of β-catenin degradation in an intact Axin1 complex, *Cell* **149**, 1245–1256, 2012, ISSN 1097-4172, doi:10.1016/j.cell.2012.05.002.

[73] Kikuchi, A., Kishida, S., & Yamamoto, H., Regulation of Wnt signaling by protein-protein interaction and post-translational modifications, *Experimental & Molecular Medicine* **38**, 1–10, 2006, ISSN 1226-3613, doi:10.1038/emm.2006.1.

[74] Hernández, A. R., Klein, A. M., & Kirschner, M. W., Kinetic responses of β-catenin specify the sites of Wnt control, *Science* **338**, 1337–1340, 2012, ISSN 1095-9203, doi:10.1126/science.1228734.

[75] Lee, E., Salic, A., Krüger, R., Heinrich, R., & Kirschner, M. W., The roles of APC and Axin derived from experimental and theoretical analysis of the Wnt pathway, *PLoS biology* **1**, E10, 2003, ISSN 1545-7885, doi:10.1371/journal.pbio.0000010.

[76] Oishi, I. *et al.*, The receptor tyrosine kinase Ror2 is involved in non-canonical Wnt5a/JNK signalling pathway, *Genes to Cells: Devoted to Molecular & Cellular Mechanisms* **8**, 645–654, 2003, ISSN 1356-9597.

[77] Coso, O. A. *et al.*, The small GTP-binding proteins Rac1 and Cdc42 regulate the activity of the JNK/SAPK signaling pathway, *Cell* **81**, 1137–1146, 1995, ISSN 0092-8674.

[78] Minden, A., Lin, A., Claret, F. X., Abo, A., & Karin, M., Selective activation of the JNK signaling cascade and c-Jun transcriptional activity by the small GTPases Rac and Cdc42Hs, *Cell* **81**, 1147–1157, 1995, ISSN 0092-8674.

[79] Park, D., Jhon, D. Y., Lee, C. W., Lee, K. H., & Rhee, S. G., Activation of phospholipase C isozymes by G protein $\beta\gamma$ subunits, *The Journal of Biological Chemistry* **268**, 4573–4576, 1993, ISSN 0021-9258.

[80] Hughes, A. R. & Putney, J. W., Inositol phosphate formation and its relationship to calcium signaling, *Environmental Health Perspectives* **84**, 141–147, 1990, ISSN 0091-6765.

[81] Kinoshita, N., Iioka, H., Miyakoshi, A., & Ueno, N., PKC delta is essential for Dishevelled function in a noncanonical Wnt pathway that regulates Xenopus convergent extension movements, *Genes & Development* **17**, 1663–1676, 2003, ISSN 0890-9369, doi:10.1101/gad.1101303.

[82] Kühl, M. *et al.*, Antagonistic regulation of convergent extension movements in Xenopus by Wnt/β-catenin and Wnt/Ca2+ signaling, *Mechanisms of Development* **106**, 61–76, 2001, ISSN 0925-4773.

[83] Mikels, A. J. & Nusse, R., Purified Wnt5a protein activates or inhibits β-catenin-TCF signaling depending on receptor context, *PLoS biology* **4**, e115, 2006, ISSN 1545-7885, doi:10.1371/journal.pbio.0040115.

[84] Hikasa, H., Shibata, M., Hiratani, I., & Taira, M., The Xenopus receptor tyrosine kinase Xror2 modulates morphogenetic movements of the axial mesoderm and neuroectoderm via Wnt signaling, *Development* **129**, 5227–5239, 2002, ISSN 0950-1991.

[85] Moriguchi, T. *et al.*, Distinct domains of mouse dishevelled are responsible for the c-Jun N-terminal kinase/stress-activated protein kinase activation and the axis formation in vertebrates, *Journal of Biological Chemistry* **274**, 30957–30962, 1999, ISSN 0021-9258.

[86] Boutros, M., Paricio, N., Strutt, D. I., & Mlodzik, M., Dishevelled activates JNK and discriminates between JNK pathways in planar polarity and wingless signaling, *Cell* **94**, 109–118, 1998, ISSN 0092-8674.

[87] Habas, R., Dawid, I. B., & He, X., Coactivation of Rac and Rho by Wnt/Frizzled signaling is required for vertebrate gastrulation, *Genes & Development* **17**, 295–309, 2003, ISSN 0890-9369, doi:10.1101/gad.1022203.

[88] Habas, R., Kato, Y., & He, X., Wnt/Frizzled activation of Rho regulates vertebrate gastrulation and requires a novel Formin homology protein Daam1, *Cell* **107**, 843–854, 2001, ISSN 0092-8674.

[89] Fanto, M., Weber, U., Strutt, D. I., & Mlodzik, M., Nuclear signaling by Rac and Rho GTPases is required in the establishment of epithelial planar polarity in the Drosophila eye, *Current biology* **10**, 979–988, 2000, ISSN 0960-9822.

[90] Teramoto, H. *et al.*, Signaling from the small GTP-binding proteins Rac1 and Cdc42 to the c-Jun N-terminal kinase/stress-activated protein kinase pathway. A role for mixed lineage kinase 3/protein-tyrosine kinase 1, a novel member of the mixed lineage kinase family, *The Journal of Biological Chemistry* **271**, 27225–27228, 1996, ISSN 0021-9258.

[91] Liao, G. *et al.*, Jun NH2-terminal kinase (JNK) prevents nuclear beta-catenin accumulation and regulates axis formation in Xenopus embryos, *Proceedings of the National Academy of Sciences of the United States of America* **103**, 16313–16318, 2006, ISSN 0027-8424, doi:10.1073/pnas.0602557103.

[92] Hu, D. *et al.*, c-Jun N-terminal kinase 1 interacts with and negatively regulates Wnt/β-catenin signaling through GSK3β pathway, *Carcinogenesis* **29**, 2317–2324, 2008, ISSN 1460-2180, doi:10.1093/carcin/bgn239.
